# Mathematical modeling and comparison of protein size distribution in different plant, animal, fungal and microbial species reveals a negative correlation between protein size and protein number, thus providing insight into the evolution of proteomes

**DOI:** 10.1186/1756-0500-5-85

**Published:** 2012-02-01

**Authors:** Axel Tiessen, Paulino Pérez-Rodríguez, Luis José Delaye-Arredondo

**Affiliations:** 1 Departamento de Ingeniería Genética, CINVESTAV Irapuato, Irapuato, CP 36821, Mexico; 2 Colegio de Posgraduados, Texcoco, Mexico

## Abstract

**Background:**

The sizes of proteins are relevant to their biochemical structure and for their biological function. The statistical distribution of protein lengths across a diverse set of taxa can provide hints about the evolution of proteomes.

**Results:**

Using the full genomic sequences of over 1,302 prokaryotic and 140 eukaryotic species two datasets containing 1.2 and 6.1 million proteins were generated and analyzed statistically. The lengthwise distribution of proteins can be roughly described with a gamma type or log-normal model, depending on the species. However the shape parameter of the gamma model has not a fixed value of 2, as previously suggested, but varies between 1.5 and 3 in different species. A gamma model with unrestricted shape parameter described best the distributions in ~48% of the species, whereas the log-normal distribution described better the observed protein sizes in 42% of the species. The gamma restricted function and the sum of exponentials distribution had a better fitting in only ~5% of the species. Eukaryotic proteins have an average size of 472 aa, whereas bacterial (320 aa) and archaeal (283 aa) proteins are significantly smaller (33-40% on average). Average protein sizes in different phylogenetic groups were: Alveolata (628 aa), Amoebozoa (533 aa), Fornicata (543 aa), Placozoa (453 aa), Eumetazoa (486 aa), Fungi (487 aa), Stramenopila (486 aa), Viridiplantae (392 aa). Amino acid composition is biased according to protein size. Protein length correlated negatively with %C, %M, %K, %F, %R, %W, %Y and positively with %D, %E, %Q, %S and %T. Prokaryotic proteins had a different protein size bias for %E, %G, %K and %M as compared to eukaryotes.

**Conclusions:**

Mathematical modeling of protein length empirical distributions can be used to asses the quality of small ORFs annotation in genomic releases (detection of too many false positive small ORFs). There is a negative correlation between average protein size and total number of proteins among eukaryotes but not in prokaryotes. The %GC content is positively correlated to total protein number and protein size in prokaryotes but not in eukaryotes. Small proteins have a different amino acid bias than larger proteins. Compared to prokaryotic species, the evolution of eukaryotic proteomes was characterized by increased protein number (massive gene duplication) and substantial changes of protein size (domain addition/subtraction).

## Background

The biological function of a protein is determined by its tertiary, i.e., three-dimensional, structure, which in turn is influenced by its primary structure, i.e. its amino acid sequence. Besides the given order of amino acids (aa), the total length of a protein is also important for determining the tertiary structure of any polypeptide. The longer a protein is, the more options there are for accommodating multiple secondary structures and folding loops [1-3]. The statistical distribution of the sizes of proteins has been investigated by several groups in the past, although with a limited number of representative taxa or focused on prokaryotes. Comparison of average protein size in the proteomes of 5 archaeal, 15 bacterial and 2 eukaryotic species revealed marked differences of protein size [4]. A larger study compared also protein sizes in 16 archaeal, 67 bacterial and 5 eukaryotic species and came to a similar conclusion [5]. It is actually well established that eukaryotic proteins are on average, significantly longer than bacterial proteins, and these in turn are longer on average than archaeal proteins [4,5]. However, previous studies have not investigated whether there are protein size differences among eukaryotic organisms.

The fact that eukaryotic organisms have larger proteins than prokaryotic species has been interpreted as a true evolutionary trend towards an increase of protein size [4,5]. It has been postulated that the evolution of eukaryotic proteins was influenced by processes of fusion of single-function proteins into extended multifunctional and multi-domain proteins [5]. Fusion of domains of given size could be predicted to give rise to peaks of a multiple given size in the protein size histograms given the discontinuity of domain sizes and the limited number of different structural domain types. Fusion of domains increases the average size of proteins and this could potentially lead to a respective reduction of the number of individual proteins in the genome.

The evolutionary forces that have shaped protein number and size distributions in modern organisms are unknown. Some groups have tried to find answers based on theoretical models. According to the frequency of stop triplets in the genetic code (= 3/64), the expected size of an open reading frame (ORF) from a random DNA sequence should be on average 64 nucleotides long (~21 aa) [6]. However, since stop codons are biased towards the nucleotides T and A, the expected size also depends on the %GC content of the random sequence, varying between 14 aa (for 35% GC) and 31 aa (for 60% GC) [7]. According to a more detailed analysis of ORF statistics, non-coding DNA sequences are not fully random, but generate random ORF much longer than theoretically expected [8]. Nevertheless, a sharp cutoff is found at 100 triplets (~33 aa) [8]. Since most biologically active proteins are actually much larger than 50 aa, there must be a strong selective mechanism for maintaining the coding capacity of DNA (ORF length).

Two theories have been postulated to explain the relation between protein origin and size distribution: the starter-set and the random-origin hypotheses. The starter-set hypothesis assumes that proteins originated from a small set of starter sequences (functional domains) with lengths of 4 aa, 15 aa or 50 aa which were expanded by gene duplication and modification [9-13]. The premise of this hypothesis is that gene or exon duplication and fusion were essential from the very beginning of protein evolution for producing modern sequences of all organisms, including prokaryotic and eukaryotic species [14]. In contrast, the random-origin hypothesis assumes that proteins emerged first from a very large number of random heteropeptides [15-17]. The random-origin hypothesis assumes that the length of proteins were initially determined by the "start" and "end" signals that delimited the primitive genes and that were distributed randomly along nucleotide sequences. The random-origin hypothesis explains the appearance of large proteins alone by chance [14,18]. The starter set hypothesis assumes that primitive proteins were initially very small (< 50 aa) but biologically active, and by domain joining and gene fusion became gradually larger [14,18].

In order to discriminate between those evolutionary theories, is it important to know the frequency of small proteins in the genomes of different species. It is also relevant to determine the selective advantage of having numerous proteins (gene duplications) or proteins of larger size (biological functionality). Some researchers have started by investigating which is the best theoretical model that underlies protein size distributions. White (1994) examined 1,792 sequences and reported that prokaryotic and eukaryotic protein sets had a similar statistical length distribution that could be described by a gamma distribution with shape parameter equal to two or with a distribution that results from the sum of two exponential distributions [18]. A moderate fit to a gamma-type distribution was also found by [19] whereas [20] postulated a better fit to a log-normal distribution. Some have argued that a log-normal function was more appropriate because it describes distributions that arise from the product of many random independent events [20]. A stochastic model based on multiplicative processes has also been used to explain protein length distributions [21].

Several groups have postulated that the sequence distributions of all organisms are similar and that it is possible to describe them in terms of simple mathematical functions [14,18,20,22]. Considering that proteins increase in length by addition or duplication, the log-normal distribution has been said to be less appropriate [20]. Gamma distributions, on the other hand, result from the addition of random intervals and have been said to be more reasonable *a priori *[22].

A simple theory to explain why protein lengths can follow gamma-type distributions [18], assumes that (i) protein sequences have exponentially distributed random lengths; (ii) there is a length dependence of protein fold stability and potential for biochemical activity which greatly limits the number of small proteins (< 100 aa). This theory assumes that maximal protein size (> 1,000 aa) is limited by the very frequent occurrence of stop codons, whereas minimal size is determined by the more limited biological usefulness of small proteins (< 100 aa) [14,18]. In support of the hypothesis that proteins emerged first from random heteropeptides, it has been shown that modern proteins have a 90% chance of having a lengthwise distribution of amino acids that is indistinguishable from the random expectation [14]. Preliminary surveys concluded that the abundance of different amino acids in proteins is not dependent on the protein length or species of origin [23]. This suggest that small and large proteins should have indistinguishable amino acid composition. It also implies that bacterial and animal proteins (or other groups for that matter) should have the same amino acid bias.

In this work we wanted to revise many of these assumptions. We aimed to address the following questions: How does protein size vary in eukaryotic taxa? Which evolutionary forces influence protein number and protein size? Which theoretical function better describes the observed distributions of protein sizes? Is average protein size correlated to the total number of proteins or to the GC content of DNA? How well are small proteins annotated in the genomic releases? Is there an amino acids bias according to protein size? In order to answer those and other similar questions, we analyzed two independent sets of proteomic data.

## Results

### Construction and curation of protein datasets

We decided to compare the protein size distributions of different taxa in order to examine the factors that determine protein function, stability and evolutionary trends. In order to achieve this, we first had to construct and validate two datasets for this purpose (set 1 and 2). Selection of biological species, automatic filtering and manual curation of the protein data files was a necessary requirement to ensuring the reliability of the statistical analysis that was performed subsequently. Set 1 was biased towards eukaryotes and plant species, whereas set 2 was biased towards prokaryotic species.

**Figure 1:**
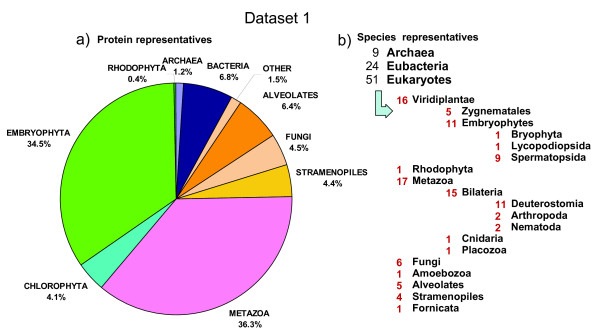
**Phylogenetic coverage of the protein dataset 1**. The protein dataset 1 was constructed from the proteomes from a wide range of Phylogenetic lineages downloaded from diverse sites on 2010 (Additional file 1: Table S1): Prokaryotes (100,681 proteins) and eukaryots (1,165,473 proteins). Around 34% belonged to plant species, 36% to animal species and the rest to other species. a) The pie reflects the percentage of protein entries that belong to a given phylogenetic lineage. b) Number of species within phylogenetic groups of the tree of life [24].

For dataset 1, the publicly available sequence genomic files were downloaded (see Additional file 1: Table S1) and duplicated protein sequences (identical amino acid sequence, or a sequence being an identical subsequence of other) were removed to yield a non redundant (nr) set (see Additional file 1: Table S2). For the prokaryotic group, we selected 9 archaeal species (15,089 nr proteins) and 24 representative bacterial species (85,592 nr proteins). From the eukaryotic group we selected 5 Alveolata species (81,215 nr proteins), one species of each of the following taxa: Amoenozoa, Fornicata, Placozoa (30,171 nr proteins), 6 fungal species (57,501 nr proteins), 4 Stramenopiles species (55,559 nr proteins) and 16 Eumetazoan species (447,717 nr proteins). From photosynthetic eukaryotic organisms, we selected one species of the following taxa Bryophyta, Lycopodiophyta, Rhodophyta (62,737 nr proteins), 5 Chlorophyta species (52,062 nr proteins), 5 dicot species (200,710 nr proteins) and 4 monocot species (177,801 nr proteins). In total, we obtained 1,266,154 nr proteins (see Additional file 1: Table S2) with a percentage coverage of each taxa as shown in Figure 1, which we considered -for our purposes- to be an acceptable snapshot of the genomic diversity that was available in the public domain at the time of downloading (May 2010).

**Figure 2:**
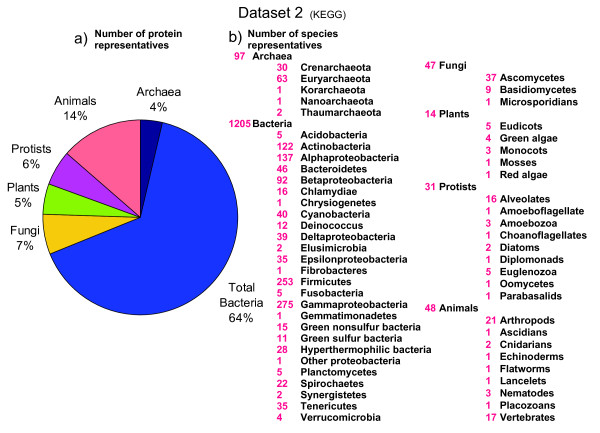
**Phylogenetic coverage of the protein dataset 2**. The protein dataset 2 was constructed from the full genomic sequences available in the KEGG database downloaded on the 27 of May 2011 (www.genome.jp/kegg/). Set 2 contained 6,169,140 proteins representing 1,442 species. a) The pie reflects the percentage of protein entries that belong to a given phylogenetic lineage. b) Number of species within phylogenetic groups of the tree of life [24].

In order to complement our study, we also downloaded all genomes from the KEGG database (downloaded on the 27 of May 2011; http://www.genome.jp/kegg/), which is biased towards prokaryotic genomes, but it also contains many eukaryotic species (see Additional file 1: Table S3). In the second set, we analyzed 1,442 species (97 archaeal, 1,205 bacterial and 140 eukaryotic species), representing 6,169,140 proteins (Figure 2).

### Average protein size in different species and lineages

**Figure 3:**
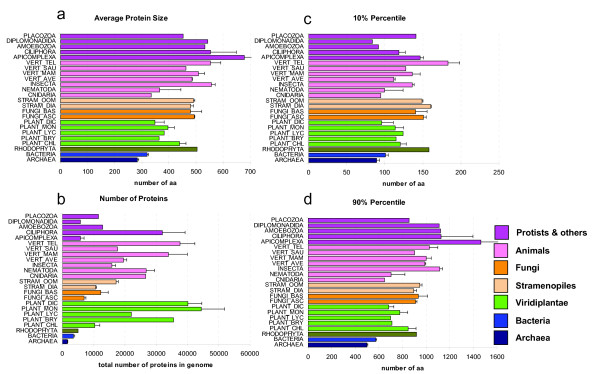
**Summary of protein number and protein size (set 1)**. Comparison of the protein length attributes in species from different phylogenetic groups. Species were grouped as indicated in Table 1. a) Average protein size. b) Total number of proteins in genome. c) Average of the 10% percentiles. d) Average of the 90% percentiles. Bars indicate mean values ± standard error (SE). In panels acd the x axis indicates the number of amino acids (aa), whereas in panel b it gives the average number of proteins in those species.

We first analyzed the distribution of protein sizes (sequence length in given number of amino acids (aa) per protein). Table 1 shows statistical parameters of protein size in each of the 84 species of dataset 1. Proteins were grouped according to phylogenetic groups and the mean values and standard errors were visualized as barplots (Figure 3). The average length of archaeal proteins (283 aa) was the smallest, followed by the average lengths of bacterial (320 aa) and eukaryotic (472 aa) proteins. Plant proteins (392 aa) were intermediate in size between bacterial (320 aa) and animal proteins (486 aa), whereas proteins from fungi (487 aa) and stramenopiles (486 aa) were as large as the ones from the eumetazoa (486 aa) (Figure 3). Similar average values were obtained from the analysis of the set 2 (data not shown). The differences between taxonomic groups for protein size were highly significant. The same conclusions were obtained when considering averages, medians or other percentile values for comparisons. This confirmed that the statistical analysis was sufficiently robust (given the great number of proteins analyzed) and not affected by the skewness of the distributions.

**Table 1:** Protein size summary.

			Length aa			Percentiles			
Group	Species Code	Species Name	Mean	SD	10%	25%	50%	75%	90%
ARCHAEA	ARC_PRO	*Archaeoglobus profundus DSM5631*	263	187	80	128	221	346	479
ARCHAEA	CAN_KOR	*Candidatus Korarchaeum cryptofilum OPF8*	296	191	104	160	262	379	501
ARCHAEA	CEN_SYM	*Cenarchaeum symbiosum A*	308	535	74	117	213	348	521
ARCHAEA	DES_KAM	*Desulfurococcus kamchatkensis 1221n*	272	188	75	129	238	369	499
ARCHAEA	MET_JAN	*Methanococcus jannaschii*	283	204	98	149	241	365	492
ARCHAEA	NAN_EQU	*Nanoarchaeum equitans Kin4-M*	276	203	91	142	225	352	512
ARCHAEA	SUL_ACI	*Sulfolobus acidocaldarius DSM 639*	284	183	96	146	249	375	511
ARCHAEA	THE_NEU	*Thermoproteus neutrophilus V24Sta*	268	182	91	142	230	346	463
ARCHAEA	THE_VOL	*Thermoplasma volcanium GSS1*	297	198	98	157	258	390	518
BACTERIA	ACI_FER	*Acidimicrobium ferrooxidans DSM 10331*	322	203	109	174	287	415	553
BACTERIA	BAC_FRA	*Bacteroides fragilis NCTC 9343*	361	249	107	182	310	455	691
BACTERIA	BAC_SUB	*Bacillus subtilis 168*	294	266	85	145	254	382	504
BACTERIA	BIF_ADO	*Bifidobacterium adolescentis ATCC 15703*	369	233	136	218	325	461	654
BACTERIA	BRA_JAP	*Bradyrhizobium japonicum USDA 110*	317	229	107	170	277	403	552
BACTERIA	BUR_CEP	*Burkholderia cepacia AMMD*	330	250	110	180	295	410	549
BACTERIA	CAM_JEJ	*Campylobacter jejuni RM1221*	294	202	83	150	254	392	538
BACTERIA	CHL_MUR	*Chlamydia muridarum Nigg*	355	296	105	172	290	446	650
BACTERIA	COR_AUR	*Corynebacterium aurimucosum ATCC 700975*	325	225	105	177	283	417	557
BACTERIA	DEI_DES	*Deinococcus deserti VCD115*	314	209	117	169	274	395	552
BACTERIA	ESC_COL	*Escherichia coli O157:H7 str. EC4115*	287	236	58	121	239	384	548
BACTERIA	GLO_VIO	*Gloeobacter violaceus PCC 7421*	313	233	95	151	256	398	593
BACTERIA	HYD_THE	*Hydrogenobacter thermophilus TK-6*	293	198	93	149	251	389	540
BACTERIA	KOC_RHI	*Kocuria rhizophila DC2201*	337	213	118	189	300	434	578
BACTERIA	LEP_BIF	*Leptospira biflexa Patoc 1 (Ames)*	338	216	123	184	292	430	611
BACTERIA	MYC_ABS	*Mycobacterium abscessus*	317	250	115	174	273	400	524
BACTERIA	PER_MAR	*Persephonella marina EX-H1*	304	240	95	152	256	392	569
BACTERIA	STA_AUR	*Staphylococcus aureus aureus MW2*	298	285	84	149	254	385	522
BACTERIA	STR_AVE	*Streptomyces avermitilis MA-4680*	341	308	115	182	289	422	578
BACTERIA	SUL_DEL	*Sulfurospirillum deleyianum DSM 6946*	312	223	101	166	266	403	577
BACTERIA	SYN_SP	*Synechocystis sp. PCC 6803*	319	256	96	153	264	404	584
BACTERIA	THE_ELO	*Thermosynechococcus elongatus BP-1*	314	214	98	157	273	403	577
BACTERIA	THE_THE	*Thermus thermophilus HB27*	303	199	109	167	264	390	529
BACTERIA	XAN_CAM	*Xanthomonas campestris pv armoraciae*	311	258	59	134	257	412	623
APICOMPLEXA	CRY_PAR	*Cryptosporidium parvum*	597	628	155	251	433	729	1192
APICOMPLEXA	PLA_FAL	*Plasmodium falciparum*	753	866	145	253	453	930	1707
APICOMPLEXA	TOX_GON	*Toxoplasma gondii*	682	766	139	224	441	843	1486
CILIOPHORA	PAR_TET	*Paramecium tetraurelia*	457	438	127	205	348	541	854
CILIOPHORA	TET_THE	*Tetrahymena thermophila*	649	660	110	229	456	839	1396
AMOEBOZOA	DIC_DIS	*Dictyostelium discoideum*	533	513	92	198	392	702	1123
DIPLOMONADIDA	GUI_LAM	*Giardia lamblia*	543	630	84	180	369	689	1110
PLACOZOA	TRI_ADH	*Trichoplax adhaerens*	453	426	141	217	345	539	854
FUNGI_ASC	PIC_STI	*Pichia stipitis*	492	346	161	263	416	613	893
FUNGI_ASC	SAC_CER	*Saccharomyces cerevisiae*	497	382	137	239	409	632	951
FUNGI_ASC	TRI_REE	*Trichoderma reesei*	491	452	154	262	408	600	891
FUNGI_BAS	LAC_BIC	*Laccaria bicolor*	370	312	88	153	289	488	749
FUNGI_BAS	PHA_CHR	*Phanerochaete chrysosporium strain RP78*	456	327	157	246	373	556	856
FUNGI_BAS	UST_MAY	*Ustilago maydis*	613	454	176	298	501	793	1198
STRAM_DIA	PHA_TRI	*Phaeodactylum tricornutum*	462	343	162	249	381	562	841
STRAM_DIA	THA_PSE	*Thalassiosira pseudonana*	499	424	159	249	391	613	947
STRAM_OOM	PHY_RAM	*Phytophthora ramorum*	479	407	152	237	373	584	903
STRAM_OOM	PHY_SOJ	*Phytophthora sojae*	502	447	146	234	382	616	986
CNIDARIA	NEM_VEC	*Nematostella vectensis*	335	336	95	145	250	405	646
INSECTA	ANO_GAM	*Anopheles gambiae*	529	547	132	223	389	632	1065
INSECTA	DRO_MEL	*Drosophila melanogaster*	584	642	141	242	427	700	1164
NEMATODA	CAE_ELE	*Caenorhabditis elegans*	444	484	124	211	342	522	820
NEMATODA	PRI_PAC	*Pristionchus pacificus*	288	285	76	116	206	359	583
VERT_AVE	GAL_GAL	*Gallus gallus*	490	508	108	184	346	608	1007
VERT_AVE	MEL_GAL	*Meleagris gallopavo*	479	463	116	197	351	595	968
VERT_MAM	BOS_TAU	*Bos taurus*	495	490	145	246	356	592	947
VERT_MAM	EQU_CAB	*Equus caballus*	564	606	147	247	393	688	1139
VERT_MAM	HOM_SAP	*Homo sapiens*	456	540	98	163	311	562	947
VERT_MAM	MON_DOM	*Monodelphis domestica*	574	489	174	295	457	719	1069
VERT_MAM	ORN_ANA	*Ornithorhynchus anatinus*	445	416	123	202	327	540	868
VERT_MAM	RAT_NOR	*Rattus norvegicus*	520	500	130	224	374	643	1039
VERT_SAU	ANO_CAR	*Anolis carolinensis*	462	436	128	207	346	559	903
VERT_TEL	DAN_RER	*Danio rerio*	473	456	151	234	363	565	879
VERT_TEL	TAK_RUB	*Takifugu rubripes*	634	536	215	324	494	780	1177
PLANT_BRY	PHY_PAT	*Physcomitrella patens*	363	308	115	165	278	461	711
PLANT_CHL	CHL_REI	*Chlamydomonas reinhardtii*	503	589	97	173	335	608	1074
PLANT_CHL	MIC_CCM	*Micromonas CCMP1545*	426	390	123	202	334	522	799
PLANT_CHL	MIC_RCC	*Micromonas RCC299*	485	475	146	236	371	571	920
PLANT_CHL	OST_LUC	*Ostreococcus lucimarinus*	397	343	121	199	319	486	726
PLANT_CHL	OST_TAU	*Ostreococcus tauri*	387	349	114	186	307	476	716
PLANT_DIC	ARA_THA	*Arabidopsis thaliana*	403	299	115	202	345	513	749
PLANT_DIC	CAR_PAP	*Carica papaya*	296	249	68	112	225	411	611
PLANT_DIC	GLY_MAX	*Glycine max*	422	354	139	220	353	529	768
PLANT_DIC	MED_TRU	*Medicagao truncatula*	245	245	59	78	149	334	550
PLANT_DIC	POP_TRI	*Populus trichocarpa*	375	292	101	167	306	490	732
PLANT_LYC	SEL_MOE	*Selaginella moellendorfii*	382	300	124	191	316	481	699
PLANT_MON	BRA_DIS	*Brachypodium distachyon*	428	303	146	223	361	537	788
PLANT_MON	ORY_SAT	*Oryza sativa ssp. japonica*	448	389	108	174	332	574	960
PLANT_MON	SOR_BIC	*Sorghum bicolor*	361	282	103	167	288	476	706
PLANT_MON	ZEA_MAY	*Zea mays*	345	258	97	164	286	455	655
RHODOPHYTA	CYA_MER	*Cyanidioschyzon merolae*	504	404	158	259	412	628	918

Statistical summary of protein length values in the proteomes of dataset 1 including 84 different species (9 archeal, 24 bacterial and 51 eukaryotic organisms). The mean, standard deviation (SD) and the 10%, 25%, 50%, 75% and 90% percentiles were calculated for each organism individually (see methods)

### Protein size of functional KO categories across taxonomic groups

Three strategies were followed in order to confirm that average protein size was not derived from genomic artefacts and data outliers generated by transposons, alternative spliced proteins and gene family duplications: 1) we performed mathematical modeling for decreasing the influence of outliers (see later sections), 2) we filtered transposon proteins and made a separate analysis of size distribution, and 3) we grouped genes according to functional categories and compared average sizes of different taxonomic groups (see below).

For strategy 2 we selected the best annotated plant genome (*Arabidopsis thaliana*) as representative eukaryotic species. We filtered all proteins that contained particular keywords in the gene annotation (e.g. transposon, transposase, retrovirus, etc.) and made a separate analysis of protein size (data not shown). The results confirmed that transposon related proteins did neither affect the distribution models nor any of the other results (averages, medians and percentiles shown in Table 1).

**Figure 4:**
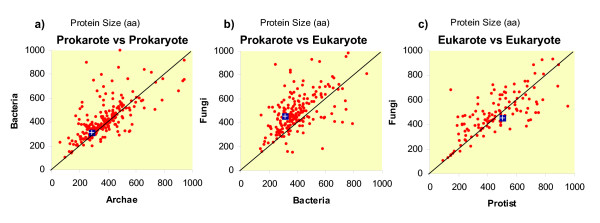
**Comparison of functional category average protein size between different taxa (dataset 2)**. The average size of proteins of KEGG ontology (KO) categories were calculated for each taxonomic group and then plotted to each other. Each red point represents a KO category. The blue rectangle indicates the average size of all proteins of those taxa. The diagonal black line indicates identical size in given number of amino acids. Dots above the diagonal indicate proteins that are larger in y-axis species as compared to the x-axis species.

For strategy 3, the KEGG ontological categories (KO) were assigned to proteins of dataset 2. The average protein sizes of each KO category were plotted for comparing taxa (Figure 4). The size differences between archaeal and bacterial proteins were distributed among many but not all KO categories (Figure 4a). This means that some KO categories of proteins were larger in bacterial species, but other KO categories were smaller than archaeal proteins. On a global average, archaeal proteins were significantly smaller than bacterial proteins, as previously shown in Table 2. This means that one of the selective forces that shaped size differences between prokaryotic taxa increased the number of proteins of some KO categories (e.g. categories with large proteins). Thus, among prokaryotes, protein size (within the same KO category) did not increase so much through domain addition or gene fusion (Table 1). The 90% percentile of plant proteins is in the range of 649-877 aa, whereas in animals it is in the range of 909-1,125 aa (Table 1).

In contrast, when comparing bacteria and fungi (as eukaryotic representative) the size differences were present in many KO categories (Figure 4b). Proteins of most KO categories were larger in size in fungi than in bacteria (Figure 4b). This can be interpreted that the average differences of protein size (Table 2) was not an artifact, but it has been caused by a mechanism of gene extension, through domain addition or gene fusion.

Finally, eukaryotic taxa were also compared to each other. Proteins belonging to the same KO category were of variable size in different taxonomic groups, e.g. as shown between fungi and protist (Figure 4c). This means that the observed size differences between eukaryotic groups (see Table 1 and Figure 3) were caused by both evolutionary mechanisms, by gene duplication (increasing the number of large proteins) and by gene fusion (altering average protein size of some selected KO categories).

### Protein number and protein average size in different species

**Figure 5:**
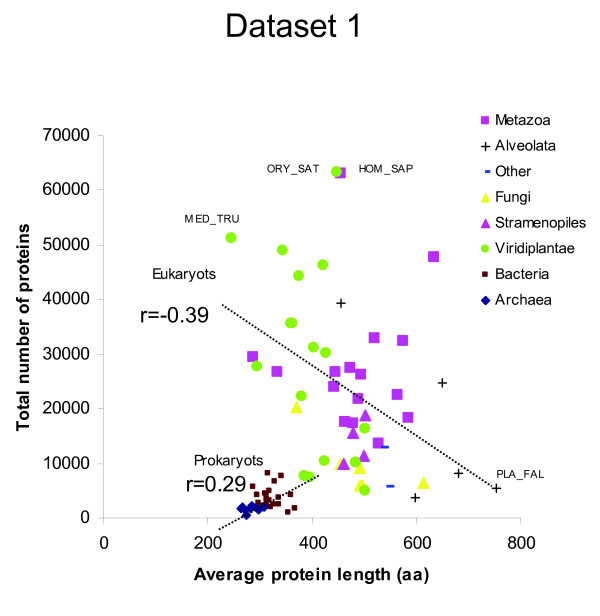
**Relationship between the total number of proteins in a genome and the average protein length (dataset 1)**. The average protein size (number of amino acids) was plotted against the total number of proteins in the genome of 9 archaeal, 24 bacterial and 51 eukaryotic species.

**Figure 6:**
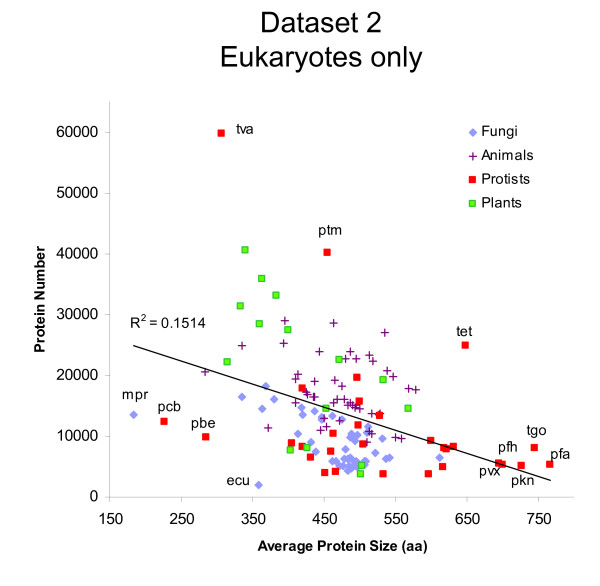
**Relationship between the total number of proteins and average protein length in eukaryotes (dataset 2)**. The average protein size (number of amino acids) was plotted against the total number of proteins in the genome of 140 eukaryotic species. KEGG three letter code of highlighted species: *Moniliophthora perniciosa *(mpr), *Plasmodium chabaudi *(pcb), *Plasmodium berghei *(pbe), *Encephalitozoon cuniculi *(ecu), *Trichomonas vaginalis *(tva), *Paramecium tetraurelia *(ptm), *Tetrahymena thermophila *(tet), *Plasmodium vivax *(pvx), *Plasmodium falciparum HB3 *(pfh), *Toxoplasma gondii *(tgo), *Plasmodium knowlesi *(pkn), *Plasmodium falciparum 3D7 *(pfa).

After detecting significant differences in protein length among lineages (Table 1), we studied the relationship between the average protein length and the total number of proteins coded in the genomes. In dataset 1, a low positive correlation between the total number of proteins and the average protein length of each species was found (Figure 4; *r *= 0.25, n = 84, *p *= 0.024). However, this relationship arouse from the strong difference between prokaryotic and eukaryotic species. When species of data set 1 were analyzed as two separate kingdoms, the number of proteins per genome correlated positively for prokaryotes (*r *= 0.25) but negatively for eukaryotes (*r *= -0.39) (Figure 5). A similar negative correlation value was found for eukaryotic species of dataset 2 (*r *= -0.39, n = 140, *p *= 2 × 10^-6^) (Figure 6).

### Range of average protein size differences

Plotting average protein sizes showed that the values were quite narrowly distributed for prokaryotic species, but it had a much larger spread in eukaryotes (Figure 4). Protein size of archaeal species were even more narrowly distributed than bacterial proteins (Figure 4). Among eukaryotic species, the spread was larger for protists than for animals or fungi (Figure 6). Protist species have longest or shortest protein sizes, or also the genome with the most numerous genes (Figure 6). This indicates that there is larger diversity of protein number and size among unicellular protists species than among all other taxa. This parallels 16SrRNA, where protists showed the largest amount of diversity among eukaryotes [25].

### GC content of coding DNA and average protein size in different species

We also studied the possible relationship between protein number, size and GC content of the genomes. We therefore measured the gene-based GC content for all species in dataset 2 (see methods). The overall correlation coefficient between average protein size and GC content was barely significant (r = 0.05, *n *= 1,442; *p *= 0.048). When analyzed as separate kingdoms, correlation values were non-significant in Eukaryotes (r = -0.05, *n *= 140; *p *= 0.53) and Archaea (r = 0.021; *n *= 101; *p *= 0.84) but significant in Bacteria (r = 0.21, *n *= 1,239; *p *= 9.7 × 10^-14^).

Correlation values between protein number and GC content were: All species (r = 0.17, *n *= 1,442; *p *= 3.1 × 10^-11^), Eukaryotes (r = -0.05, *n *= 140; *p *= 0.53), Archaea (r = 0.44; *n *= 101; p = 2.7 × 10^-6^) and Bacteria (r = 0.58, *n *= 1,239; *p *= 8.6 × 10^-114^).

These results are in accordance with the hypothesis that protein size in eukaryotic organisms (as compared to prokaryotes) has been under distinct selective pressures during the evolution of lineages [26]. The positive correlation found for bacterial species gives support for the theoretical prediction that the length of ORFs increases with the GC content of DNA due to the AT bias of stop codons [7]. It is interesting to note that this correlation applied only for bacterial genomes but not for complex organisms with large genomes. However, other potential explanations, like selection for smaller genes to increase the rate of duplication in genomes under a reductive process cannot be ruled out.

### Size anomalies in the protein length histograms of different species

**Figure 7:**
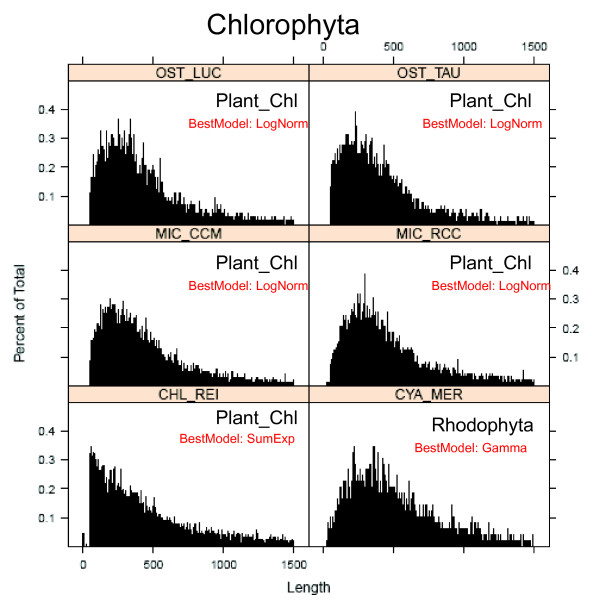
**Protein size histograms in algae (Chlorophyta)**. Emprirical distribution of protein length in some representative algal genomes. The range of protein lengths in the x-axis is from 0 to 1,500 aa. The y-axis indicates the percentage of proteins that fall into the given interval bins of 1 aa. The letters in red indicate the theoretical model that best fits the observed distribution. Consult Table 1 for species abbreviations.

**Figure 8:**
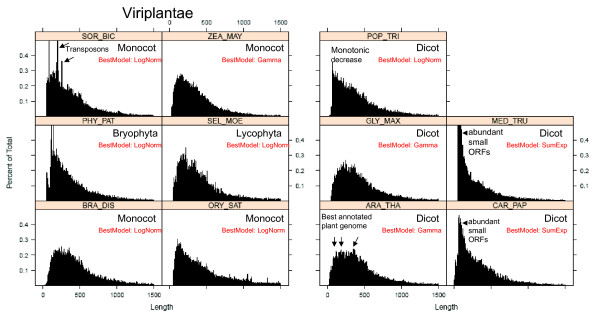
**Protein size histograms in dicot and monocot plants (Viridiplantae)**. Empirical distribution of protein length in some representative plant genomes. The range of protein lengths in the x-axis is from 0 to 1,500 aa. The y-axis indicates the percentage of proteins that fall into the given interval bins of 1 aa. The letters in red indicate the theoretical model that best fits the observed distribution. Consult Additional file 1: Table S1 for species abbreviations.

**Figure 9:**
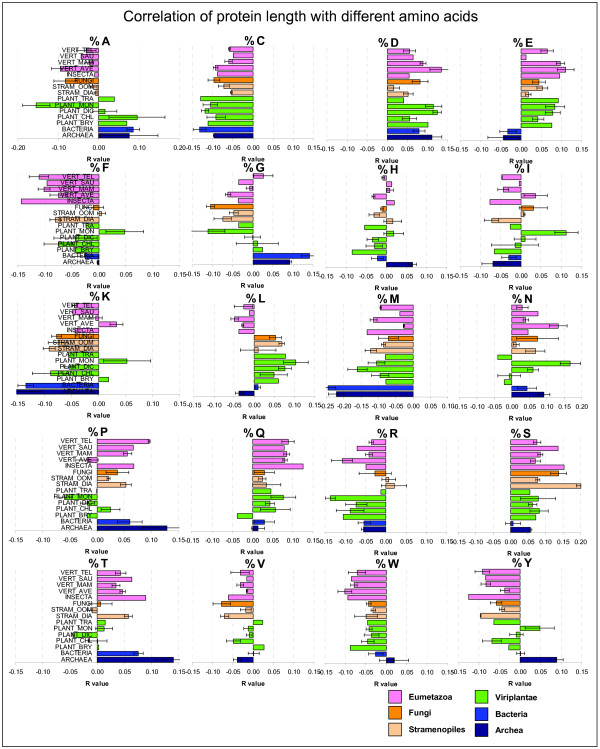
**Protein length amino acid bias**. Correlation of percentage amino acid composition with protein length were calculated for each species individually (see Table S4). The pearson correlation coefficients *r *were then averaged according to taxonomic groups. Bars indicate mean values and standard errors across species of the same taxa. Positive or negative *r *values indicate the direction of the correlation (e.g. the amino acid C is negatively related to protein length, thus small proteins have more %C than large proteins).

When protein length distributions in different species were compared in more detail, many common features were found (e.g. gamma type distributions), but also some striking differences and anomalies (Figure 7, 8). For example, some proteomes contain several local maxima, i.e. peaks in the range 200-400 aa (Figure 6, 7, 8, 9). In Arabidopsis thaliana, there was a first peak at 176 aa, a secondary peak at 238 aa, and a third peak at 363 aa (Figure 8). In Sorghum bicolor there were several prominent peaks of sizes 51 aa, 83 aa, 191 aa, 196 aa and 254 aa (Figure 8). The presence of several local maxima in some species could suggest that strong selective forces are able to increase the number of proteins of given sizes above the frequency predicted by the theoretical models. More functional details of those protein peaks will be presented in a follow-up study.

### Theoretical fit of protein length distributions

Finding the best fit of protein length distributions to well characterized mathematical models can offer insights about evolutionary trends, selective pressures and constraints for protein function and structure [27]. Since protein stability is determined in part by length [28,29], the size of proteins has a selective advantage and therefore, influences the evolution of proteomes in each lineage. One could assume that proteins smaller than 130 aa are less functional or less stable than proteins larger than 200 aa. Indeed, the need to explain the origin of large proteins was one of the major reasons for invoking gene or exon duplication in the starter-set hypothesis [10,30]. If size distributions can be explained by a simple stochastic model without assuming massive gene or exon duplication; then, the random-origin hypothesis would be supported. If the same model applies for all species, one could also hypothesize that protein size has increased by the same evolutionary process that caused bacterial proteins to be larger than archaeal proteins.

Therefore it is important to find the model that most accurately describes the protein distribution in each of the selected species. We compared the following theoretical distributions: 1) gamma with fixed shape parameter 2) gamma distributions with no restriction of the shape parameter, 3) the log-normal distribution, and 4) a distribution resulting from the sum of two exponential random variables. For each theoretical function we estimated all the parameters (see methods), and we obtained the Akaike's Information Criterion (AIC) for each fit for each species of set 1 (Additional file 1: Table S3) and set 2 (data not shown). For the species shown in Figure 7, 8 the model that had the lowest (most negative) AIC value is shown in small red letters. According to the AIC criterion the log-normal model best fits the data for 40 out of 84 species of set 1; the gamma model best fits the data for further 37 species and the sum of exponential model best fits the remaining 7 species (Additional file 1: Table S3). In set 2, the best fit models were: log-normal (601 species), gamma free (699 species), gamma with fixed shape parameter (71 species) and sum of exponential (71 species) (data not shown).

**Figure 10:**
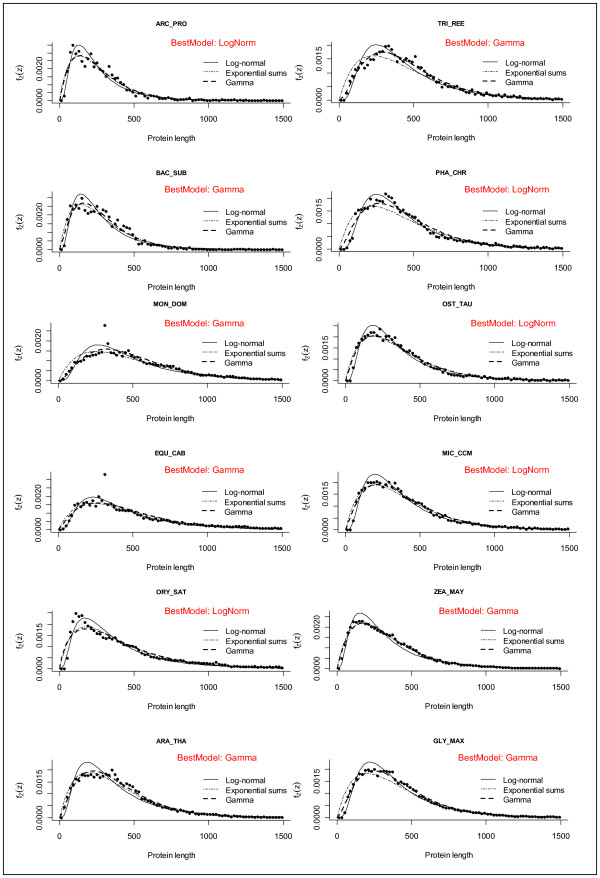
**Fitting to theoretical distributions**. Protein length distributions for 12 typical genomes: Archea (*Archaeoglobus profundus *ARC_PRO), Bacteria (*Bacillus subtilis *BAC_SUB), Eumetozoa (*Equus caballus *EQU_CAB, *Monodelphis domestica *MON_DOM), Fungi (*Trichoderma reesei *TRI_REE, *Phanerochaete chrysosporium *PHA_CHR) and Viridiplantae (*Ostreococcus tauri *OST_TAU, *Micromonas CCMP1545 *MIC_CCM, *Oryza sativa *ORY_SAT, *Arabidopsis thaliana *ARA_THA, *Zea mays *ZEA_MAY, *Glycine max *GLY_MAX). The continuous line represents the log-normal fitted model (the corresponding estimated parameters appear in Additional file 1: Table S3) the dots represents the relative frequency counts for the observed data in bins of 10 aa.

In order to visualize the goodness of the theoretical fits, we plotted the models on top of the real data for a few selected species (Figure 10). In those figures one can easily observe that there are datasets for which the log-normal model fits very well but there are cases that other functions fit better (Figure 10). It can also be seen that although the models explain the sizes very roughly, there are many datapoints that lie outside the fitted models (Figure 10).

### The shape parameter of the gamma function

Previous attempts to model protein size distributions had used a gamma distribution with fixed shape parameter equal to 2 [14,18]. To examine the soundness of this assumption, we modeled gamma distributions without the fixed shape parameter restriction, and then analyzed the estimated shape parameter in all species of dataset 1 (Additional file 1: Table S3) and dataset 2 (data not shown). The empirical distribution of the shape parameter for the gamma models had a mean of 2.3 in both data sets (Additional file 1: Figure S1). Using hypothesis testing from normal theory, we determined that the statistical feasibly of a fixed value of 2 is negligible (*p *= 7.11 × 10^-9^). This means that gamma models with fixed shape parameter equal to 2 are inadequate for describing protein size distributions. Thus, the shape parameter is not strictly 2 as earlier assumed by [18], but it can vary between the extreme values of 1 and 3 depending on the species (Additional file 1: Table S3).

### Modeling of size distribution can detect data outliers

In order to visualize the goodness of the theoretical fits, we plotted the models on top of the real data for a few selected species (Figure 9). In those figures one can observe datasets for which the log-normal model fits well but there are also cases for which other functions (e.g. the sum of exponential distribution) fit better (Figure 10). Although the models explain the sizes roughly, there are some extreme data points that lie outside the fitted curves (see size ~330 aa in Figure 9 MON_DOM and EQU_CAB).

The fitted values of the models can be used to estimate theoretical protein sizes based on the idealized functions. For example, the theoretical median size can be estimated with the meanlog value (μ) of the log-normal function, whereas the expected value can be calculated with the meanlog (μ) and sdlog (σ) (see methods). The same general conclusions were obtained when comparing the theoretical average and median values from the fitted models (data not shown) with the previous conclusions drawn from the arithmetic values (see Table 1 and Figure 3). Thus, the main conclusions from this study are robust and statistically reliable and not an artifact of extreme outliers or single size anomalies.

### Modeling of size distribution can detect genomic annotation artifacts

Comparison of the fitted models between different species also allowed us to detect genomic releases that had an aberrant distribution of protein sizes with very abundant occurrence of small ORFs (see Figure 8 MED_TRU, CAR_PAP and POP_TRI). In those genomes, small proteins of size < 100 aa are more abundant then proteins of size 200 aa. As discussed below, mathematical modeling could be used as a tool for discriminating genomic releases that have a better annotation of small ORFs (see discussion).

### Amino acid bias depending on protein length

In addition to studying the length of proteins, we also investigated the amino acid (aa) composition in both absolute terms (occurrence) and relative amounts (percentage occurrence). We asked whether protein length has any amino acid bias (e.g. if small/large proteins have more/less of any amino acid). Do small proteins contain more cysteines for stabilizing their structure? Do proteins from plant species have different properties from proteins of other phylogenetic groups? In order to answer these questions, we first calculated percentage aa composition of each protein of a species, we then calculated the Pearson correlation coefficient *r *of the 20 aa to protein length (Figure 9). The obtained r values (negative or positive) were then averaged across phylogenetic groups and plotted horizontally with error bars (Figure 9). There were marked differences between prokaryotic and eukaryotic species. For example, glycine was positively correlated to protein length in prokaryotes but not in eukaryotic proteomes (Figure 9g), whereas the opposite was true for glumatic acid (Figure 9e). The r value was also different for other amino acids (Figure 9). Threonine had a high positive r value in archaea and bacteria, much less in animal species and almost a zero or negative value in plant species (Figure 9). Negative r values were found for cysteine, methionine, lysine, fenilalanine, tryptophan and tyrosine, whereas positive r values were found for aspartate, glutamate, serine (Figure 9). Positive r values means either that longer proteins tend to have more from that particular amino acid or that short proteins tend to have less from that amino acid. The opposite is true for negative r values. For example, it seems that long proteins have less cysteine as expected, either because it is detrimental for long proteins, or because cysteine helps to stabilize short proteins. The lowest r values were found for histidine and valine (Figure 9), meaning that those amino acids have rather a neutral effect on protein length.

## Discussion

The overall goal of the study was to investigate how similar or dissimilar are protein sequences across different taxa of eukaryotic organisms. In this paper we focused on average protein length, protein number and amino acid compositional bias. We also tested the fit of the length distributions to different theoretical models. We determined the mathematical function that best fits the empirical distribution of protein size in each organism.

Protein length distribution has not been previously understood as a selective trait *per se *(i.e. it is not a character that is directly selected for in classical evolutionary terms [31]). Individuals are selected for having inheritable units (e.g. genes or epigenetic states) whose products (e.g. proteins) confer a selective advantage to their carriers and progeny. Protein features under direct selection could include many, like the specificity and efficiency of a reaction when the protein is an enzyme, or the thermo-stability of the protein, among others [31,32]. Physicochemical restrictions must also play a role, for example, very small proteins might not fold properly, and the chances to evolve useful proteins are reduced for extreme sizes. Protein size directly affects the number of functions accessible to a polypeptide, and it is also indirectly associated to many features that are indeed under direct selection [31]. The actual shape of the protein length distribution in a particular genome has to be an interplay between mutation, recombination, fusion, fission, deletion, selection, physicochemical restriction and history. The challenge then becomes to explain how these factors have contributed each, to originate a particular distribution.

### There are significant differences of average protein size in different eukaryotic species

Previous studies on the statistical distributions of the lengths of modern protein sequences have focused on prokaryotic species. It was already known that archaeal proteins are on average smaller than bacterial proteins [4,5]. Some preliminary surveys also concluded that proteins from eukaryotic species are larger than bacterial proteins. Brocchieri and Karlin (2005) analysed five eukaryotic species [5], whereas Zhang (2000) studied only two eukaryotic species (*Saccharomyces cerevisiae *and *Caenorhabditis elegans*) [4]. Since the analysis of few species can lead to severe statistical bias due to limited sampling, we considered necessary to analyze protein length in a much larger and diverse set of eukaryotic species. We constructed large protein datasets of prokaryotic (set 1 n = 33, set 2 n = 1,302) and eukaryotic species (set 1 n = 51, set 2 n = 140), including fungal, animal and plant proteomes (Figure 1, 2). We then estimated size differences among all species (Table 1) and confirmed previous reports [4] (see above) that eukaryotic proteins are larger on average than bacterial and archaeal proteins (Figure 3). We show that average protein size could be due to an altered number of proteins or to an altered size of proteins within a functional KO category (Figure 4).

Furthermore, the large variability of eukaryotic protein length followed some phylogenetic relationships (Figure 3). Plant species had particularly small proteins among all eukaryotes (Figure 3). Simple animal organisms like Nematostella vectensis and Pristionchus pacificus had protein sizes that were similar to plant species (Table 1). We also found that unicellular eukaryotic organisms tend to have larger average sizes than multicelular species (Table 1). For example, the apicomplexa group has larger proteins than the group of vertebrates, whereas chlorophyta group has less but larger proteins than the other groups of plants (Figure 3). In order to confirm these observations we compared all unicellular eukaryotes against all multicellular species of dataset 2 and found that the abovementioned differences were significant for protein number (*p *= 9.6 × 10^-12^), GC content (*p *= 0.0011) and protein size (*p *= 0.0018).

### There are marked peaks but no marked gaps in the protein length distribution curves

After observing the strong size differences among phylogenetic groups we analyzed the distribution curves to see how smooth and homogenous are proteins distributed in size (Figure 7, 8). We detected protein bins of 1 amino acid (aa) that were more frequent than expected by the theoretical models (Figure 9). Gaps in the distribution curves would have indicated that there are protein sizes that are prohibited by structural or functional reasons. For example stably functional proteins can be formed by the very common structure of an eight-stranded α/β barrel (TIM barrel) [33-35], but there are no reported functional proteins that have five or nine α/β strands [36,37], and thus one could expect gaps at given size intervals. Since ~200 aa residues are required to fully form a TIM barrel (each α/β strand consists of ~25 aa residues) [2], one could expect that some protein sizes are less frequent than those which have a multiple of 25 aa or 200 aa. However, no marked gaps or such regularities were observed in the histograms (Figure 7, 8). This indicates that there are no prohibitive structural constrains of protein size along the whole range of observed sizes. Instead, marked peaks were indeed observed, which corresponded to data outliers in the fitted models (Figure 9). This can be explained by massive duplication of particular genes. Most noticeably, all mammal species had a very prominent single peak of size ~332 aa in the distribution curves (Figure 9 MON_DOM and EQU_CAB). In plants, some prominent peaks were due to multiple copies of transposon-encoded proteins like the 191 aa peak in Sorghum bicolor (Figure 8).

The significant deviations from the idealized functions, and the strong differences of the distributions among different organisms, indicates that protein length distributions are strongly influenced by specific selective pressures. One of the evolutionary mechanisms is gene duplication and subfunctionalization leading to large gene families. An example for selective pressure for gene duplication is the need for a large repertoire of olfactory receptors in mammal species [32], leading to large increase of proteins with length = 332 aa (Figure 9).

### The theoretical models that best describe the distribution of protein length are the log-normal function and the gamma function with unrestricted shape parameter

Finding the best fit of distributions to known mathematical models can offer some useful biological insights. Protein length analyses of modern species not only could provide clues to better explain the origin of primitive proteins [14], but it can also provide valuable information on selective pressures that have prevailed during evolution. A good fit to a gamma function had been previously used to sustain the hypothesis that proteins evolved from random nucleotide sequences [18]. The gamma function with shape parameter 2 describes a probability density function that results from the combined action of two independent random variables exponentially distributed with parameters α > 0 and β > 0 respectively. A simple theory for this theoretical distribution is that the occurrence of stop codons in a random nucleotide sequence leads to exponentially distributed protein lengths, whereas selective pressure for protein stability, folding capacity, and potential biochemical activity is dependent on sequence length, so that small proteins (< 100 aa) have a limited potential for a useful biological function, and thus are rather discarded or negatively selected for [18].

However, the assumption of a fixed shape parameter 2 had to be rejected on statistical grounds (Additional file 1: Figure S1). The lognormal function had a better fit in 48% of the species, making it almost equivalent to the gamma function with free shape parameter. Furthermore, the sum of exponential functions had a better fit in only 8% of the species, particularly of recently sequenced genomes with not so long history of curation and manual annotation. It can be concluded from all previous results that the theoretical model that better describe protein size distribution is the gamma function with unrestricted shape parameter.

### Why do genomes have a protein size distribution different from the theoretically expected?

The genetic code allows making some theoretical predictions about average protein size and frequency distribution [7,8,38]. Since stop codons can appear stochastically after any start codon, then larger proteins should always be less frequent than smaller proteins. The most frequent protein sizes should be 1 aa in length [7]. However, distributions of well annotated genomes such as *Arabidopsis thaliana *do not decrease monotonically but rather increase sharply at about ~80 aa, peak several times in the range of 150-250 aa and then decreases gradually (Figure 7, 8). In most genomes, proteins of size 151-250 aa were more frequent than proteins of size 51-150 aa and even more than proteins of size 1-100 aa (Figure 7, 8). One can interpret this as evidence of a selective pressure for the avoidance of proteins smaller than 100 aa and the selective advantage of functional proteins of > 250 aa. The characteristic increase of proteins in the range 50-200 aa can be explained with the abovementioned selection force, whereas the monotonic decrease of frequency in the range 500-1,000 aa can be explained by the probable occurrence of stop codons in the coding determining sequence (CDS).

If one considers simple models, the average protein size should be ~21 aa [7]. If one considers more sophisticated models explaining the length of random open reading frames (ORFs) in the intergenic regions of yeast [8], random ORFs of ~33 aa can be explained by the mummy and baby ORF theory alone [8,39]. However, the average eukaryotic protein is much larger than 100 aa (Table 1). We assume that the frequent occurrence proteins of size 150-250 aa is due to protein folding stability (for example TIM barrels) that generates a selective pressures avoiding stop codons within exons or genes. We postulate that this force is so strong in eukaryotic species, that it overcomes the influence of the GC content of DNA on average ORF length as indeed found in prokaryotic genomes.

Since most prokaryotes (archaea and bacteria) lack introns, the fact that eukaryotic proteins are much larger can be explained because proteins usually are encoded in multiple exons [40]. In follow up studies we will analyze how the statistical frequency of stop codons limits the maximal protein length in prokaryotic species that do not have splicing mechanisms.

### Limitations for the occurrence of small proteins

Why are proteins of size 150-250 aa so frequent? Why are proteins smaller than 150 aa so infrequent in some genomes but not in others? Is it the result of bioinformatic annotation procedures? In the yeast genome, many ORFs < 100 aa are likely non-coding or over annotated [41]. Some genomic annotating algorithms are instructed to ignore small open reading frames with a minimal cutoff of ~33 aa in order to limit the number of false positive ORFs [42]. Some proteomes show clearly such drastic cutoffs in the range of 20-60 aa (Figure 7, 8). Small proteins could have important biological functions, however there is a statistical justification in that smaller proteins are more difficult to predict than larger ones [42]. Therefore, the definition of cut off limits the number of false ORF predictions. A cutoff increase from 21 aa to 33 aa might be supported by the mummy and baby ORF theory [8]. Sophisticated measures of nucleotide bias at the DNA level (asymmetry in the composition of the first and the second positions in the codons) can help to detect spurious non-coding ORFs in the yeast genome [41]. However, we propose using additional bioinformatic tools at the protein level, such as mathematical modeling and amino acid bias to exclude false positive small ORFs rather than defining an arbitrary cutoff of protein size. For example, from two equally small proteins, it is more likely to be functional the one that contains more %C, %M, %K, %F, %R, %W and %Y and less %D, %E, %Q, %S and %T (Figure 9). The importance of amino acids like C is not surprising, since cisteine bridges stabilize the structure of small proteins.

### Biological functionality of small proteins

As previously mentioned, the protein length distribution of most eukaryotic organisms is not monotonically decreasing, but increases from 1-200 aa (see Figure 7, 8). What determines the minimal size of a biologically active protein? Is it the function, the structure or the capacity to be regulated? Finding a biochemical explanation for a threshold of small proteins is neither simple nor trivial. An active site of an enzyme typically consists of only 3-5 amino acid residues correctly accommodated in space [1]. If enzymatic catalysis can be carried out by only so few amino acids, then the remaining residues (> 97%) of eukaryotic proteins are maybe only for accommodating those catalytic residues correctly in space and for spatial filling. The smallest known enzymes are about 10 kDa (~100 aa) in size with some extreme cases like an enzyme of 62 aa that forms a stable homopentamer (5 × 62 aa = 310 aa) [43]. Thus it seems that even the smallest enzymes achieve stable folding and regulatory features only when more than 200 aa residues interact spatially. Proteomic surveys show that the most abundant proteins in SDS-PAGE gels of plant extracts are in the range of 20-60 kDa (~200-600 aa) with very few proteins appearing in the range below 10 kDa (data not shown). Most enzymes form quaternary structures of several polypeptides, for example the often found di-mers or tetra-mers (http://www.expasy.org/).

From the bioinformatic survey of eukaryotic organisms (n = 140) we conclude that the range of 150-250 aa is the optimal length for a biologically active polypetide. Does that represent a waste of resources? Probably not, since a minimal size of > 200 aa might be required for conferring regulatory properties to enzymes and proteins. More work and deeper studies are needed to address such open questions on enzymatic function and biological capabilities of small proteins.

### What limits the occurrence large proteins in plants?

We found that there are not so many long proteins in plants (Figure 3). But, why? We found that plant proteins are on average encoded by less exons than in animal genomes (data not shown). What limits a more frequent appearance of multi-exon genes in plants in comparison to animals? Is there any metabolic efficiency and amino acid composition in plant proteomes? Biosynthetic cost-minimization of bacterial proteins has been postulated as an explanatory hypothesis for differences in evolutionary fitness [44]. In bacteria, it has been shown that the energetic advantage of using different amino acids for highly expressed genes can be a substantial proportion of the total energy budget [45]. Is there cost-minimization of amino acid usage in plant proteins? Or is the reason the appearance of more complex proteins in animals. Plant genomes have numerous genes, but it seems that the average plant protein is not only smaller but it is also encoded by less exons, thus suggesting that sequence length differences could reflect a difference in protein multi-functionalities between plant and animal proteins. More work is required for the analysis and comparison of the multiplicity of PFAM and Interpro domains in plant and animal proteins in order to statistically test such hypotheses.

### Is there an universal evolutionary trend towards larger proteins?

The average length of polypeptides in archaea (~283 aa), bacteria (~319 aa) and eukaryot (~472 aa) are significantly different (Table 1). The progressive increase of protein size among archaea, bacteria and eukaryotes has been interpreted as a constant evolutionary trend for larger proteins [4]. Did eukaryotic proteins become steadily larger through domain fusion as suggested by Brocchieri & Karlin (2005) [5] The negative correlation between protein number and average protein size among eukaryotes (Figure 4, 5, 6) provides support for the hypothesis that proteins can increase or decrease their average size through the fusion or splitting of protein domains. However, we rule out the possibility of a steady trend for size increase in all organisms. Some phylogenetic groups had larger proteins while others had smaller proteins, and this is not related to the evolutionary time of emergence of those eukaryotic lineages. Most noticeably was the extreme variability of protein size and protein number in protist species (Figure 6). This indicates that protein size can depend greatly on short-term environmental adaptations.

Among eukaryotes, there was an indirect relationship between total protein number and average protein size (Figure 5, 6). It is therefore tempting to speculate that proteins can fuse together, so that many small proteins can become fewer larger proteins. This could be because some organisms have better a adaptation when they have less but more multifunctional proteins (larger size), while other species are better adapted to specific environments, when they have more but less multifunctional proteins (smaller size).

In a nutshell, compared to prokaryotic species, eukaryotic proteomes have been shaped by distinct evolutionary forces that have favored massive gene duplication events (increase of protein number) and domain addition (increase of average protein size).

### The shape of histograms and the fit to theoretical distributions could be indicative of the efficiency of the bioinformatic procedures for annotating small proteins

As shown in Figure 7, 8, the empirical distribution of protein lengths for many species show the characteristic shape of a log-normal or a gamma function. However, there are some exceptions. For example, the protein size distribution in *Chlamydomonas reinhardtii *(CHL_REI) is monotonically decreasing from 50 aa to 250 aa (best fit to sum of exponentials model) whereas all other algae show a characteristic increase in that range (Figure 7). The same is true for the histogram of *Medicago trunculata *(MED_TRU) and *Carica papaya *(CAR_PAP) in comparison to other dicot species (Figure 8). In comparison, the histograms of well characterized plant genomes like *Arabidopsis thaliana *(ARA_THA) and *Zea mays *(ZEA_MAY) show a typical gamma distribution (Figure 8). It seems therefore plausible to suggest that the bioinformatic procedures that were used for annotating small proteins in the genomes of CHL_REI and MED_TRU were not as accurate as the procedures that were implemented in other plant species.

**Figure 11:**
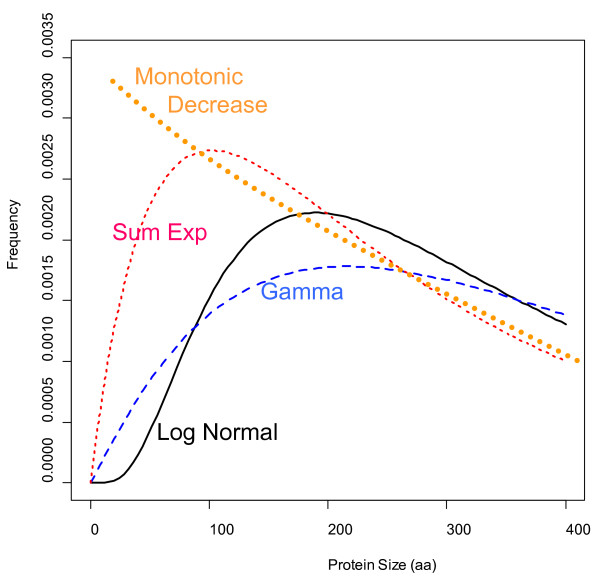
**Theoretical functions of protein size distribution**. One of the most notorious differences between the distribution models is how they start from cero. The monotonic decrease function is based on the occurrence of stop codons (3/64) along a random DNA sequence (random ORF). In the monotonic model, an ORF of size x is always more frequent than an ORF of size x + 1. The log normal function starts flat and then it increases sharply and peaks at ~200 aa. The gamma function starts steep, but then it peaks flatter at 200 aa than the other models. The sum of exponential function starts even steeper from cero and simulates somehow a monotonic function with a sharp cutoff of ORFs smaller than 33 aa (randomly expected ORFs). The model parameters to plot the density function in R were: dlnorm(x,5.772,0.729), dgamma(x,2.08,0.005), dsexp(x,0.01895,0.0042).

Considering these preliminary observations, we would like to speculate on the following: the most crude bioinformatic procedure simply detects all possible ORFs along the six frames of the genomic DNA sequence. This generates a protein size distribution that is monotonically decreasing (Figure 11). In order to filter out false positives, a sharp threshold is generally defined for ORFs smaller than 21-33 aa (randomly expected mean size). This generates a distribution that is similar as the one observed for MED_TRU (Figure 8). As more sophisticated procedures are applied for the detection of ORFs, the size distribution changes from a sum of exponentials, to a gamma or a log-normal function (Figure 11). A well annotated proteome will then generate a typical gamma distribution for protein size as observed for *Arabidopsis thaliana, Glycine max *and *Zea mays *(Figure 8).

More work is required to optimize bioinformatic procedures for correctly discriminating ORFs of biological active proteins and therefore filtering out false positives and baby ORFs of small size. One strategy would be to measure the nucleotide bias of the first and second codon positions [41]. Another complementary strategy would be to use the amino acid bias information in dependence of protein length and taxonomy (Figure 9) in order to discriminate baby ORFs from mummy ORFs [8]. Comparing the empirical size distribution to a log-normal or gamma function could then indicate how well a genomic release has been annotated.

### There are some significant amino acid bias depending on protein length and on species

It has been reported that the isoelectric point (pI) of proteins has a bimodal distribution, with very low fractions of proteins with pI close to 7.4 [46,47]. When all proteins are analyzed together, there is no correlation between protein size and pI values [46]. When acidic or basic proteins were analyzed separately, the correlation coefficient between protein size and pI was positive (r = 0.15) for the acidic set of proteins and negative (r = -0.25) for the basic set of proteins [[45] There is also a negative correlation between the pI bias of proteins and the genomic GC content [46]. We therefore asked whether protein size is correlated to the occurrence of certain amino acids influencing the pI values.

Previous studies have reported that there is a dependence of amino acid frequencies on sequence length [29]. Using a non-redundant set of only 2,275 proteins they found that the frequency of cysteine (C) increases dramatically for sequences shorter than 100 amino acids. It was also reported that arginine (R) and Lysine (K) frequencies increase for short sequences whereas aspartate (D) and glutamate (E) frequencies decrease [29]. In our datasets based on over 1.2 million proteins, we observed that the correlation of protein length with percentage amino acid composition is variable and not as restricted as postulated earlier [29]. Protein length correlates negatively with %C, %M, %K, %F, %R, %W, %Y (Figure 9). Positive correlations where detected for %D, %E, %Q, %S and %T (Figure 9). Rather variable, small or no significant correlations were found for %H, %I, and %V (Figure 9). It is noteworthy to mention that protein size correlated negatively with the basic amino acids (K and R) and positively with the acidic amino acids (D and E). This agrees with previous reports on the bimodal pI distribution, where acidic proteins are significantly longer than basic ones [46].

There are also some strong differences among phylogenetic groups. In archaea and bacteria, marked differences were found for the amino acids %E, %G, %K, %M as compared to the observed values in other eukaryotic groups (Figure 9). The positive correlation of %G is in accordance to the occurrence of large glycine rich proteins in bacterial genomes, a feature that seems specific for bacteria but not for eukaryotes (Figure 9. Thus, prokaryotic and eukaryotic proteins not only are different in size (Table 1 and Figure 2), but have also a different amino acid composition in dependence on protein length (Figure 9).

Interestingly, there were also some differences between plant groups. In monocot plants, protein length correlated negatively with %A, %G, %P. This was not the case in the dicot plant group. The opposite effect was observed for other amino acids where in monocot plants, protein length correlated positively with %F, %I, %K, %N. The reason for this is also unknown and should be investigated in more detail in follow-up studies. What is clear is that bacterial, animal and plant proteins have not only different average sizes, but have also specific biases towards different amino acids.

## Conclusions

In the present study, we demonstrate that proteins of different phylogenetic groups have different mean lengths. Some groups of species (e.g. protists, ciliophora, chlorophyta) have larger proteins than other groups of species (e.g. cnidaria, nematoda, placozoa, metazoa, viridiplantae). We found variable relationships between protein size, protein number or genomic %GC content. Among eukaryotes, protein number and protein size are negatively correlated (Figure 5, 6). The theoretical model of a gamma distribution with an unrestricted shape parameter can be a valuable tool to study protein evolution and to optimize automatic ORFs annotations.

Plants have usually more proteins in their genomes than animal species, but animal proteins are on average much larger. One possible reason for plants having smaller proteins than most animal species could be that plant proteins are encoded by less exons on average. We speculate that evolutionary forces related to functional domains in eukaryotic proteins explain the distribution of protein size in eukaryotes, but these hypotheses need to be tested statistically in some follow-up studies.

### Outlook

A comprehensive understanding of protein size across all taxonomic groups is relevant in the context of synthetic biology, which aims to construct minimal genomes and organisms. In addition to finding the minimal set of genes that are required to build a living cell (minimal number of proteins), it is also important to design synthetic proteins with minimal size (minimal aa usage) that still perform the desired biological function. Multi-domain proteins and multi-functional enzymes could be attractive strategies for synthetic DNA minimization.

## Methods

### Protein datafiles set 1

The protein sequences of all organisms where obtained from the NCBI and Ensemble public databases (download date February 2010), unless otherwise stated. We choose the species on following criteria: 1) full genomic versions containing all proteins of that species 2) sequence files publicly available. 3) focus primarily on all available higher plants species. We also included species from other taxonomic groups (animals, fungi, etc.) by selecting few representatives that also matched previous criteria. For a general comparison between eukaryotic and prokaryotic proteomes we therefore included 24 bacterial and 9 archaeal species in addition to the main 51 eukaryotic species.

The release version of the genomic sequences, the species IDs and the download sites of the protein fasta files are listed in Additional file 1: Table S1. In order to create a protein database that would be reliable for our purposes, we filtered the publicly available genomic protein fasta files for the presence of identical proteins, redundant, and thus created a database containing over 1.2 million non-redundant entries. The number of proteins in the original files (1,312,288), the duplicated genes (46,134) and the number of proteins finally kept for further analysis (1,266,154) are shown in Additional file 1: Table S2 for each species individually.

### Protein datafiles set 2

Available genomes from KEGG database (27 of May 2011; http://www.genome.jp/kegg/) were downloaded, and a database of protein sequences (in fasta format) from these genomes was constructed, comprising ~6.1 million entries. The taxonomic classification, KEGG code, number of proteins and gene-based G + C content is shown for each species in Additional file 1: Table S6.

### Sequence analysis and statistical procedures

*In house *developed perl scripts were used for sequence handling and analysis taking advantage of some standard BioPerl routines (http://www.bioperl.org/).

For all comparisons between samples, analysis of variance (ANOVA) or Linear Modelling (LM) were done with a threshold of *p *≤ 0.01 for statistical significance. In certain cases, we used Bonferroni type corrections for multiple comparisons, so that highly stringent p-values were used such as *p *≤ 0.00001. Statistical analysis was done mainly with the R program version 2.13 [48]. Analysis of variance (aov), Linear models (lm), Principal component analysis (pca), hierarchical clustering (hclust) and heatmap biclusters were done with R using the bioconductor, lattice and pcaMethods libraries with default settings for the *aov, lm, pca, histogram, hclust, dist, pairs, heatmap *and *plot *functions [48].

### Probability density functions for calculating theoretical protein length distribution

For describing the protein length distribution curves we chose some probability density functions that previous groups had chosen before: The gamma distribution [18], the sum of two independent exponential distributions [18] and the log-normal distribution [20,21]. For proteins larger than 1,500 aa we also analyzed the sequence length distributions using the Pareto's function [21].

#### The sum of two independent exponential random variables

The density function of the sum of two independent distributed random variables with parameters *α *> 0 and *β *> 0 respectively is given by:

(1) $$f_{Z}\left(z;\alpha ,\beta \right)=\frac{\alpha \beta }{\beta -\alpha }\left(e^{-\alpha z}-e^{-\beta z}\right)I_{\left(0,\infty \right)}\left(z\right),$$

where *z *is the protein length (total number of amino acid residues).

#### Gamma distributions

The gamma distribution has been used to model the protein length distributions in other works [19,20]. This distribution can be expressed in terms of a shape parameter (*θ*) and a scale parameter (*α*):

(2) $$f_{Z}\left(z;\theta ,\alpha \right)=\frac{\alpha^{\theta }z^{\theta -1}e^{-\alpha z}}{\Gamma \left(\theta \right)}I_{\left(0,\infty \right)}\left(z\right)$$

If *α = β*, then (1) reduces to:

(3) $$f_{Z}\left(z;\alpha \right)=\alpha^{2}ze^{-\alpha z}I_{\left(0,\infty \right)}\left(z\right)$$

One can obtain the same result by setting *θ *= 2 in (2), so it follows that the probability density function given in (3) corresponds to that of a Gamma random variable with scale parameter *α *and fixed shape parameter 2. The estimators of the parameters can be obtained easily using the maximum likelihood method with the moment's estimators as initial values.

#### Log normal distribution

The log-normal distribution can be expressed in terms of the parameters *μ *and *σ*:

(4) $$f_{Z}\left(z;\mu ,\sigma \right)=\frac{1}{\sqrt{2\pi }\sigma z}e^{-\frac{{\left(\log z-\mu \right)}^{2}}{2\sigma^{2}}}I_{\left(0,\infty \right)}\left(z\right)$$

This distribution can be used to approximate the frequency distributions of gamma distributed random variables since both distributions have the same support and similar asymmetries [49].

The log-normal function can be used to estimate expected values and the median as follows:

$$\begin{array}{c} E\left(Z\right)=e^{\mu +\sigma^{2}/2}\\ Me\left(Z\right)=e^{\mu } \end{array}$$

#### Pareto's Distribution

The Pareto's distribution is a power law probability distribution often used in social sciences and economics. This model has been used previously to describe the frequency distributions of protein lengths for the last few percentiles [21].

The Pareto's distribution can be expressed in terms of two parameters: *z_m _*(scale) and *α *(shape):

(5) $$f_{Z}\left(z;z_{m},\alpha \right)=\frac{\alpha z_{m}^{\alpha }}{z^{\alpha +1}}I_{\left(z_{m},\infty \right)}\left(z\right),$$

the corresponding distribution function is given by:

(6) $$P\left(Z\leq z\right)=1-\frac{z_{m}^{\alpha }}{z^{\alpha +1}}$$

so $P\left(Z>z\right)=\frac{z_{m}^{\alpha }}{z^{\alpha +1}}$, then taking natural logarithms in both sides leads to a linear model in *z*, log(*P*(*Z *>*z*)) = *αz_m _*- (*α *+ 1)log *z*. That means that under the null hypotheses the data comes from a Pareto's distribution, if one plots log(*P*(*Z *>*z*)) *vs *log *Z *the corresponding scatter plot must look like a straight line.

In the case of the Pareto's distribution the closed expressions for the parameter estimates are given by:

$${ẑ}_{m}=\underset{1\leq i\leq n}{\min}\left\{z_{i}\right\},\;\hat{\alpha }=\frac{n}{\overset{n}{\underset{i=1}{\sum }}\log\left(z_{i}-\log{ẑ}_{m}\right)}.$$

We applied the Chi-squared test to whether the data comes from the Pareto's distribution for each data set.

#### Model fitting

The statistical models described previously were fitted by using the well known maximum likelihood method for each species with the observed data in the range 0 <*z *< 1,500. In the case of the gamma and log normal distributions we used the *fitdistr *function in the MASS package [50] in R [48]. For the gamma distribution with fixed shape parameter and the distribution of the sum of two exponential distributions we used the *optim *function (also available in R) to maximize the likelihood function and obtain the estimates of the parameters of interest. The scripts used to fit all the models are available upon request.

#### Discriminating between models

The problem of choosing between rival models that are non-nested in terms of their functional forms has been studied by several authors [51]. Here we adopted the well known Akaike's information criterion (AIC) [52], given by:

(7) AIC=2k-2log(L)

where *k *is the number of parameters estimated in the model, and *log(L) *is the value of the log-likelihood function for the estimated parameters. For a given data set, several models can be fitted; according to the AIC criterion the model with smallest AIC is the best (more negative AIC value).

#### Testing whether the gamma type function has a fixed shape parameter equal to 2 or not

From the Large Sample Theory, it is well known that the maximum likelihood estimators are asymptotically normally distributed (e. g. Lehman, 1998, pag. 463, Theorem 5.1). Using this fact in the case of the gamma distribution for the shape parameter, $\sqrt{n}\left(\hat{\theta }-\theta \right)\overset{d}{\underset{}{\to }}N\left(0,{I^{-1}}_{\theta }\right)$ where $\hat{\theta }$ denotes the maximum likelihood estimator of *θ *and *I^-1^_θ _*denotes the entry corresponding to *θ *in the inverse of the Fisher information matrix. In the case of the protein length distributions for the 84 species it can be assumed that ${\hat{\theta }}_{j}\sim N\left(\theta ,\sigma_{j}^{2}\right),j=1,...,84$. Furthermore assuming that ${\hat{\theta }}_{1},\ldots ,{\hat{\theta }}_{84}$are independent random variables, then using the normal theory, $\bar{\hat{\theta }}=\frac{1}{84}\overset{84}{\underset{j=1}{\sum }}{\hat{\theta }}_{j}\sim N\left(\theta ,\frac{1}{84}\overset{84}{\underset{j=1}{\sum }}\sigma_{j}^{2}\right)$, the estimators of $\sigma_{j}^{2}$ can be obtained using the observed Fisher information matrix. Using this result one can easily test the following hypothesis set:

$$H_{0}:\;\theta =2\;vs\;H_{1}:\;\theta \neq 2$$

That is we want to know if the value hypothesized by White (1994) for the shape parameter 2 is feasible or not. This hypothesis can be easily tested using the abovementioned assumptions from the normal theory. If the obtained p-value is low enough, then the null hypothesis needs to be rejected, and that means that the gamma model with fixed shape parameter equal to 2 is inadequate.

## Competing interests

The authors declare that they have no competing interests.

## Authors' contributions

AT conceived of the study, coordinated the project, participated in the statistical analysis, prepared most figures, contributed to the statistical and biological interpretation of the results and wrote the manuscript. PPR performed statistical analysis, wrote the R scripts, carried out the model fittings and prepared some figures and tables. LDA obtained the sequence files of set 2, wrote the perl scripts for sequence analysis, and contributed to the statistical and biological interpretation of the data. All authors wrote and edited selected parts of the manuscript. They all revised and approved the final version.

## Supplementary Material

Additional file 1 **Suplemental tables and figures**. Table S1. Genomic download sites List of selected species, the respective genomic version and sites of download sites. File downloads were done between November 2009 and June 2010. Table S2. Total number of proteins Number of proteins used for statistical analysis. The publicly available protein fasta files were first formatted and filtered. Identical duplicates were discarded in order to keep a non-redundant protein set for each species. Table S3. Fitting parameters. Estimated parameters and AIC for the Gamma, lognormal and exponential sums for protein length distributions. Table S3. continued. Table S4. Protein length amino acid bias. The pearson correlation coefficients R of percentage amino acid composition with protein length were calculated for each species individually. Positive or negative R values indicate the direction of the correlation. Table S5. Parameter estimates for the Pareto's model and Chi-squared goodness of fit. Table S6. Taxonomic classification, KEGG code, species name, number of proteins and gene-based G+C content for each species of dataset 2. Figure S1. Histogram of the shape parameter in the modelled gamma functions. The distribution of the shape parameter values obtained in the modelled gamma functions. Figure S2. Dendogram of protein size attributes. Dendogram of protein size attributes in different species. Data from table 1 was used to construct a distance matrix for hierarchical clustering. Euclidean distances were calculated and then full hierarchical clustering was plotted with default parameters of the R function hclust(dist(data)). Figure S3. Pareto's best fit of the right handed distribution tail. Pareto's best fit for *Arabidopsis thaliana*. Click here for file
