# Supplementary material for: Mathematical modeling and comparison of protein size distribution in different plant, animal, fungal and microbial species reveals a negative correlation between protein size and protein number, thus providing insight into the evolution of proteomes
Source: BMC Res Notes. 2012 Feb 1;5:85. doi: 10.1186/1756-0500-5-85 (PMC3296660; doi:10.1186/1756-0500-5-85)
Supplement: Additional file 1 — Suplemental tables and figures. Table S1. Genomic download sites List of selected species, the respective genomic version and sites of download sites. File downloads were done between November 2009 and June 2010. Table S2. Total number of proteins Number of proteins used for statistical analysis. The publicly available protein fasta files were first formatted and filtered. Identical duplicates were discarded in order to keep a non-redundant protein set for each species. Table S3. Fitting parameters. Estimated parameters and AIC for the Gamma, lognormal and exponential sums for protein length distributions. Table S3. continued. Table S4. Protein length amino acid bias. The pearson correlation coefficients R of percentage amino acid composition with protein length were calculated for each species individually. Positive or negative R values indicate the direction of the correlation. Table S5. Parameter estimates for the Pareto's model and Chi-squared goodness of fit. Table S6. Taxonomic classification, KEGG code, species name, number of proteins and gene-based G+C content for each species of dataset 2. Figure S1. Histogram of the shape parameter in the modelled gamma functions. The distribution of the shape parameter values obtained in the modelled gamma functions. Figure S2. Dendogram of protein size attributes. Dendogram of protein size attributes in different species. Data from table 1 was used to construct a distance matrix for hierarchical clustering. Euclidean distances were calculated and then full hierarchical clustering was plotted with default parameters of the R function hclust(dist(data)). Figure S3. Pareto's best fit of the right handed distribution tail. Pareto's best fit for Arabidopsis thaliana. [file 1756-0500-5-85-S1.DOC]

# Supplementary Tables

Table S1 - Genomic download sites

List of selected species, the respective genomic version and sites of download sites. File downloads were done between November 2009 and June 2010.

| **Species Code** | **Species** | **Genomic**  **Version** | **Download site** |
| --- | --- | --- | --- |
| ACI_FER | Acidimicrobium ferrooxidans DSM 10331 | IMG/W 3.0 | http://img.jgi.doe.gov/cgi-bin/pub/main.cgi?section=TaxonDetail&page=taxonDetail&taxon_oid=644736322 |
| ANO_CAR | Anolis carolinensis | AnoCar1.0.57 | ftp://ftp.ensembl.org/pub/current_fasta/anolis_carolinensis/pep/Anolis_carolinensis.AnoCar1.0.57.pep.all.fa.gz |
| ANO_GAM | Anopheles gambiae | AgamP3 | ftp://ftp.ensemblgenomes.org/pub/metazoa/release-5/fasta/anopheles_gambiae/pep/Anopheles_gambiae.AgamP3.pep.all.fa.gz |
| ARA_THA | Arabidopsis thaliana | 9 | http://www.arabidopsis.org/ |
| ARC_PRO | Archaeoglobus profundus DSM5631 | 21/01/2010 | ftp://ftp.ncbi.nlm.nih.gov/genomes/Bacteria/Archaeoglobus_profundus_DSM_5631_uid43493/NC_013741.faa |
| BAC_FRA | Bacteroides fragilis NCTC 9343 | IMG/W 2.0 | http://img.jgi.doe.gov/cgi-bin/pub/main.cgi?section=TaxonDetail&taxon_oid=637000024 |
| BAC_SUB | Bacillus subtilis 168 | 06/03/2010 | ftp://ftp.ncbi.nih.gov/genbank/genomes/Bacteria/Bacillus_subtilis/AL009126.faa |
| BIF_ADO | Bifidobacterium adolescentis ATCC 15703 | IMG/W 2.1 | http://img.jgi.doe.gov/cgi-bin/pub/main.cgi?section=TaxonDetail&taxon_oid=639633010 |
| BOS_TAU | Bos taurus | Btau_4.0.57 | ftp://ftp.ensembl.org/pub/current_fasta/bos_taurus/pep/Bos_taurus.Btau_4.0.57.pep.all.fa.gz |
| BRA_DIS | Brachypodium distachyon | 01/05/2009 | http://files.brachypodium.org/Annotation/Bradi_1.0.pep.fa.gz |
| BRA_JAP | Bradyrhizobium japonicum USDA 110 | IMG/W 2.0 | http://img.jgi.doe.gov/cgi-bin/pub/main.cgi?section=TaxonDetail&taxon_oid=637000038 |
| BUR_CEP | Burkholderia cepacia AMMD | IMG/W 2.0 | http://img.jgi.doe.gov/cgi-bin/pub/main.cgi?section=TaxonDetail&taxon_oid=637000047 |
| CAE_ELE | Caenorhabditis elegans | WS120 | ftp://ftp.ensembl.org/pub/current_fasta/caenorhabditis_elegans/pep/Caenorhabditis_elegans.WS210.58.pep.all.fa.gz |
| CAM_JEJ | Campylobacter jejuni RM1221 | IMG/W 2.0 | http://img.jgi.doe.gov/cgi-bin/pub/main.cgi?section=TaxonDetail&taxon_oid=637000054 |
| CAN_KOR | Candidatus Korarchaeum cryptofilum OPF8 | IMG/W 2.6 | http://img.jgi.doe.gov/cgi-bin/pub/main.cgi?section=TaxonDetail&taxon_oid=641522611 |
| CAR_PAP | Carica papaya | 11/19/08 | ftp://asgpb.mhpcc.hawaii.edu/papaya/annotation/ (both contig (~3000 pep) and supercontig (~24000 pep) peps concat in a single file) |
| CEN_SYM | Cenarchaeum symbiosum A | IMG/W 2.6 | http://img.jgi.doe.gov/cgi-bin/pub/main.cgi?section=TaxonDetail&taxon_oid=641522613 |
| CHL_MUR | Chlamydia muridarum Nigg | IMG/W 2.0 | http://img.jgi.doe.gov/cgi-bin/pub/main.cgi?section=TaxonDetail&taxon_oid=637000062 |
| CHL_REI | Chlamydomonas reinhardtii | 4 | http://genome.jgi-psf.org/Chlre4/download/annotation/Chlre4_best_proteins.fasta.gz |
| COR_AUR | Corynebacterium aurimucosum ATCC 700975 | IMG/W 2.9 | http://img.jgi.doe.gov/cgi-bin/pub/main.cgi?section=TaxonDetail&taxon_oid=643692018 |
| CRY_PAR | Cryptosporidium parvum | Integr8(29062010) | ftp://ftp.ebi.ac.uk/pub/databases/integr8/fasta/proteomes/28963.C_parvum.fasta.gz |
| CYA_MER | Cyanidioschyzon merolae | 13/04/2004 | http://merolae.biol.s.u-tokyo.ac.jp/ |
| DAN_RER | Danio rerio | Zv8.57 | ftp://ftp.ensembl.org/pub/current_fasta/danio_rerio/pep/Danio_rerio.Zv8.57.pep.all.fa.gz |
| DEI_DES | Deinococcus deserti VCD115 | IMG/W 2.9 | http://img.jgi.doe.gov/cgi-bin/pub/main.cgi?section=TaxonDetail&taxon_oid=643692020 |
| DES_KAM | Desulfurococcus kamchatkensis 1221n | IMG/W 2.8 | http://img.jgi.doe.gov/cgi-bin/pub/main.cgi?section=TaxonDetail&taxon_oid=643348540 |
| DIC_DIS | Dictyostelium discoideum | 06-27-2010 | http://dictybase.org/db/cgi-bin/dictyBase/download/download.pl?area=blast_databases |
| DRO_MEL | Drosophila melanogaster | BDGP5.13.57 | ftp://ftp.ensembl.org/pub/current_fasta/drosophila_melanogaster/pep/Drosophila_melanogaster.BDGP5.13.57.pep.all.fa.gz |
| EQU_CAB | Equus caballus | EquCab2.57 | ftp://ftp.ensembl.org/pub/current_fasta/equus_caballus/pep/Equus_caballus.EquCab2.57.pep.all.fa.gz |
| ESC_COL | Escherichia coli O157:H7 str. EC4115 | 28/01/2010 | ftp://ftp.tigr.org/pub/data/Microbial_Genomes/e_coli_o157_h7_str_ec4115/annotation_dbs/e_coli_o157_h7_str_ec4115.pep |
| GAL_GAL | Gallus gallus | WASHUC2.57 | ftp://ftp.ensembl.org/pub/current_fasta/gallus_gallus/pep/Gallus_gallus.WASHUC2.57.pep.all.fa.gz |
| GLO_VIO | Gloeobacter violaceus PCC 7421 | 16/11/2004 | ftp://ftp.kazusa.or.jp/pub/CyanoBase/Gloeobacter/chromo.p.aa.gz |
| GLY_MAX | Glycine max | 1 | ftp://ftp.jgi-psf.org/pub/JGI_data/phytozome/v5.0/Gmax/annotation/Glyma1_highConfidence.pep.fa.gz |
| GUI_LAM | Giardia lamblia | GiardiaDB-2.1 | http://giardiadb.org/common/downloads/release-2.1/GintestinalisAssemblageA/GlambliaAnnotatedProteins_GiardiaDB-2.1.fasta |
| HOM_SAP | Homo sapiens | GRCh37.57 | ftp://ftp.ensembl.org/pub/current_fasta/homo_sapiens/pep/Homo_sapiens.GRCh37.57.pep.all.fa.gz |
| HYD_THE | Hydrogenobacter thermophilus TK-6 | IMG/W 3.1 | http://img.jgi.doe.gov/cgi-bin/pub/main.cgi?section=TaxonDetail&taxon_oid=646311936 |
| KOC_RHI | Kocuria rhizophila DC2201 | IMG/W 2.7 | http://img.jgi.doe.gov/cgi-bin/pub/main.cgi?section=TaxonDetail&taxon_oid=642555133 |
| LAC_BIC | Laccaria bicolor | 1 | ftp://ftp.jgi-psf.org/pub/JGI_data/Laccaria_bicolor/BestModelsv1.aa.fasta.gz |
| LEP_BIF | Leptospira biflexa Patoc 1 (Ames) | IMG/W 2.7 | http://img.jgi.doe.gov/cgi-bin/pub/main.cgi?section=TaxonDetail&taxon_oid=642555136 |
| MED_TRU | Medicagao truncatula | 3 | http://www.medicago.org/genome/downloads/Mt3/Medicago_3.0_annotation.tar.gz (Not public yet, we must check before publishing our results if we can include it or not) |
| MEL_GAL | Meleagris gallopavo | UMD2.57 | ftp://ftp.ensembl.org/pub/current_fasta/meleagris_gallopavo/pep/Meleagris_gallopavo.UMD2.57.pep.all.fa.gz |
| MET_JAN | Methanococcus jannaschii | 09/08/1999 | http://bioinfo.mbb.yale.edu/genome/db99/Mjan/aaseq.fa |
| MIC_CCM | Micromonas CCMP1545 | 2 | ftp://ftp.jgi-psf.org/pub/JGI_data/Micromonas_pusilla_CCMP1545/v2.0/MicromonasCCMP1545.FilteredModels2.aa.fasta.gz |
| MIC_RCC | Micromonas RCC299 | 3 | http://genome.jgi-psf.org/MicpuN3/download/MicromonasRCC299v3.FrozenGeneCatalog_20090404.proteins.fasta.gz |
| MON_DOM | Monodelphis domestica | BROADO5.57 | ftp://ftp.ensembl.org/pub/current_fasta/monodelphis_domestica/pep/Monodelphis_domestica.BROADO5.57.pep.all.fa.gz |
| MYC_ABS | Mycobacterium abscessus | IMG/W 2.6 | http://img.jgi.doe.gov/cgi-bin/pub/main.cgi?section=TaxonDetail&taxon_oid=641522641 |
| NAN_EQU | Nanoarchaeum equitans Kin4-M | IMG/W 2.0 | http://img.jgi.doe.gov/cgi-bin/pub/main.cgi?section=TaxonDetail&taxon_oid=638154511 |
| NEM_VEC | Nematostella vectensis | 1 | ftp://ftp.jgi-psf.org/pub/JGI_data/Nematostella_vectensis/v1.0/annotation/proteins.Nemve1FilteredModels1.fasta.gz |
| ORN_ANA | Ornithorhynchus anatinus | OANA5.57 | ftp://ftp.ensembl.org/pub/current_fasta/ornithorhynchus_anatinus/pep/Ornithorhynchus_anatinus.OANA5.57.pep.all.fa.gz |
| ORY_SAT | Oryza sativa ssp. japonica | 6.1 | ftp://ftp.plantbiology.msu.edu/pub/data/Eukaryotic_Projects/o_sativa/annotation_dbs/pseudomolecules/version_6.1/all.dir/all.pep |
| OST_LUC | Ostreococcus lucimarinus | 3 | ftp://ftp.jgi-psf.org/pub/JGI_data/Ostreococcus_lucimarinus/O.lucimarinus.FM.aa.fasta.gz |
| OST_TAU | Ostreococcus tauri | 4 | ftp://ftp.jgi-psf.org/pub/JGI_data/Ostreococcus_tauri/O.tauri.FM.aa.fasta.gz |
| PAR_TET | Paramecium tetraurelia | v1.43 | http://paramecium.cgm.cnrs-gif.fr/download/fasta/Ptetraurelia_peptides_v1.43.fasta |
| PER_MAR | Persephonella marina EX-H1 | IMG/W 2.9 | http://img.jgi.doe.gov/cgi-bin/pub/main.cgi?section=TaxonDetail&taxon_oid=643692030 |
| PHA_CHR | Phanerochaete chrysosporium strain RP78 | 2.1 | ftp://ftp.jgi-psf.org/pub/JGI_data/Phanerochaete_chrysosporium/v2.0/BestModels2.1.prot.gz |
| PHA_TRI | Phaeodactylum tricornutum | 2 | http://genome.jgi-psf.org/Phatr2/download/Phatr2_chromosomes_geneModels_FilteredModels2_aa.fasta.gz |
| PHY_PAT | Physcomitrella patens | 1.1 | ftp://ftp.jgi-psf.org/pub/JGI_data/Physcomitrella_patens/v1.1/proteins.Phypa1_1.FilteredModels.fasta.gz |
| PHY_RAM | Phytophthora ramorum | 1.1 | ftp://ftp.jgi-psf.org/pub/JGI_data/Pramorum/assembly/v1.1/proteins.FM_Phyra1_1.fasta.gz |
| PHY_SOJ | Phytophthora sojae | 1.1 | ftp://ftp.jgi-psf.org/pub/JGI_data/Psojae/assembly/v1.1/proteins.FM_Physo1_1.fasta.gz |
| PIC_STI | Pichia stipitis | 3 | http://genome.jgi-psf.org/Picst3/download/v2.0/Pstipitisv2.FilteredModels1.proteins.gz |
| PLA_FAL | Plasmodium falciparum | 3D7v2.1.1 | ftp://ftp.sanger.ac.uk/pub/pathogens/Plasmodium/falciparum/3D7/3D7.version2.1.1/3D7.version.2.1.1.proteins.fasta |
| POP_TRI | Populus trichocarpa | 2 | ftp://ftp.jgi-psf.org/pub/JGI_data/phytozome/v5.0/Ptrichocarpa/annotation/Populus.trichocarpa.v2.0.peptide.fa.gz |
| PRI_PAC | Pristionchus pacificus | 29062010 | ftp://ftp.ensemblgenomes.org/pub/metazoa/release-5/fasta/pristionchus_pacificus/pep/Pristionchus_pacificus.pp1.pep.all.fa.gz |
| RAT_NOR | Rattus norvegicus | RGSC3.4.57 | ftp://ftp.ensembl.org/pub/current_fasta/rattus_norvegicus/pep/Rattus_norvegicus.RGSC3.4.57.pep.all.fa.gz |
| SAC_CER | Saccharomyces cerevisiae | 05/01/2010 | http://downloads.yeastgenome.org/sequence/genomic_sequence/orf_protein/orf_trans.fasta.gz |
| SEL_MOE | Selaginella moellendorfii | 1 | http://genome.jgi-psf.org/Selmo1/download/Selmo1_GeneModels_FilteredModels3_aa.fasta.gz |
| SOR_BIC | Sorghum bicolor | 1 | ftp://ftp.jgi-psf.org/pub/JGI_data/Sorghum_bicolor/v1.0/Sorbi1_GeneModels_FilteredModels6_aa.fasta.gz |
| STA_AUR | Staphylococcus aureus aureus MW2 | IMG/W 2.0 | http://img.jgi.doe.gov/cgi-bin/pub/main.cgi?section=TaxonDetail&taxon_oid=637000276 |
| STR_AVE | Streptomyces avermitilis MA-4680 | IMG/W 2.0 | http://img.jgi.doe.gov/cgi-bin/pub/main.cgi?section=TaxonDetail&taxon_oid=637000304 |
| SUL_ACI | Sulfolobus acidocaldarius DSM 639 | IMG/W 2.0 | http://img.jgi.doe.gov/cgi-bin/pub/main.cgi?section=TaxonDetail&taxon_oid=638154517 |
| SUL_DEL | Sulfurospirillum deleyianum DSM 6946 | MG/W 3.1 | http://img.jgi.doe.gov/cgi-bin/pub/main.cgi?section=TaxonDetail&taxon_oid=646311960 |
| SYN_SP | Synechocystis sp. PCC 6803 | 11/05/2009 | ftp://ftp.kazusa.or.jp/pub/CyanoBase/Synechocystis/Synecho.p.aa.gz |
| TAK_RUB | Takifugu rubripes | FUGU4.57 | ftp://ftp.ensembl.org/pub/current_fasta/takifugu_rubripes/pep/Takifugu_rubripes.FUGU4.57.pep.all.fa.gz |
| TET_THE | Tetrahymena thermophila | Oct-08 | ftp://ftp.tigr.org/pub/data/Eukaryotic_Projects/t_thermophila/annotation_dbs/final_release_oct2008/tta1_oct2008_finalrelease.aa.fsa |
| THA_PSE | Thalassiosira pseudonana | 3 | http://genome.jgi-psf.org/Thaps3/download/Thaps3_chromosomes_geneModels_FilteredModels2_aa.fasta.gz |
| THE_ELO | Thermosynechococcus elongatus BP-1 | 26/12/2007 | ftp://ftp.kazusa.or.jp/pub/CyanoBase/Thermo/chromo.p.aa.gz |
| THE_NEU | Thermoproteus neutrophilus V24Sta | IMG/W 2.6 | http://img.jgi.doe.gov/cgi-bin/pub/main.cgi?section=TaxonDetail&taxon_oid=641522657 |
| THE_THE | Thermus thermophilus HB27 | IMG/W 2.0 | http://img.jgi.doe.gov/cgi-bin/pub/main.cgi?section=TaxonDetail&taxon_oid=637000322 |
| THE_VOL | Thermoplasma volcanium GSS1 | IMG/W 2.0 | http://img.jgi.doe.gov/cgi-bin/pub/main.cgi?section=TaxonDetail&taxon_oid=638154522 |
| TOX_GON | Toxoplasma gondii | ToxoDB-6.0 | http://toxodb.org/common/downloads/release-6.0/Tgondii/TgondiiGT1AnnotatedProteins_ToxoDB-6.0.fasta |
| TRI_ADH | Trichoplax adhaerens | 1 | ftp://ftp.jgi-psf.org/pub/JGI_data/Trichoplax_adhaerens_Grell-BS-1999/annotation/v1.0/Triad1_best_proteins.fasta.gz |
| TRI_REE | Trichoderma reesei | 2 | http://genome.jgi-psf.org/Trire2/download/TreeseiV2_FilteredModelsv2.0.proteins.fasta.gz |
| UST_MAY | Ustilago maydis | 2 | http://www.broadinstitute.org/annotation/genome/ustilago_maydis.2/download/?sp=EAProteinsFasta&sp=SUM1&sp=S.zip |
| XAN_CAM | Xanthomonas campestris pv armoraciae | CMRv24 (date 24/06/2010) | ftp://ftp.jcvi.org/pub/data/Microbial_Genomes/x_campestris_pv_armoraciae_756c/annotation_dbs/ |
| ZEA_MAY | Zea mays | 4a.53 | http://ftp.maizesequence.org/current/filtered-set/ZmB73_4a.53_filtered_translations.fasta.gz |

Table S2 - Total number of proteins

Number of proteins used for statistical analysis. The publicly available protein fasta files were first formatted and filtered. Identical duplicates were discarded in order to keep a non-redundant protein set for each species.

| **Species Code** | **Total** | **Duplicates** | **Kept** |
| --- | --- | --- | --- |
| ACI_FER | 2038 | 29 | 2009 |
| ANO_CAR | 17672 | 31 | 17641 |
| ANO_GAM | 14103 | 588 | 13515 |
| ARA_THA | 33410 | 2189 | 31221 |
| ARC_PRO | 1819 | 1 | 1818 |
| BAC_FRA | 4299 | 1 | 4298 |
| BAC_SUB | 4177 | 1 | 4176 |
| BIF_ADO | 1631 | 3 | 1628 |
| BOS_TAU | 26977 | 699 | 26278 |
| BRA_DIS | 32255 | 2094 | 30161 |
| BRA_JAP | 8317 | 61 | 8256 |
| BUR_CEP | 6634 | 10 | 6624 |
| CAE_ELE | 27975 | 3883 | 24092 |
| CAM_JEJ | 1880 | 2 | 1878 |
| CAN_KOR | 1612 | 0 | 1612 |
| CAR_PAP | 27950 | 130 | 27820 |
| CEN_SYM | 2017 | 3 | 2014 |
| CHL_MUR | 911 | 0 | 911 |
| CHL_REI | 16709 | 331 | 16378 |
| COR_AUR | 2551 | 23 | 2528 |
| CRY_PAR | 3805 | 0 | 3805 |
| CYA_MER | 5014 | 12 | 5002 |
| DAN_RER | 28630 | 1144 | 27486 |
| DEI_DES | 3451 | 7 | 3444 |
| DES_KAM | 1471 | 1 | 1470 |
| DIC_DIS | 13318 | 370 | 12948 |
| DRO_MEL | 21309 | 3085 | 18224 |
| EQU_CAB | 22641 | 163 | 22478 |
| ESC_COL | 5837 | 199 | 5638 |
| GAL_GAL | 22194 | 328 | 21866 |
| GLO_VIO | 4431 | 21 | 4410 |
| GLY_MAX | 46384 | 251 | 46133 |
| GUI_LAM | 5901 | 181 | 5720 |
| HOM_SAP | 76592 | 13514 | 63078 |
| HYD_THE | 1893 | 2 | 1891 |
| KOC_RHI | 2357 | 4 | 2353 |
| LAC_BIC | 20614 | 398 | 20216 |
| LEP_BIF | 3600 | 5 | 3595 |
| MED_TRU | 53423 | 2240 | 51183 |
| MEL_GAL | 17210 | 3 | 17207 |
| MET_JAN | 1771 | 1 | 1770 |
| MIC_CCM | 10475 | 20 | 10455 |
| MIC_RCC | 10109 | 38 | 10071 |
| MON_DOM | 32541 | 119 | 32422 |
| MYC_ABS | 4941 | 2 | 4939 |
| NAN_EQU | 556 | 0 | 556 |
| NEM_VEC | 27273 | 610 | 26663 |
| ORN_ANA | 26836 | 87 | 26749 |
| ORY_SAT | 67393 | 4178 | 63215 |
| OST_LUC | 7651 | 208 | 7443 |
| OST_TAU | 7725 | 10 | 7715 |
| PAR_TET | 39541 | 312 | 39229 |
| PER_MAR | 2051 | 3 | 2048 |
| PHA_CHR | 10048 | 52 | 9996 |
| PHA_TRI | 10025 | 52 | 9973 |
| PHY_PAT | 35938 | 341 | 35597 |
| PHY_RAM | 15743 | 260 | 15483 |
| PHY_SOJ | 19027 | 264 | 18763 |
| PIC_STI | 5839 | 18 | 5821 |
| PLA_FAL | 5405 | 22 | 5383 |
| POP_TRI | 45778 | 1425 | 44353 |
| PRI_PAC | 29644 | 134 | 29510 |
| RAT_NOR | 32971 | 191 | 32780 |
| SAC_CER | 5885 | 63 | 5822 |
| SEL_MOE | 22285 | 147 | 22138 |
| SOR_BIC | 35899 | 339 | 35560 |
| STA_AUR | 2632 | 0 | 2632 |
| STR_AVE | 7673 | 9 | 7664 |
| SUL_ACI | 2285 | 0 | 2285 |
| SUL_DEL | 2291 | 22 | 2269 |
| SYN_SP | 3264 | 27 | 3237 |
| TAK_RUB | 47841 | 113 | 47728 |
| TET_THE | 24725 | 27 | 24698 |
| THA_PSE | 11390 | 50 | 11340 |
| THE_ELO | 2477 | 23 | 2454 |
| THE_NEU | 2006 | 1 | 2005 |
| THE_THE | 2210 | 9 | 2201 |
| THE_VOL | 1561 | 2 | 1559 |
| TOX_GON | 8102 | 2 | 8100 |
| TRI_ADH | 11520 | 17 | 11503 |
| TRI_REE | 9129 | 1 | 9128 |
| UST_MAY | 6522 | 4 | 6518 |
| XAN_CAM | 4534 | 25 | 4509 |
| ZEA_MAY | 53764 | 4899 | 48865 |

Table S3 - Fitting parameters

Estimated parameters and AIC for the Gamma, lognormal and exponential sums for protein length distributions.

| **Species** | **Exponential sums** | | | |  | **Gamma** | | | |
| --- | --- | --- | --- | --- | --- | --- | --- | --- | --- |
|  | $\alpha$ | $\beta$ | **Log-lik** | **AIC** | $\theta$ | sd | $\alpha$ | **Log-lik** | **AIC** |
| ACI_FER | 0.006 | 0.006 | -13210 | 26424 | 2.547 | 0.0722 | 0.008 | -13160 | 26324 |
| ANO_CAR | 0.005 | 0.005 | -117666 | 235336 | 2.114 | 0.0195 | 0.005 | -117605 | 235214 |
| ANO_GAM | 0.004 | 0.004 | -89982 | 179968 | 2.135 | 0.0223 | 0.005 | -89944 | 179892 |
| ARA_THA | 0.005 | 0.005 | -210609 | 421221 | 2.365 | 0.0166 | 0.006 | -210387 | 420778 |
| ARC_PRO | 0.008 | 0.008 | -11633 | 23269 | 2.084 | 0.0620 | 0.008 | -11627 | 23258 |
| BAC_FRA | 0.006 | 0.006 | -28847 | 57697 | 2.191 | 0.0413 | 0.006 | -28824 | 57652 |
| BAC_SUB | 0.007 | 0.007 | -26934 | 53872 | 2.339 | 0.0462 | 0.008 | -26887 | 53778 |
| BIF_ADO | 0.006 | 0.006 | -10820 | 21645 | 2.989 | 0.0947 | 0.008 | -10734 | 21473 |
| BOS_TAU | 0.005 | 0.005 | -175711 | 351426 | 2.335 | 0.0177 | 0.005 | -175412 | 350828 |
| BRA_DIS | 0.005 | 0.005 | -203772 | 407547 | 2.639 | 0.0189 | 0.006 | -202893 | 405790 |
| BRA_JAP | 0.006 | 0.006 | -53873 | 107750 | 2.615 | 0.0368 | 0.008 | -53617 | 107238 |
| BUR_CEP | 0.006 | 0.006 | -43264 | 86532 | 2.724 | 0.0429 | 0.009 | -43020 | 86044 |
| CAE_ELE | 0.005 | 0.005 | -160515 | 321034 | 2.262 | 0.0180 | 0.006 | -160300 | 320604 |
| CAM_JEJ | 0.007 | 0.007 | -12236 | 24476 | 2.213 | 0.0646 | 0.008 | -12225 | 24455 |
| CAN_KOR | 0.007 | 0.007 | -10420 | 20843 | 2.679 | 0.0859 | 0.009 | -10359 | 20722 |
| CAR_PAP | 0.019 | 0.004 | -183212 | 366428 | 1.554 | 0.0113 | 0.005 | -183375 | 366754 |
| CEN_SYM | 0.012 | 0.006 | -12736 | 25476 | 1.713 | 0.0478 | 0.007 | -12765 | 25534 |
| CHL_MUR | 0.006 | 0.006 | -6041 | 12086 | 2.150 | 0.0887 | 0.006 | -6037 | 12078 |
| CHL_REI | 0.011 | 0.003 | -107663 | 215330 | 1.674 | 0.0156 | 0.004 | -107763 | 215530 |
| COR_AUR | 0.006 | 0.006 | -16580 | 33164 | 2.509 | 0.0634 | 0.008 | -16523 | 33049 |
| CRY_PAR | 0.004 | 0.004 | -25237 | 50479 | 2.331 | 0.0459 | 0.005 | -25201 | 50407 |
| CYA_MER | 0.004 | 0.004 | -33912 | 67828 | 2.739 | 0.0479 | 0.006 | -33792 | 67587 |
| DAN_RER | 0.005 | 0.005 | -184072 | 368149 | 2.437 | 0.0182 | 0.006 | -183583 | 367170 |
| DEI_DES | 0.006 | 0.006 | -22433 | 44870 | 2.596 | 0.0566 | 0.008 | -22316 | 44636 |
| DES_KAM | 0.007 | 0.007 | -9506 | 19016 | 2.095 | 0.0691 | 0.008 | -9503 | 19011 |
| DIC_DIS | 0.010 | 0.003 | -87530 | 175063 | 1.822 | 0.0188 | 0.004 | -87513 | 175030 |
| DRO_MEL | 0.004 | 0.004 | -120830 | 241665 | 2.268 | 0.0204 | 0.005 | -120754 | 241512 |
| EQU_CAB | 0.004 | 0.004 | -148907 | 297817 | 2.235 | 0.0181 | 0.005 | -148763 | 297529 |
| ESC_COL | 0.012 | 0.005 | -36822 | 73647 | 1.745 | 0.0288 | 0.006 | -36834 | 73672 |
| GAL_GAL | 0.008 | 0.003 | -145633 | 291270 | 1.827 | 0.0148 | 0.004 | -145675 | 291355 |
| GLO_VIO | 0.007 | 0.006 | -28873 | 57750 | 2.081 | 0.0392 | 0.007 | -28858 | 57720 |
| GLY_MAX | 0.005 | 0.005 | -311006 | 622015 | 2.602 | 0.0151 | 0.006 | -309831 | 619666 |
| GUI_LAM | 0.014 | 0.003 | -37880 | 75765 | 1.727 | 0.0271 | 0.004 | -37877 | 75759 |
| HOM_SAP | 0.011 | 0.003 | -418106 | 836216 | 1.650 | 0.0078 | 0.004 | -418445 | 836893 |
| HYD_THE | 0.007 | 0.007 | -12280 | 24563 | 2.274 | 0.0663 | 0.008 | -12258 | 24519 |
| KOC_RHI | 0.006 | 0.006 | -15538 | 31080 | 2.649 | 0.0694 | 0.008 | -15469 | 30942 |
| LAC_BIC | 0.010 | 0.004 | -135415 | 270833 | 1.746 | 0.0148 | 0.005 | -135542 | 271087 |
| LEP_BIF | 0.006 | 0.006 | -23680 | 47364 | 2.689 | 0.0572 | 0.008 | -23547 | 47098 |
| MED_TRU | 0.031 | 0.005 | -326850 | 653704 | 1.111 | 0.0058 | 0.005 | -330039 | 660083 |
| MEL_GAL | 0.006 | 0.004 | -114932 | 229868 | 1.953 | 0.0180 | 0.005 | -114943 | 229889 |
| MET_JAN | 0.007 | 0.007 | -11356 | 22715 | 2.351 | 0.0714 | 0.008 | -11320 | 22644 |
| MIC_CCM | 0.005 | 0.005 | -69892 | 139788 | 2.240 | 0.0270 | 0.006 | -69803 | 139610 |
| MIC_RCC | 0.005 | 0.005 | -67356 | 134715 | 2.385 | 0.0293 | 0.006 | -67198 | 134399 |
| MON_DOM | 0.004 | 0.004 | -218538 | 437080 | 2.816 | 0.0193 | 0.006 | -217819 | 435642 |
| MYC_ABS | 0.006 | 0.006 | -32074 | 64152 | 2.655 | 0.0485 | 0.009 | -31883 | 63770 |
| NAN_EQU | 0.007 | 0.007 | -3565 | 7134 | 2.143 | 0.1153 | 0.008 | -3561 | 7125 |
| NEM_VEC | 0.010 | 0.005 | -174039 | 348082 | 1.712 | 0.0129 | 0.005 | -174446 | 348897 |
| ORN_ANA | 0.005 | 0.005 | -178422 | 356848 | 2.075 | 0.0155 | 0.005 | -178363 | 356730 |
| ORY_SAT | 0.009 | 0.003 | -421997 | 843998 | 1.783 | 0.0085 | 0.004 | -422354 | 844713 |
| OST_LUC | 0.005 | 0.005 | -49541 | 99086 | 2.248 | 0.0323 | 0.006 | -49464 | 98932 |
| OST_TAU | 0.006 | 0.006 | -51254 | 102512 | 2.141 | 0.0302 | 0.006 | -51212 | 102427 |
| PAR_TET | 0.005 | 0.005 | -259610 | 519224 | 2.218 | 0.0139 | 0.006 | -259325 | 518653 |
| PER_MAR | 0.007 | 0.007 | -13365 | 26733 | 2.182 | 0.0608 | 0.007 | -13350 | 26703 |
| PHA_CHR | 0.005 | 0.005 | -67786 | 135577 | 2.617 | 0.0324 | 0.006 | -67472 | 134948 |
| PHA_TRI | 0.005 | 0.005 | -67360 | 134723 | 2.692 | 0.0335 | 0.006 | -67004 | 134013 |
| PHY_PAT | 0.007 | 0.005 | -235956 | 471915 | 1.956 | 0.0128 | 0.006 | -236022 | 472049 |
| PHY_RAM | 0.005 | 0.005 | -104245 | 208494 | 2.440 | 0.0241 | 0.006 | -103941 | 207885 |
| PHY_SOJ | 0.004 | 0.004 | -126257 | 252519 | 2.272 | 0.0202 | 0.005 | -126099 | 252203 |
| PIC_STI | 0.004 | 0.004 | -39603 | 79210 | 2.857 | 0.0465 | 0.006 | -39398 | 78800 |
| PLA_FAL | 0.004 | 0.004 | -33554 | 67111 | 2.009 | 0.0334 | 0.004 | -33553 | 67111 |
| POP_TRI | 0.005 | 0.006 | -297514 | 595033 | 2.003 | 0.0116 | 0.006 | -297511 | 595027 |
| PRI_PAC | 0.015 | 0.005 | -190375 | 380753 | 1.484 | 0.0105 | 0.005 | -191251 | 382506 |
| RAT_NOR | 0.004 | 0.004 | -218651 | 437307 | 2.130 | 0.0143 | 0.005 | -218588 | 437180 |
| SAC_CER | 0.004 | 0.004 | -39421 | 78846 | 2.440 | 0.0391 | 0.005 | -39385 | 78774 |
| SEL_MOE | 0.005 | 0.005 | -147588 | 295180 | 2.321 | 0.0194 | 0.006 | -147271 | 294546 |
| SOR_BIC | 0.006 | 0.005 | -237403 | 474809 | 1.984 | 0.0129 | 0.006 | -237419 | 474841 |
| STA_AUR | 0.007 | 0.007 | -17098 | 34199 | 2.167 | 0.0534 | 0.007 | -17085 | 34173 |
| STR_AVE | 0.006 | 0.006 | -50255 | 100515 | 2.589 | 0.0377 | 0.008 | -50029 | 100063 |
| SUL_ACI | 0.007 | 0.007 | -14763 | 29529 | 2.426 | 0.0649 | 0.009 | -14717 | 29439 |
| SUL_DEL | 0.006 | 0.006 | -14819 | 29642 | 2.480 | 0.0663 | 0.008 | -14773 | 29550 |
| SYN_SP | 0.006 | 0.006 | -21163 | 42331 | 2.137 | 0.0472 | 0.007 | -21146 | 42297 |
| TAK_RUB | 0.004 | 0.004 | -319891 | 639786 | 3.153 | 0.0179 | 0.006 | -317590 | 635185 |
| TET_THE | 0.006 | 0.003 | -161447 | 322898 | 1.944 | 0.0147 | 0.004 | -161417 | 322837 |
| THA_PSE | 0.004 | 0.004 | -76388 | 152779 | 2.528 | 0.0292 | 0.006 | -76145 | 152293 |
| THE_ELO | 0.006 | 0.006 | -16104 | 32212 | 2.253 | 0.0573 | 0.007 | -16081 | 32167 |
| THE_NEU | 0.007 | 0.008 | -12816 | 25636 | 2.337 | 0.0668 | 0.009 | -12781 | 25566 |
| THE_THE | 0.007 | 0.007 | -14251 | 28506 | 2.772 | 0.0762 | 0.009 | -14170 | 28344 |
| THE_VOL | 0.007 | 0.007 | -10119 | 20242 | 2.419 | 0.0781 | 0.008 | -10088 | 20181 |
| TOX_GON | 0.004 | 0.004 | -51553 | 103110 | 2.016 | 0.0272 | 0.004 | -51551 | 103107 |
| TRI_ADH | 0.005 | 0.005 | -76705 | 153413 | 2.325 | 0.0268 | 0.006 | -76527 | 153057 |
| TRI_REE | 0.004 | 0.004 | -61786 | 123576 | 2.773 | 0.0361 | 0.006 | -61522 | 123049 |
| UST_MAY | 0.004 | 0.004 | -44152 | 88309 | 2.754 | 0.0414 | 0.005 | -44024 | 88052 |
| XAN_CAM | 0.012 | 0.005 | -29844 | 59692 | 1.745 | 0.0319 | 0.006 | -29848 | 59700 |
| ZEA_MAY | 0.006 | 0.006 | -325054 | 650111 | 2.085 | 0.0117 | 0.006 | -324969 | 649941 |

Table S3 – continued

| Species | Lognormal | | | |
| --- | --- | --- | --- | --- |
|  | $\mu$ | $\sigma$ | Log-lik | AIC |
| ACI_FER | 5.582 | 0.646 | -13180 | 26365 |
| ANO_CAR | 5.787 | 0.704 | -117534 | 235071 |
| ANO_GAM | 5.863 | 0.722 | -90006 | 180016 |
| ARA_THA | 5.736 | 0.722 | -211388 | 422779 |
| ARC_PRO | 5.340 | 0.692 | -11606 | 23216 |
| BAC_FRA | 5.651 | 0.711 | -28866 | 57736 |
| BAC_SUB | 5.431 | 0.688 | -26940 | 53884 |
| BIF_ADO | 5.731 | 0.591 | -10744 | 21491 |
| BOS_TAU | 5.864 | 0.689 | -175850 | 351704 |
| BRA_DIS | 5.836 | 0.629 | -202826 | 405656 |
| BRA_JAP | 5.560 | 0.623 | -53591 | 107186 |
| BUR_CEP | 5.592 | 0.617 | -43054 | 86113 |
| CAE_ELE | 5.766 | 0.690 | -160482 | 320967 |
| CAM_JEJ | 5.449 | 0.719 | -12266 | 24536 |
| CAN_KOR | 5.508 | 0.605 | -10342 | 20689 |
| CAR_PAP | 5.340 | 0.880 | -183888 | 367780 |
| CEN_SYM | 5.305 | 0.719 | -12680 | 25364 |
| CHL_MUR | 5.600 | 0.714 | -6047 | 12098 |
| CHL_REI | 5.699 | 0.825 | -107808 | 215620 |
| COR_AUR | 5.575 | 0.650 | -16546 | 33096 |
| CRY_PAR | 5.977 | 0.683 | -25192 | 50389 |
| CYA_MER | 5.943 | 0.661 | -33924 | 67852 |
| DAN_RER | 5.855 | 0.660 | -183759 | 367522 |
| DEI_DES | 5.560 | 0.608 | -22271 | 44546 |
| DES_KAM | 5.362 | 0.741 | -9527 | 19058 |
| DIC_DIS | 5.810 | 0.870 | -87894 | 175792 |
| DRO_MEL | 5.933 | 0.725 | -121034 | 242071 |
| EQU_CAB | 5.919 | 0.699 | -148809 | 297622 |
| ESC_COL | 5.347 | 0.816 | -36909 | 73823 |
| GAL_GAL | 5.745 | 0.801 | -145888 | 291780 |
| GLO_VIO | 5.505 | 0.684 | -28776 | 57556 |
| GLY_MAX | 5.810 | 0.642 | -310026 | 620056 |
| GUI_LAM | 5.724 | 0.907 | -38058 | 76120 |
| HOM_SAP | 5.642 | 0.843 | -419212 | 838429 |
| HYD_THE | 5.468 | 0.666 | -12242 | 24489 |
| KOC_RHI | 5.627 | 0.640 | -15503 | 31010 |
| LAC_BIC | 5.590 | 0.779 | -135335 | 270674 |
| LEP_BIF | 5.635 | 0.605 | -23500 | 47004 |
| MED_TRU | 5.114 | 0.844 | -324818 | 649640 |
| MEL_GAL | 5.772 | 0.763 | -115057 | 230118 |
| MET_JAN | 5.441 | 0.626 | -11280 | 22564 |
| MIC_CCM | 5.752 | 0.683 | -69769 | 139543 |
| MIC_RCC | 5.861 | 0.660 | -67178 | 134361 |
| MON_DOM | 6.026 | 0.669 | -218888 | 437781 |
| MYC_ABS | 5.560 | 0.600 | -31829 | 63662 |
| NAN_EQU | 5.402 | 0.645 | -3542 | 7088 |
| NEM_VEC | 5.491 | 0.712 | -173146 | 346296 |
| ORN_ANA | 5.756 | 0.710 | -178259 | 356522 |
| ORY_SAT | 5.725 | 0.768 | -421372 | 842747 |
| OST_LUC | 5.707 | 0.671 | -49417 | 98838 |
| OST_TAU | 5.671 | 0.684 | -51128 | 102260 |
| PAR_TET | 5.768 | 0.690 | -259277 | 518559 |
| PER_MAR | 5.486 | 0.681 | -13337 | 26677 |
| PHA_CHR | 5.894 | 0.616 | -67361 | 134727 |
| PHA_TRI | 5.898 | 0.611 | -66939 | 133883 |
| PHY_PAT | 5.615 | 0.689 | -234850 | 469704 |
| PHY_RAM | 5.886 | 0.643 | -103792 | 207588 |
| PHY_SOJ | 5.885 | 0.693 | -126215 | 252435 |
| PIC_STI | 5.958 | 0.627 | -39474 | 78952 |
| PLA_FAL | 5.953 | 0.770 | -33604 | 67212 |
| POP_TRI | 5.648 | 0.727 | -297180 | 594364 |
| PRI_PAC | 5.330 | 0.751 | -189343 | 378691 |
| RAT_NOR | 5.854 | 0.744 | -219205 | 438414 |
| SAC_CER | 5.896 | 0.728 | -39601 | 79206 |
| SEL_MOE | 5.701 | 0.657 | -147053 | 294109 |
| SOR_BIC | 5.615 | 0.720 | -236983 | 473969 |
| STA_AUR | 5.442 | 0.715 | -17127 | 34259 |
| STR_AVE | 5.605 | 0.628 | -50028 | 100059 |
| SUL_ACI | 5.453 | 0.646 | -14705 | 29415 |
| SUL_DEL | 5.531 | 0.655 | -14783 | 29570 |
| SYN_SP | 5.512 | 0.688 | -21118 | 42240 |
| TAK_RUB | 6.134 | 0.602 | -318355 | 636714 |
| TET_THE | 5.903 | 0.866 | -162339 | 324682 |
| THA_PSE | 5.917 | 0.648 | -76148 | 152300 |
| THE_ELO | 5.526 | 0.685 | -16086 | 32176 |
| THE_NEU | 5.388 | 0.643 | -12758 | 25519 |
| THE_THE | 5.522 | 0.620 | -14186 | 28377 |
| THE_VOL | 5.492 | 0.649 | -10085 | 20175 |
| TOX_GON | 5.935 | 0.737 | -51467 | 102939 |
| TRI_ADH | 5.812 | 0.644 | -76306 | 152616 |
| TRI_REE | 5.930 | 0.645 | -61698 | 123401 |
| UST_MAY | 6.102 | 0.666 | -44151 | 88305 |
| XAN_CAM | 5.418 | 0.834 | -29933 | 59871 |
| ZEA_MAY | 5.580 | 0.735 | -325559 | 651122 |

Table S4 – Protein length amino acid bias

The pearson correlation coefficients R of percentage amino acid composition with protein length were calculated for each species individually. Positive or negative R values indicate the direction of the correlation.

| **Group** | **Species** | **%A** | **%C** | **%D** | **%E** | **%F** | **%G** | **%H** | **%I** | **%K** | **%L** |
| --- | --- | --- | --- | --- | --- | --- | --- | --- | --- | --- | --- |
| ARCHAEA | ARC_PRO | 0.15 | -0.10 | 0.09 | -0.08 | 0.00 | 0.10 | 0.07 | -0.04 | -0.21 | -0.05 |
| ARCHAEA | MET_JAN | 0.00 | -0.10 | 0.14 | -0.01 | 0.00 | 0.09 | 0.05 | -0.10 | -0.11 | -0.03 |
| BACTERIA | BAC_SUB | 0.13 | -0.10 | 0.04 | -0.04 | -0.03 | 0.10 | -0.01 | -0.04 | -0.13 | 0.01 |
| BACTERIA | ESC_COL | 0.12 | -0.19 | 0.10 | -0.01 | -0.10 | 0.20 | -0.06 | -0.08 | -0.17 | 0.03 |
| BACTERIA | GLO_VIO | 0.07 | -0.13 | 0.06 | -0.10 | 0.05 | 0.16 | -0.05 | -0.05 | -0.13 | 0.01 |
| BACTERIA | SYN_SP | 0.06 | -0.13 | 0.08 | -0.05 | -0.03 | 0.15 | -0.02 | -0.01 | -0.16 | -0.01 |
| BACTERIA | THE_ELO | 0.05 | -0.11 | 0.12 | 0.02 | -0.03 | 0.10 | 0.02 | 0.02 | -0.09 | 0.01 |
| INSECTA | DRO_MEL | -0.01 | -0.09 | 0.05 | 0.10 | -0.14 | -0.04 | 0.02 | -0.08 | -0.04 | -0.04 |
| VERT_AVE | GAL_GAL | -0.12 | -0.10 | 0.16 | 0.13 | -0.06 | -0.07 | -0.04 | 0.06 | 0.05 | -0.03 |
| VERT_AVE | MEL_GAL | -0.07 | -0.09 | 0.11 | 0.09 | -0.09 | -0.05 | -0.03 | 0.01 | 0.02 | -0.03 |
| VERT_MAM | BOS_TAU | 0.01 | -0.07 | 0.11 | 0.12 | -0.13 | 0.01 | 0.00 | -0.07 | -0.01 | -0.07 |
| VERT_MAM | EQU_CAB | -0.03 | -0.05 | 0.09 | 0.06 | -0.10 | 0.02 | 0.02 | -0.06 | -0.03 | -0.04 |
| VERT_MAM | HOM_SAP | -0.04 | -0.07 | 0.08 | 0.07 | -0.05 | -0.04 | 0.00 | 0.02 | 0.02 | -0.04 |
| VERT_MAM | MON_DOM | -0.06 | -0.03 | 0.07 | 0.13 | -0.13 | -0.01 | 0.04 | -0.06 | 0.03 | -0.08 |
| VERT_MAM | ORN_ANA | -0.02 | -0.06 | 0.08 | 0.11 | -0.09 | -0.01 | 0.00 | -0.04 | 0.01 | -0.06 |
| VERT_MAM | RAT_NOR | 0.00 | -0.09 | 0.12 | 0.10 | -0.10 | -0.02 | 0.00 | -0.05 | -0.05 | -0.01 |
| VERT_SAU | ANO_CAR | -0.04 | -0.05 | 0.07 | 0.01 | -0.10 | -0.04 | 0.01 | 0.00 | -0.05 | -0.01 |
| VERT_TEL | DAN_RER | -0.01 | -0.06 | 0.04 | 0.05 | -0.09 | 0.00 | 0.00 | -0.05 | -0.05 | 0.00 |
| VERT_TEL | TAK_RUB | -0.05 | -0.06 | 0.07 | 0.08 | -0.13 | 0.05 | -0.01 | -0.05 | -0.04 | -0.05 |
| FUNGI_ASC | PIC_STI | -0.14 | -0.06 | 0.05 | 0.00 | 0.04 | -0.12 | -0.01 | 0.10 | -0.10 | 0.10 |
| FUNGI_ASC | SAC_CER | -0.12 | -0.12 | 0.13 | 0.05 | -0.02 | -0.12 | -0.01 | 0.08 | -0.08 | 0.04 |
| FUNGI_ASC | TRI_REE | -0.05 | -0.09 | 0.09 | 0.05 | -0.02 | -0.08 | -0.01 | 0.01 | -0.06 | 0.04 |
| FUNGI_BAS | PHA_CHR | -0.01 | -0.13 | 0.05 | 0.07 | -0.04 | -0.09 | -0.02 | -0.06 | -0.07 | 0.03 |
| STRAM_DIA | PHA_TRI | -0.01 | -0.05 | 0.04 | 0.02 | -0.07 | -0.10 | 0.04 | -0.05 | -0.10 | 0.05 |
| STRAM_DIA | THA_PSE | 0.00 | -0.06 | 0.07 | 0.01 | -0.09 | -0.05 | -0.01 | -0.09 | -0.08 | -0.03 |
| STRAM_OOM | PHY_RAM | 0.00 | -0.06 | 0.00 | 0.04 | 0.00 | -0.04 | -0.02 | 0.01 | -0.09 | 0.08 |
| STRAM_OOM | PHY_SOJ | -0.02 | -0.09 | 0.03 | 0.07 | 0.01 | -0.06 | -0.04 | 0.01 | -0.06 | 0.06 |
| PLANT_BRY | PHY_PAT | 0.07 | -0.11 | 0.10 | 0.08 | -0.04 | 0.02 | -0.08 | -0.07 | 0.02 | 0.06 |
| PLANT_CHL | CHL_REI | 0.29 | -0.17 | 0.00 | 0.00 | -0.16 | 0.20 | -0.08 | -0.17 | -0.20 | -0.03 |
| PLANT_CHL | MIC_CCM | 0.17 | -0.10 | 0.09 | 0.07 | -0.08 | 0.01 | -0.06 | -0.05 | -0.07 | 0.02 |
| PLANT_CHL | MIC_RCC | 0.15 | -0.08 | 0.09 | 0.06 | -0.09 | 0.01 | -0.04 | -0.09 | -0.12 | 0.01 |
| PLANT_CHL | OST_LUC | -0.07 | -0.07 | 0.04 | 0.01 | -0.04 | -0.10 | 0.04 | 0.12 | -0.06 | 0.11 |
| PLANT_CHL | OST_TAU | -0.06 | -0.04 | 0.05 | 0.07 | -0.01 | -0.07 | -0.01 | 0.12 | 0.01 | 0.14 |
| PLANT_DIC | ARA_THA | 0.01 | -0.13 | 0.11 | 0.10 | -0.07 | -0.03 | 0.00 | -0.01 | -0.07 | 0.12 |
| PLANT_DIC | CAR_PAP | -0.01 | -0.12 | 0.15 | 0.02 | 0.11 | -0.05 | -0.06 | 0.10 | -0.05 | 0.05 |
| PLANT_DIC | GLY_MAX | -0.03 | -0.09 | 0.09 | 0.05 | -0.06 | 0.00 | -0.01 | 0.03 | -0.08 | 0.10 |
| PLANT_DIC | MED_TRU | 0.13 | -0.14 | 0.15 | 0.12 | -0.12 | 0.07 | -0.08 | -0.06 | -0.02 | 0.03 |
| PLANT_DIC | POP_TRI | -0.02 | -0.12 | 0.13 | 0.10 | -0.08 | 0.00 | -0.03 | -0.01 | -0.06 | 0.08 |
| PLANT_MON | BRA_DIS | -0.22 | -0.08 | 0.08 | 0.05 | 0.03 | -0.13 | 0.05 | 0.16 | 0.02 | 0.14 |
| PLANT_MON | ORY_SAT | -0.21 | -0.10 | 0.13 | 0.12 | 0.14 | -0.22 | 0.06 | 0.16 | 0.18 | 0.08 |
| PLANT_MON | SOR_BIC | -0.09 | -0.09 | 0.08 | 0.04 | 0.05 | -0.06 | -0.04 | 0.05 | -0.02 | 0.17 |
| PLANT_MON | ZEA_MAY | -0.10 | -0.16 | 0.17 | 0.13 | -0.03 | -0.03 | 0.00 | 0.08 | 0.04 | 0.03 |
| PLANT_LYC | SEL_MOE | 0.04 | -0.13 | 0.04 | 0.09 | -0.02 | -0.04 | -0.05 | -0.03 | -0.06 | 0.08 |
|  |  |  |  |  |  |  |  |  |  |  |  |
|  |  |  |  |  |  |  |  |  |  |  |  |
| **Group** | **Species** | **%M** | **%N** | **%P** | **%Q** | **%R** | **%S** | **%T** | **%V** | **%W** | **%Y** |
| ARCHAEA | ARC_PRO | -0.20 | 0.08 | 0.17 | 0.03 | -0.06 | 0.06 | 0.17 | -0.03 | -0.02 | 0.07 |
| ARCHAEA | MET_JAN | -0.24 | 0.11 | 0.08 | 0.00 | -0.05 | 0.05 | 0.11 | -0.05 | 0.06 | 0.11 |
| BACTERIA | BAC_SUB | -0.17 | -0.04 | 0.14 | -0.01 | -0.02 | 0.06 | 0.07 | 0.01 | 0.01 | -0.03 |
| BACTERIA | ESC_COL | -0.24 | 0.01 | 0.07 | 0.06 | -0.05 | -0.01 | 0.07 | 0.03 | 0.02 | 0.00 |
| BACTERIA | GLO_VIO | -0.28 | 0.09 | 0.05 | -0.05 | -0.08 | 0.04 | 0.09 | 0.02 | -0.04 | 0.03 |
| BACTERIA | SYN_SP | -0.26 | 0.11 | 0.05 | 0.06 | -0.09 | 0.01 | 0.10 | 0.01 | -0.06 | 0.00 |
| BACTERIA | THE_ELO | -0.28 | 0.04 | 0.00 | 0.09 | -0.02 | -0.06 | 0.05 | -0.05 | -0.06 | -0.01 |
| INSECTA | DRO_MEL | -0.13 | 0.05 | 0.07 | 0.13 | -0.05 | 0.15 | 0.09 | -0.06 | -0.09 | -0.13 |
| VERT_AVE | GAL_GAL | -0.03 | 0.16 | -0.04 | 0.09 | -0.13 | 0.06 | 0.04 | -0.01 | -0.12 | -0.03 |
| VERT_AVE | MEL_GAL | -0.02 | 0.11 | 0.00 | 0.08 | -0.08 | 0.09 | 0.05 | -0.02 | -0.08 | -0.05 |
| VERT_MAM | BOS_TAU | -0.14 | 0.02 | 0.08 | 0.09 | -0.01 | 0.10 | 0.02 | -0.05 | -0.06 | -0.11 |
| VERT_MAM | EQU_CAB | -0.10 | 0.02 | 0.06 | 0.09 | -0.06 | 0.11 | 0.03 | -0.01 | -0.05 | -0.08 |
| VERT_MAM | HOM_SAP | -0.12 | 0.07 | 0.03 | 0.06 | -0.06 | 0.07 | 0.06 | 0.00 | -0.10 | -0.03 |
| VERT_MAM | MON_DOM | -0.13 | 0.05 | 0.05 | 0.11 | -0.02 | 0.07 | 0.05 | -0.06 | -0.09 | -0.10 |
| VERT_MAM | ORN_ANA | -0.07 | 0.05 | 0.04 | 0.08 | -0.05 | 0.07 | 0.03 | -0.04 | -0.08 | -0.08 |
| VERT_MAM | RAT_NOR | -0.13 | 0.03 | 0.08 | 0.08 | -0.04 | 0.09 | 0.01 | -0.02 | -0.09 | -0.10 |
| VERT_SAU | ANO_CAR | -0.04 | 0.07 | 0.07 | 0.08 | -0.07 | 0.14 | 0.06 | -0.02 | -0.09 | -0.08 |
| VERT_TEL | DAN_RER | -0.10 | 0.01 | 0.09 | 0.07 | -0.03 | 0.06 | 0.03 | -0.01 | -0.05 | -0.07 |
| VERT_TEL | TAK_RUB | -0.09 | 0.05 | 0.10 | 0.10 | -0.04 | 0.09 | 0.05 | -0.05 | -0.09 | -0.11 |
| FUNGI_ASC | PIC_STI | -0.05 | 0.15 | -0.01 | -0.01 | -0.06 | 0.18 | 0.00 | -0.09 | -0.03 | -0.04 |
| FUNGI_ASC | SAC_CER | -0.16 | 0.19 | 0.03 | -0.02 | -0.12 | 0.16 | 0.07 | -0.13 | -0.04 | -0.04 |
| FUNGI_ASC | TRI_REE | -0.04 | 0.01 | 0.04 | 0.07 | 0.01 | 0.07 | -0.01 | -0.03 | -0.04 | -0.06 |
| FUNGI_BAS | PHA_CHR | -0.04 | -0.06 | 0.09 | 0.08 | 0.06 | 0.14 | -0.03 | -0.06 | -0.06 | -0.10 |
| STRAM_DIA | PHA_TRI | -0.14 | 0.04 | 0.04 | 0.00 | 0.05 | 0.19 | 0.06 | -0.06 | -0.02 | -0.10 |
| STRAM_DIA | THA_PSE | -0.11 | 0.10 | 0.06 | 0.07 | -0.01 | 0.22 | 0.05 | -0.08 | -0.08 | -0.10 |
| STRAM_OOM | PHY_RAM | -0.09 | 0.02 | 0.02 | 0.03 | 0.02 | 0.08 | -0.02 | -0.03 | -0.04 | -0.05 |
| STRAM_OOM | PHY_SOJ | -0.08 | 0.00 | 0.02 | 0.02 | -0.01 | 0.07 | 0.00 | 0.00 | -0.03 | -0.04 |
| PLANT_BRY | PHY_PAT | -0.08 | -0.02 | -0.02 | -0.04 | -0.10 | 0.07 | 0.00 | 0.03 | -0.09 | -0.03 |
| PLANT_CHL | CHL_REI | -0.14 | -0.09 | 0.06 | 0.07 | -0.11 | 0.11 | -0.05 | -0.11 | -0.10 | -0.12 |
| PLANT_CHL | MIC_CCM | -0.12 | -0.04 | 0.01 | -0.02 | -0.05 | 0.02 | 0.03 | -0.06 | -0.05 | -0.10 |
| PLANT_CHL | MIC_RCC | -0.15 | -0.04 | 0.05 | -0.03 | 0.02 | 0.07 | 0.00 | -0.04 | -0.03 | -0.09 |
| PLANT_CHL | OST_LUC | -0.04 | 0.05 | 0.04 | 0.14 | -0.13 | 0.17 | 0.05 | -0.03 | -0.03 | -0.05 |
| PLANT_CHL | OST_TAU | -0.03 | 0.08 | -0.04 | 0.14 | -0.18 | 0.04 | -0.01 | -0.01 | -0.01 | 0.01 |
| PLANT_DIC | ARA_THA | -0.18 | 0.05 | -0.03 | 0.08 | -0.05 | 0.05 | -0.05 | -0.02 | 0.00 | -0.02 |
| PLANT_DIC | CAR_PAP | -0.15 | 0.11 | -0.01 | 0.01 | -0.17 | 0.11 | -0.05 | 0.01 | -0.08 | 0.06 |
| PLANT_DIC | GLY_MAX | -0.08 | 0.03 | -0.04 | 0.05 | -0.02 | 0.04 | -0.04 | -0.03 | 0.01 | -0.03 |
| PLANT_DIC | MED_TRU | -0.25 | 0.03 | 0.03 | 0.03 | -0.09 | 0.06 | -0.04 | -0.02 | -0.07 | -0.02 |
| PLANT_DIC | POP_TRI | -0.15 | 0.07 | -0.04 | 0.04 | -0.05 | 0.06 | -0.05 | 0.00 | -0.04 | -0.04 |
| PLANT_MON | BRA_DIS | -0.11 | 0.22 | -0.08 | 0.07 | -0.11 | 0.15 | 0.05 | -0.04 | -0.02 | 0.03 |
| PLANT_MON | ORY_SAT | -0.15 | 0.20 | -0.02 | 0.16 | -0.14 | -0.05 | 0.02 | -0.02 | -0.05 | 0.15 |
| PLANT_MON | SOR_BIC | -0.04 | 0.09 | -0.07 | 0.01 | -0.17 | 0.17 | -0.02 | 0.01 | -0.05 | 0.01 |
| PLANT_MON | ZEA_MAY | -0.13 | 0.17 | -0.07 | 0.06 | -0.12 | 0.04 | 0.00 | 0.00 | -0.05 | 0.01 |
| PLANT_LYC | SEL_MOE | -0.08 | -0.04 | 0.00 | 0.05 | -0.01 | 0.06 | 0.01 | 0.02 | -0.05 | -0.06 |

Table S5 – Pararameter estimates for the Pareto’s model and Chi-squared goodness of fit.

| **Specie** | $\hat{\alpha}$ | ***p-value*** | **Specie** | $\hat{\alpha}$ | ***p-value*** |
| --- | --- | --- | --- | --- | --- |
| ANO_CAR | 2.9011 | 0.5991 | NEM_VEC | 2.7748 | 0.5141 |
| ANO_GAM | 2.6090 | 0.0002 | ORN_ANA | 2.9600 | 0.0371 |
| ARA_THA | 3.8303 | 0.0041 | ORY_SAT | 5.6232 | 0.0000 |
| BAC_SUB | 1.5109 | 0.0119 | OST_LUC | 2.7364 | 0.1806 |
| BOS_TAU | 2.7355 | 0.0194 | OST_TAU | 2.6773 | 0.9072 |
| BRA_DIS | 3.8769 | 0.0547 | PAR_TET | 2.6396 | 0.0000 |
| BRA_JAP | 2.2098 | 0.2548 | PHA_CHR | 3.8830 | 0.2679 |
| BUR_CEP | 2.1409 | 0.4244 | PHA_TRI | 3.5987 | 0.0859 |
| CAE_ELE | 2.5934 | 0.3335 | PHY_PAT | 3.2467 | 0.0102 |
| CAR_PAP | 5.8876 | 0.0225 | PHY_RAM | 3.0660 | 0.2485 |
| CEN_SYM | 1.5507 | 0.8077 | PHY_SOJ | 3.0399 | 0.0010 |
| CHL_REI | 2.5631 | 0.0029 | PIC_STI | 4.2626 | 0.1972 |
| CRY_PAR | 2.6480 | 0.3307 | PLA_FAL | 2.1978 | 0.0000 |
| CYA_MER | 3.1345 | 0.3011 | POP_TRI | 4.2405 | 0.0070 |
| DAN_RER | 2.9546 | 0.2196 | PRI_PAC | 3.2766 | 0.0673 |
| DIC_DIS | 3.1082 | 0.1668 | RAT_NOR | 2.8880 | 0.0002 |
| DRO_MEL | 2.6523 | 0.0000 | SAC_CER | 4.7499 | 0.0000 |
| EQU_CAB | 2.6242 | 0.0000 | SEL_MOE | 3.3190 | 0.0370 |
| ESC_COL | 3.4686 | 0.0594 | SOR_BIC | 4.3144 | 0.1005 |
| GAL_GAL | 2.6925 | 0.0000 | STR_AVE | 1.5728 | 0.0138 |
| GLO_VIO | 4.8272 | 0.5259 | SYN_SP | 3.3293 | 0.0119 |
| GLY_MAX | 3.1671 | 0.0546 | TAK_RUB | 3.0497 | 0.0000 |
| GUI_LAM | 2.5210 | 0.0325 | TET_THE | 2.8058 | 0.0000 |
| HOM_SAP | 2.6760 | 0.0000 | THA_PSE | 3.2251 | 0.0212 |
| LAC_BIC | 4.3408 | 0.1742 | TOX_GON | 2.3896 | 0.0211 |
| MED_TRU | 4.6428 | 0.3335 | TRI_ADH | 2.6738 | 0.6294 |
| MEL_GAL | 3.0131 | 0.1374 | TRI_REE | 3.5987 | 0.9483 |
| MIC_CCM | 2.6258 | 0.6164 | UST_MAY | 4.2862 | 0.2985 |
| MIC_RCC | 2.6551 | 0.4178 | XAN_CAM | 2.5751 | 0.6718 |
| MON_DOM | 3.0237 | 0.0000 | ZEA_MAY | 4.3303 | 0.0951 |
| MYC_ABS | 2.5673 | 0.6028 |  |  |  |

Table S6 – Taxonomic classification, KEGG code, species name, number of proteins and gene-based G+C content for each species of dataset 2.

| SuperKingdom | Kingdom | Group | KEGG code | Species | Number of proteins | G+C content (gene based) |
| --- | --- | --- | --- | --- | --- | --- |
| Prokaryotes | Bacteria | Betaproteobacteria | aaa | Acidovorax avenae subsp. avenae ATCC 19860 | 4737 | 0.692 |
| Prokaryotes | Bacteria | Firmicutes | aac | Alicyclobacillus acidocaldarius | 3084 | 0.624 |
| Prokaryotes | Bacteria | Hyperthermophilic bacteria | aae | Aquifex aeolicus | 1560 | 0.438 |
| Eukaryotes | Animals | Arthropods | aag | Aedes aegypti (yellow fever mosquito) | 15428 | 0.499 |
| Prokaryotes | Bacteria | Actinobacteria | aai | Arthrobacter arilaitensis | 3436 | 0.602 |
| Prokaryotes | Bacteria | Gammaproteobacteria | aap | Aggregatibacter aphrophilus | 2219 | 0.433 |
| Prokaryotes | Bacteria | Firmicutes | aar | Acetohalobium arabaticum | 2282 | 0.375 |
| Prokaryotes | Bacteria | Bacteroidetes | aas | Candidatus Amoebophilus asiaticus | 1334 | 0.362 |
| Prokaryotes | Bacteria | Gammaproteobacteria | aat | Aggregatibacter actinomycetemcomitans | 2219 | 0.455 |
| Prokaryotes | Bacteria | Actinobacteria | aau | Arthrobacter aurescens | 4587 | 0.628 |
| Prokaryotes | Bacteria | Betaproteobacteria | aav | Acidovorax avenae | 4709 | 0.690 |
| Prokaryotes | Bacteria | Acidobacteria | aba | Candidatus Koribacter versatilis | 4777 | 0.589 |
| Prokaryotes | Bacteria | Gammaproteobacteria | abb | Acinetobacter baumannii AB307-0294 | 3451 | 0.403 |
| Prokaryotes | Bacteria | Gammaproteobacteria | abc | Acinetobacter baumannii ACICU | 3759 | 0.401 |
| Prokaryotes | Archaea | Euryarchaeota | abi | Aciduliprofundum boonei | 1544 | 0.395 |
| Prokaryotes | Bacteria | Gammaproteobacteria | abm | Acinetobacter baumannii SDF | 2975 | 0.401 |
| Prokaryotes | Bacteria | Gammaproteobacteria | abn | Acinetobacter baumannii AB0057 | 3801 | 0.404 |
| Prokaryotes | Bacteria | Gammaproteobacteria | abo | Alcanivorax borkumensis | 2755 | 0.552 |
| Prokaryotes | Bacteria | Epsilonproteobacteria | abu | Arcobacter butzleri | 2259 | 0.274 |
| Prokaryotes | Bacteria | Gammaproteobacteria | aby | Acinetobacter baumannii AYE | 3712 | 0.404 |
| Prokaryotes | Bacteria | Acidobacteria | aca | Acidobacterium capsulatum | 3377 | 0.611 |
| Prokaryotes | Bacteria | Gammaproteobacteria | acb | Acinetobacter baumannii ATCC 17978 | 3367 | 0.405 |
| Prokaryotes | Bacteria | Gammaproteobacteria | acd | Acinetobacter sp. DR1 | 3874 | 0.399 |
| Prokaryotes | Bacteria | Actinobacteria | ace | Acidothermus cellulolyticus | 2157 | 0.667 |
| Prokaryotes | Bacteria | Actinobacteria | ach | Arthrobacter chlorophenolicus | 4590 | 0.664 |
| Prokaryotes | Bacteria | Gammaproteobacteria | aci | Acinetobacter sp. ADP1 | 3307 | 0.416 |
| Prokaryotes | Bacteria | Tenericutes | acl | Acholeplasma laidlawii | 1380 | 0.324 |
| Prokaryotes | Bacteria | Acidobacteria | acm | Acidobacterium sp. MP5ACTX9 | 4542 | 0.608 |
| Prokaryotes | Bacteria | Alphaproteobacteria | acn | Anaplasma centrale | 923 | 0.500 |
| Prokaryotes | Bacteria | Synergistetes | aco | Aminobacterium colombiense | 1876 | 0.458 |
| Prokaryotes | Bacteria | Deltaproteobacteria | acp | Anaeromyxobacter dehalogenans 2CP-1 | 4473 | 0.746 |
| Prokaryotes | Bacteria | Alphaproteobacteria | acr | Acidiphilium cryptum JF-5 | 3559 | 0.678 |
| Eukaryotes | Fungi | Ascomycetes | act | Aspergillus clavatus | 9121 | 0.553 |
| Prokaryotes | Bacteria | Deltaproteobacteria | ade | Anaeromyxobacter dehalogenans 2CP-C | 4346 | 0.748 |
| Prokaryotes | Bacteria | Firmicutes | adg | Ammonifex degensii | 2080 | 0.599 |
| Prokaryotes | Bacteria | Betaproteobacteria | adk | Alicycliphilus denitrificans K601 | 4696 | 0.685 |
| Prokaryotes | Bacteria | Betaproteobacteria | adn | Alicycliphilus denitrificans BC | 4542 | 0.686 |
| Prokaryotes | Bacteria | Gammaproteobacteria | aeh | Alkalilimnicola ehrlichei | 2865 | 0.678 |
| Prokaryotes | Bacteria | Alphaproteobacteria | aex | Asticcacaulis excentricus | 3763 | 0.603 |
| Prokaryotes | Bacteria | Gammaproteobacteria | afe | Acidithiobacillus ferrooxidans ATCC 53993 | 2826 | 0.595 |
| Prokaryotes | Bacteria | Firmicutes | afl | Anoxybacillus flavithermus | 2831 | 0.423 |
| Eukaryotes | Fungi | Ascomycetes | afm | Aspergillus fumigatus | 9630 | 0.540 |
| Prokaryotes | Bacteria | Firmicutes | afn | Acidaminococcus fermentans | 2026 | 0.570 |
| Prokaryotes | Bacteria | Actinobacteria | afo | Acidimicrobium ferrooxidans DSM 10331 | 1964 | 0.683 |
| Prokaryotes | Bacteria | Gammaproteobacteria | afr | Acidithiobacillus ferrooxidans ATCC 23270 | 3147 | 0.593 |
| Prokaryotes | Archaea | Euryarchaeota | afu | Archaeoglobus fulgidus | 2420 | 0.494 |
| Eukaryotes | Fungi | Ascomycetes | afv | Aspergillus flavus | 13485 | 0.521 |
| Prokaryotes | Bacteria | Deltaproteobacteria | afw | Anaeromyxobacter sp. Fw109-5 | 4466 | 0.735 |
| Eukaryotes | Animals | Arthropods | aga | Anopheles gambiae (mosquito) | 12460 | 0.564 |
| Eukaryotes | Fungi | Ascomycetes | ago | Ashbya gossypii (Eremothecium gossypii) | 4776 | 0.526 |
| Prokaryotes | Bacteria | Alphaproteobacteria | agr | Agrobacterium sp. H13-3 | 3403 | 0.594 |
| Prokaryotes | Bacteria | Gammaproteobacteria | aha | Aeromonas hydrophila | 4121 | 0.627 |
| Prokaryotes | Bacteria | Actinobacteria | ahe | Arcanobacterium haemolyticum | 1731 | 0.537 |
| Prokaryotes | Bacteria | Betaproteobacteria | ajs | Acidovorax sp. JS42 | 4155 | 0.666 |
| Prokaryotes | Bacteria | Gammaproteobacteria | alv | Allochromatium vinosum | 3220 | 0.647 |
| Prokaryotes | Bacteria | Alphaproteobacteria | ama | Anaplasma marginale St. Maries | 948 | 0.498 |
| Prokaryotes | Bacteria | Gammaproteobacteria | amc | Alteromonas macleodii | 4072 | 0.457 |
| Prokaryotes | Bacteria | Actinobacteria | amd | Amycolatopsis mediterranei | 9228 | 0.718 |
| Eukaryotes | Animals | Arthropods | ame | Apis mellifera (honey bee) | 9102 | 0.399 |
| Prokaryotes | Bacteria | Alphaproteobacteria | amf | Anaplasma marginale Florida | 940 | 0.498 |
| Prokaryotes | Bacteria | Actinobacteria | ami | Actinosynnema mirum | 6916 | 0.740 |
| Eukaryotes | Animals | Vertebrates | aml | Ailuropoda melanoleuca (giant panda) | 17905 | 0.530 |
| Prokaryotes | Bacteria | Cyanobacteria | amr | Acaryochloris marina | 8383 | 0.481 |
| Prokaryotes | Bacteria | Firmicutes | amt | Alkaliphilus metalliredigens | 4625 | 0.376 |
| Prokaryotes | Bacteria | Verrucomicrobia | amu | Akkermansia muciniphila | 2138 | 0.567 |
| Prokaryotes | Bacteria | Alphaproteobacteria | amv | Acidiphilium multivorum | 3948 | 0.677 |
| Prokaryotes | Bacteria | Cyanobacteria | ana | Anabaena sp. PCC7120 | 6129 | 0.424 |
| Eukaryotes | Fungi | Ascomycetes | ang | Aspergillus niger | 10505 | 0.542 |
| Eukaryotes | Fungi | Ascomycetes | ani | Aspergillus nidulans | 9541 | 0.534 |
| Prokaryotes | Bacteria | Deltaproteobacteria | ank | Anaeromyxobacter sp. K | 4457 | 0.747 |
| Prokaryotes | Bacteria | Epsilonproteobacteria | ant | Arcobacter nitrofigilis | 3126 | 0.288 |
| Prokaryotes | Bacteria | Firmicutes | aoe | Alkaliphilus oremlandii | 2836 | 0.373 |
| Eukaryotes | Fungi | Ascomycetes | aor | Aspergillus oryzae | 12691 | 0.521 |
| Prokaryotes | Bacteria | Gammaproteobacteria | apa | Actinobacillus pleuropneumoniae AP76 (serotype 7) | 2142 | 0.423 |
| Prokaryotes | Bacteria | Alphaproteobacteria | apb | Candidatus Puniceispirillum marinum | 2543 | 0.496 |
| Prokaryotes | Archaea | Crenarchaeota | ape | Aeropyrum pernix | 1700 | 0.571 |
| Prokaryotes | Bacteria | Alphaproteobacteria | aph | Anaplasma phagocytophilum | 1264 | 0.427 |
| Eukaryotes | Animals | Arthropods | api | Acyrthosiphon pisum (pea aphid) | 10363 | 0.393 |
| Prokaryotes | Bacteria | Gammaproteobacteria | apj | Actinobacillus pleuropneumoniae JL03 (serotype 3) | 2036 | 0.423 |
| Prokaryotes | Bacteria | Gammaproteobacteria | apl | Actinobacillus pleuropneumoniae L20 (serotype 5b) | 2012 | 0.425 |
| Prokaryotes | Bacteria | Actinobacteria | apn | Arthrobacter phenanthrenivorans | 4131 | 0.658 |
| Prokaryotes | Archaea | Euryarchaeota | apo | Archaeoglobus profundus | 1823 | 0.424 |
| Prokaryotes | Bacteria | Betaproteobacteria | app | Accumulibacter phosphatis | 4562 | 0.641 |
| Prokaryotes | Bacteria | Firmicutes | apr | Anaerococcus prevotii | 1806 | 0.362 |
| Prokaryotes | Bacteria | Bacteroidetes | aps | Candidatus Azobacteroides pseudotrichonymphae | 852 | 0.342 |
| Prokaryotes | Bacteria | Alphaproteobacteria | apt | Acetobacter pasteurianus | 3050 | 0.539 |
| Prokaryotes | Bacteria | Actinobacteria | apv | Atopobium parvulum | 1353 | 0.464 |
| Prokaryotes | Bacteria | Alphaproteobacteria | ara | Agrobacterium radiobacter K84 | 6684 | 0.608 |
| Prokaryotes | Bacteria | Actinobacteria | art | Arthrobacter sp. FB24 | 4523 | 0.658 |
| Prokaryotes | Bacteria | Gammaproteobacteria | asa | Aeromonas salmonicida | 4436 | 0.592 |
| Prokaryotes | Archaea | Crenarchaeota | asc | Acidilobus saccharovorans | 1499 | 0.580 |
| Prokaryotes | Bacteria | Gammaproteobacteria | asu | Actinobacillus succinogenes | 2079 | 0.460 |
| Prokaryotes | Bacteria | Firmicutes | ate | Caldicellulosiruptor bescii | 2666 | 0.357 |
| Eukaryotes | Plants | Eudicots | ath | Arabidopsis thaliana (thale cress) | 27361 | 0.441 |
| Prokaryotes | Bacteria | Green nonsulfur bacteria | atm | Anaerolinea thermophila | 3166 | 0.544 |
| Prokaryotes | Bacteria | Alphaproteobacteria | atu | Agrobacterium tumefaciens C58 | 5355 | 0.598 |
| Prokaryotes | Bacteria | Firmicutes | aur | Aerococcus urinae | 1726 | 0.429 |
| Prokaryotes | Bacteria | Cyanobacteria | ava | Anabaena variabilis | 5710 | 0.425 |
| Prokaryotes | Archaea | Euryarchaeota | ave | Archaeoglobus veneficus | 2090 | 0.479 |
| Prokaryotes | Bacteria | Alphaproteobacteria | avi | Agrobacterium vitis S4 | 5389 | 0.585 |
| Prokaryotes | Bacteria | Gammaproteobacteria | avn | Azotobacter vinelandii | 5050 | 0.663 |
| Prokaryotes | Bacteria | Gammaproteobacteria | avr | Aeromonas veronii | 4028 | 0.600 |
| Prokaryotes | Bacteria | Betaproteobacteria | axy | Achromobacter xylosoxidans | 6815 | 0.663 |
| Prokaryotes | Bacteria | Tenericutes | ayw | Phytoplasma AYWB | 693 | 0.288 |
| Prokaryotes | Bacteria | Alphaproteobacteria | azc | Azorhizobium caulinodans | 4717 | 0.676 |
| Prokaryotes | Bacteria | Alphaproteobacteria | azl | Azospirillum sp. B510 | 6309 | 0.683 |
| Prokaryotes | Bacteria | Betaproteobacteria | azo | Azoarcus sp. BH72 | 3989 | 0.681 |
| Prokaryotes | Bacteria | Gammaproteobacteria | bab | Buchnera aphidicola Bp | 507 | 0.273 |
| Prokaryotes | Bacteria | Betaproteobacteria | bac | Burkholderia ambifaria MC40-6 | 6697 | 0.670 |
| Prokaryotes | Bacteria | Actinobacteria | bad | Bifidobacterium adolescentis | 1632 | 0.601 |
| Prokaryotes | Bacteria | Firmicutes | bae | Bacillus atrophaeus | 4186 | 0.442 |
| Prokaryotes | Bacteria | Spirochaetes | baf | Borrelia afzelii | 1209 | 0.285 |
| Prokaryotes | Bacteria | Firmicutes | bah | Bacillus anthracis CDC 684 | 5902 | 0.361 |
| Prokaryotes | Bacteria | Firmicutes | bai | Bacillus anthracis A0248 | 5291 | 0.361 |
| Prokaryotes | Bacteria | Firmicutes | bal | Bacillus anthracis CI | 5558 | 0.361 |
| Prokaryotes | Bacteria | Betaproteobacteria | bam | Burkholderia cepacia | 6610 | 0.673 |
| Prokaryotes | Bacteria | Firmicutes | ban | Bacillus anthracis Ames | 5328 | 0.362 |
| Prokaryotes | Bacteria | Firmicutes | bao | Bacillus amyloliquefaciens DSM 7 | 3893 | 0.471 |
| Prokaryotes | Bacteria | Gammaproteobacteria | bap | Buchnera aphidicola 5A | 555 | 0.277 |
| Prokaryotes | Bacteria | Firmicutes | bar | Bacillus anthracis Ames 0581 | 5484 | 0.361 |
| Prokaryotes | Bacteria | Gammaproteobacteria | bas | Buchnera aphidicola Sg | 546 | 0.265 |
| Prokaryotes | Bacteria | Firmicutes | bat | Bacillus anthracis Sterne | 5289 | 0.362 |
| Prokaryotes | Bacteria | Gammaproteobacteria | bau | Buchnera aphidicola Tuc7 | 553 | 0.277 |
| Prokaryotes | Bacteria | Betaproteobacteria | bav | Bordetella avium | 3381 | 0.621 |
| Prokaryotes | Bacteria | Firmicutes | bay | Bacillus amyloliquefaciens FZB42 | 3693 | 0.474 |
| Prokaryotes | Bacteria | Deltaproteobacteria | bba | Bdellovibrio bacteriovorus | 3587 | 0.510 |
| Prokaryotes | Bacteria | Firmicutes | bbe | Brevibacillus brevis | 5947 | 0.480 |
| Prokaryotes | Bacteria | Actinobacteria | bbi | Bifidobacterium bifidum S17 | 1783 | 0.636 |
| Prokaryotes | Bacteria | Alphaproteobacteria | bbk | Bartonella bacilliformis | 1283 | 0.396 |
| Prokaryotes | Bacteria | Bacteroidetes | bbl | Blattabacterium sp. (Blattella germanica) | 586 | 0.275 |
| Eukaryotes | Protists | Alveolates | bbo | Babesia bovis | 3706 | 0.439 |
| Prokaryotes | Bacteria | Actinobacteria | bbp | Bifidobacterium bifidum PRL2010 | 1706 | 0.636 |
| Prokaryotes | Bacteria | Betaproteobacteria | bbr | Bordetella bronchiseptica | 4994 | 0.684 |
| Prokaryotes | Bacteria | Alphaproteobacteria | bbt | Bradyrhizobium sp. BTAi1 | 7621 | 0.654 |
| Prokaryotes | Bacteria | Spirochaetes | bbu | Borrelia burgdorferi B31 | 1640 | 0.290 |
| Prokaryotes | Bacteria | Spirochaetes | bbz | Borrelia burgdorferi ZS7 | 1239 | 0.290 |
| Prokaryotes | Bacteria | Firmicutes | bca | Bacillus cereus ATCC 10987 | 5843 | 0.362 |
| Prokaryotes | Bacteria | Firmicutes | bcb | Bacillus cereus B4264 | 5398 | 0.362 |
| Prokaryotes | Bacteria | Gammaproteobacteria | bcc | Buchnera aphidicola Cc | 362 | 0.218 |
| Prokaryotes | Bacteria | Alphaproteobacteria | bcd | Bartonella clarridgeiae | 1326 | 0.375 |
| Prokaryotes | Bacteria | Firmicutes | bce | Bacillus cereus ATCC 14579 | 5255 | 0.362 |
| Prokaryotes | Bacteria | Firmicutes | bcg | Bacillus cereus G9842 | 5857 | 0.359 |
| Prokaryotes | Bacteria | Betaproteobacteria | bch | Burkholderia cenocepacia HI2424 | 6919 | 0.673 |
| Prokaryotes | Bacteria | Gammaproteobacteria | bci | Baumannia cicadellinicola | 595 | 0.346 |
| Prokaryotes | Bacteria | Betaproteobacteria | bcj | Burkholderia cenocepacia J2315 | 7116 | 0.675 |
| Prokaryotes | Bacteria | Firmicutes | bcl | Bacillus clausii | 4096 | 0.454 |
| Prokaryotes | Bacteria | Betaproteobacteria | bcm | Burkholderia cenocepacia MC0-3 | 7008 | 0.672 |
| Prokaryotes | Bacteria | Betaproteobacteria | bcn | Burkholderia cenocepacia AU1054 | 6477 | 0.675 |
| Prokaryotes | Bacteria | Firmicutes | bco | Bacillus cellulosilyticus | 4266 | 0.371 |
| Prokaryotes | Bacteria | Firmicutes | bcq | Bacillus cereus Q1 | 5489 | 0.363 |
| Prokaryotes | Bacteria | Firmicutes | bcr | Bacillus cereus AH187 | 5783 | 0.363 |
| Prokaryotes | Bacteria | Alphaproteobacteria | bcs | Brucella canis | 3251 | 0.582 |
| Prokaryotes | Bacteria | Firmicutes | bcu | Bacillus cereus AH820 | 5810 | 0.362 |
| Prokaryotes | Bacteria | Actinobacteria | bcv | Beutenbergia cavernae | 4197 | 0.731 |
| Prokaryotes | Bacteria | Firmicutes | bcx | Bacillus cereus 03BB102 | 5606 | 0.362 |
| Prokaryotes | Bacteria | Firmicutes | bcy | Bacillus cytotoxis NVH 391-98 | 3844 | 0.367 |
| Prokaryotes | Bacteria | Firmicutes | bcz | Bacillus cereus ZK | 5641 | 0.360 |
| Prokaryotes | Bacteria | Actinobacteria | bde | Bifidobacterium dentium | 2129 | 0.593 |
| Prokaryotes | Bacteria | Spirochaetes | bdu | Borrelia duttonii | 1305 | 0.289 |
| Prokaryotes | Bacteria | Actinobacteria | bfa | Brachybacterium faecium | 3068 | 0.720 |
| Prokaryotes | Bacteria | Gammaproteobacteria | bfl | Candidatus Blochmannia floridanus | 583 | 0.291 |
| Eukaryotes | Animals | Lancelets | bfo | Branchiostoma floridae (Florida lancelet) | 28634 | 0.530 |
| Prokaryotes | Bacteria | Bacteroidetes | bfr | Bacteroides fragilis YCH46 | 4624 | 0.442 |
| Prokaryotes | Bacteria | Bacteroidetes | bfs | Bacteroides fragilis NCTC9343 | 4231 | 0.442 |
| Eukaryotes | Fungi | Ascomycetes | bfu | Botryotinia fuckeliana | 16389 | 0.462 |
| Prokaryotes | Bacteria | Spirochaetes | bga | Borrelia garinii | 929 | 0.287 |
| Prokaryotes | Bacteria | Betaproteobacteria | bgd | Burkholderia gladioli | 7410 | 0.680 |
| Prokaryotes | Bacteria | Betaproteobacteria | bge | Burkholderia sp. CCGE1002 | 6889 | 0.640 |
| Prokaryotes | Bacteria | Betaproteobacteria | bgl | Burkholderia glumae | 5773 | 0.682 |
| Prokaryotes | Bacteria | Alphaproteobacteria | bgr | Bartonella grahamii | 1768 | 0.399 |
| Prokaryotes | Bacteria | Firmicutes | bha | Bacillus halodurans | 4065 | 0.444 |
| Prokaryotes | Bacteria | Alphaproteobacteria | bhe | Bartonella henselae | 1488 | 0.400 |
| Prokaryotes | Bacteria | Bacteroidetes | bhl | Bacteroides helcogenes | 3244 | 0.457 |
| Prokaryotes | Bacteria | Spirochaetes | bhr | Borrelia hermsii | 819 | 0.302 |
| Prokaryotes | Bacteria | Spirochaetes | bhy | Brachyspira hyodysenteriae | 2642 | 0.282 |
| Prokaryotes | Bacteria | Alphaproteobacteria | bid | Beijerinckia indica | 3784 | 0.582 |
| Prokaryotes | Bacteria | Alphaproteobacteria | bja | Bradyrhizobium japonicum | 8317 | 0.647 |
| Prokaryotes | Bacteria | Actinobacteria | bla | Bifidobacterium animalis subsp. lactis AD011 | 1527 | 0.615 |
| Prokaryotes | Bacteria | Actinobacteria | blb | Bifidobacterium longum subsp. longum BBMN68 | 1806 | 0.610 |
| Prokaryotes | Bacteria | Actinobacteria | blc | Bifidobacterium animalis subsp. lactis Bl-04 | 1567 | 0.614 |
| Prokaryotes | Bacteria | Firmicutes | bld | Bacillus licheniformis DSM13 | 4192 | 0.472 |
| Prokaryotes | Bacteria | Actinobacteria | blf | Bifidobacterium longum subsp. infantis 157F | 1999 | 0.610 |
| Prokaryotes | Bacteria | Firmicutes | bli | Bacillus licheniformis ATCC 14580 | 4173 | 0.472 |
| Prokaryotes | Bacteria | Actinobacteria | blj | Bifidobacterium longum DJO10A | 2002 | 0.611 |
| Prokaryotes | Bacteria | Actinobacteria | bll | Bifidobacterium longum subsp. longum JDM301 | 1958 | 0.609 |
| Prokaryotes | Bacteria | Actinobacteria | blm | Bifidobacterium longum subsp. longum JCM 1217 | 1924 | 0.612 |
| Prokaryotes | Bacteria | Actinobacteria | bln | Bifidobacterium longum subsp. infantis ATCC 15697 | 2416 | 0.607 |
| Prokaryotes | Bacteria | Actinobacteria | blo | Bifidobacterium longum NCC2705 | 1729 | 0.609 |
| Prokaryotes | Bacteria | Actinobacteria | blt | Bifidobacterium animalis subsp. lactis DSM 10140 | 1566 | 0.614 |
| Prokaryotes | Bacteria | Betaproteobacteria | bma | Burkholderia mallei ATCC 23344 | 5024 | 0.686 |
| Prokaryotes | Bacteria | Alphaproteobacteria | bmb | Brucella abortus 9-941 | 3084 | 0.582 |
| Prokaryotes | Bacteria | Alphaproteobacteria | bmc | Brucella abortus S19 | 3000 | 0.584 |
| Prokaryotes | Bacteria | Firmicutes | bmd | Bacillus megaterium DSM 319 | 5100 | 0.393 |
| Prokaryotes | Bacteria | Alphaproteobacteria | bme | Brucella melitensis bv. 1 16M | 3198 | 0.583 |
| Prokaryotes | Bacteria | Alphaproteobacteria | bmf | Brucella melitensis biovar Abortus | 3034 | 0.582 |
| Prokaryotes | Bacteria | Alphaproteobacteria | bmi | Brucella melitensis ATCC 23457 | 3135 | 0.582 |
| Prokaryotes | Bacteria | Betaproteobacteria | bmj | Burkholderia multivorans ATCC 17616 (Tohoku) | 6112 | 0.672 |
| Prokaryotes | Bacteria | Betaproteobacteria | bml | Burkholderia mallei NCTC 10229 | 5509 | 0.687 |
| Prokaryotes | Bacteria | Betaproteobacteria | bmn | Burkholderia mallei NCTC 10247 | 5415 | 0.687 |
| Prokaryotes | Bacteria | Firmicutes | bmq | Bacillus megaterium QM B1551 | 5612 | 0.391 |
| Prokaryotes | Bacteria | Alphaproteobacteria | bmr | Brucella microti | 3282 | 0.582 |
| Prokaryotes | Bacteria | Alphaproteobacteria | bms | Brucella suis 1330 | 3271 | 0.582 |
| Prokaryotes | Bacteria | Alphaproteobacteria | bmt | Brucella suis ATCC 23445 | 3241 | 0.582 |
| Prokaryotes | Bacteria | Betaproteobacteria | bmu | Burkholderia multivorans ATCC 17616 (JGI) | 6258 | 0.671 |
| Prokaryotes | Bacteria | Betaproteobacteria | bmv | Burkholderia mallei SAVP1 | 5188 | 0.686 |
| Eukaryotes | Animals | Nematodes | bmy | Brugia malayi (filaria) | 11371 | 0.395 |
| Prokaryotes | Bacteria | Alphaproteobacteria | bov | Brucella ovis | 2890 | 0.583 |
| Prokaryotes | Bacteria | Betaproteobacteria | bpa | Bordetella parapertussis | 4185 | 0.685 |
| Prokaryotes | Bacteria | Firmicutes | bpb | Butyrivibrio proteoclasticus | 3811 | 0.407 |
| Prokaryotes | Bacteria | Betaproteobacteria | bpd | Burkholderia pseudomallei 668 | 7116 | 0.685 |
| Prokaryotes | Bacteria | Betaproteobacteria | bpe | Bordetella pertussis | 3436 | 0.682 |
| Prokaryotes | Bacteria | Firmicutes | bpf | Bacillus pseudofirmus | 4335 | 0.405 |
| Prokaryotes | Bacteria | Betaproteobacteria | bph | Burkholderia phymatum | 7496 | 0.629 |
| Prokaryotes | Bacteria | Bacteroidetes | bpi | Blattabacterium sp. (Periplaneta americana) | 582 | 0.286 |
| Prokaryotes | Bacteria | Betaproteobacteria | bpl | Burkholderia pseudomallei 1106a | 7174 | 0.686 |
| Prokaryotes | Bacteria | Betaproteobacteria | bpm | Burkholderia pseudomallei 1710b | 6344 | 0.687 |
| Prokaryotes | Bacteria | Gammaproteobacteria | bpn | Candidatus Blochmannia pennsylvanicus | 610 | 0.323 |
| Prokaryotes | Bacteria | Spirochaetes | bpo | Brachyspira pilosicoli | 2299 | 0.289 |
| Prokaryotes | Bacteria | Betaproteobacteria | bpr | Burkholderia pseudomallei MSHR346 | 4202 | 0.680 |
| Prokaryotes | Bacteria | Betaproteobacteria | bps | Burkholderia pseudomallei K96243 | 5728 | 0.683 |
| Prokaryotes | Bacteria | Betaproteobacteria | bpt | Bordetella petrii | 5027 | 0.659 |
| Prokaryotes | Bacteria | Firmicutes | bpu | Bacillus pumilus | 3678 | 0.419 |
| Prokaryotes | Bacteria | Betaproteobacteria | bpy | Burkholderia phytofirmans | 7241 | 0.630 |
| Prokaryotes | Bacteria | Alphaproteobacteria | bqu | Bartonella quintana | 1142 | 0.404 |
| Prokaryotes | Bacteria | Alphaproteobacteria | bra | Bradyrhizobium sp. ORS278 | 6717 | 0.661 |
| Prokaryotes | Bacteria | Spirochaetes | bre | Borrelia recurrentis | 990 | 0.282 |
| Prokaryotes | Bacteria | Betaproteobacteria | brh | Burkholderia rhizoxinica | 3870 | 0.612 |
| Prokaryotes | Bacteria | Spirochaetes | brm | Brachyspira murdochii | 2809 | 0.291 |
| Prokaryotes | Bacteria | Bacteroidetes | bsa | Bacteroides salanitronis | 3641 | 0.477 |
| Prokaryotes | Bacteria | Alphaproteobacteria | bsb | Brevundimonas subvibrioides | 3327 | 0.688 |
| Prokaryotes | Bacteria | Firmicutes | bse | Bacillus selenitireducens | 3255 | 0.495 |
| Prokaryotes | Bacteria | Firmicutes | bsn | Bacillus subtilis BSn5 | 4145 | 0.447 |
| Prokaryotes | Bacteria | Firmicutes | bss | Bacillus subtilis subsp. spizizenii | 4062 | 0.447 |
| Prokaryotes | Bacteria | Firmicutes | bsu | Bacillus subtilis | 4176 | 0.444 |
| Eukaryotes | Animals | Vertebrates | bta | Bos taurus (cow) | 22769 | 0.536 |
| Prokaryotes | Bacteria | Firmicutes | btb | Bacillus thuringiensis BMB171 | 5350 | 0.361 |
| Prokaryotes | Bacteria | Betaproteobacteria | bte | Burkholderia thailandensis | 5632 | 0.680 |
| Prokaryotes | Bacteria | Bacteroidetes | bth | Bacteroides thetaiotaomicron | 4816 | 0.439 |
| Prokaryotes | Bacteria | Firmicutes | btk | Bacillus thuringiensis 97-27 | 5197 | 0.362 |
| Prokaryotes | Bacteria | Firmicutes | btl | Bacillus thuringiensis Al Hakam | 4798 | 0.363 |
| Prokaryotes | Bacteria | Alphaproteobacteria | btr | Bartonella tribocorum | 2087 | 0.408 |
| Prokaryotes | Bacteria | Firmicutes | bts | Bacillus tusciae | 3150 | 0.595 |
| Prokaryotes | Bacteria | Spirochaetes | btu | Borrelia turicatae | 818 | 0.295 |
| Prokaryotes | Bacteria | Gammaproteobacteria | buc | Buchnera aphidicola APS | 574 | 0.277 |
| Prokaryotes | Bacteria | Betaproteobacteria | bug | Burkholderia sp. CCGE1001 | 5965 | 0.641 |
| Prokaryotes | Bacteria | Betaproteobacteria | bur | Burkholderia sp. 383 | 7716 | 0.668 |
| Prokaryotes | Bacteria | Gammaproteobacteria | bva | Candidatus Blochmannia vafer | 587 | 0.294 |
| Prokaryotes | Bacteria | Betaproteobacteria | bvi | Burkholderia vietnamiensis | 7617 | 0.661 |
| Prokaryotes | Bacteria | Bacteroidetes | bvu | Bacteroides vulgatus | 4066 | 0.433 |
| Prokaryotes | Bacteria | Firmicutes | bwe | Bacillus weihenstephanensis | 5653 | 0.364 |
| Prokaryotes | Bacteria | Betaproteobacteria | bxe | Burkholderia xenovorans | 8702 | 0.632 |
| Prokaryotes | Bacteria | Verrucomicrobia | caa | Coraliomargarita akajimensis | 3120 | 0.539 |
| Prokaryotes | Bacteria | Chlamydiae | cab | Chlamydophila abortus | 932 | 0.406 |
| Prokaryotes | Bacteria | Firmicutes | cac | Clostridium acetobutylicum | 3847 | 0.318 |
| Prokaryotes | Bacteria | Green nonsulfur bacteria | cag | Chloroflexus aggregans | 3731 | 0.568 |
| Prokaryotes | Bacteria | Actinobacteria | cai | Catenulispora acidiphila | 8913 | 0.702 |
| Prokaryotes | Bacteria | Alphaproteobacteria | cak | Caulobacter sp. K31 | 5438 | 0.680 |
| Eukaryotes | Fungi | Ascomycetes | cal | Candida albicans | 14629 | 0.352 |
| Prokaryotes | Bacteria | Bacteroidetes | cao | Cellulophaga algicola | 4163 | 0.344 |
| Prokaryotes | Bacteria | Actinobacteria | car | Corynebacterium aurimucosum | 2551 | 0.613 |
| Prokaryotes | Bacteria | Bacteroidetes | cat | Croceibacter atlanticus | 2702 | 0.344 |
| Prokaryotes | Bacteria | Green nonsulfur bacteria | cau | Chloroflexus aurantiacus | 3853 | 0.572 |
| Prokaryotes | Bacteria | Firmicutes | cba | Clostridium botulinum A ATCC 19397 | 3550 | 0.294 |
| Prokaryotes | Bacteria | Firmicutes | cbb | Clostridium botulinum B1 Okra | 3846 | 0.295 |
| Prokaryotes | Bacteria | Gammaproteobacteria | cbc | Coxiella burnetii CbuK_Q154 | 1942 | 0.429 |
| Prokaryotes | Bacteria | Gammaproteobacteria | cbd | Coxiella burnetii Dugway 5J108-111 | 2045 | 0.426 |
| Prokaryotes | Bacteria | Firmicutes | cbe | Clostridium beijerinckii | 5020 | 0.312 |
| Prokaryotes | Bacteria | Firmicutes | cbf | Clostridium botulinum F Langeland | 3655 | 0.296 |
| Prokaryotes | Bacteria | Gammaproteobacteria | cbg | Coxiella burnetii CbuG_Q212 | 1866 | 0.429 |
| Prokaryotes | Bacteria | Firmicutes | cbh | Clostridium botulinum A Hall | 3401 | 0.294 |
| Prokaryotes | Bacteria | Firmicutes | cbi | Clostridium botulinum Ba4 | 4002 | 0.292 |
| Prokaryotes | Bacteria | Firmicutes | cbk | Clostridium botulinum B Eklund 17B | 3475 | 0.287 |
| Prokaryotes | Bacteria | Firmicutes | cbl | Clostridium botulinum A3 Loch Maree | 3983 | 0.293 |
| Prokaryotes | Bacteria | Firmicutes | cbn | Clostridium botulinum BKT015925 | 2994 | 0.291 |
| Prokaryotes | Bacteria | Firmicutes | cbo | Clostridium botulinum A ATCC 3502 | 3590 | 0.295 |
| Eukaryotes | Animals | Nematodes | cbr | Caenorhabditis briggsae | 19414 | 0.442 |
| Prokaryotes | Bacteria | Gammaproteobacteria | cbs | Coxiella burnetii RSA 331 | 1975 | 0.430 |
| Prokaryotes | Bacteria | Firmicutes | cbt | Clostridium botulinum E3 | 3257 | 0.287 |
| Prokaryotes | Bacteria | Gammaproteobacteria | cbu | Coxiella burnetii RSA 493 | 1847 | 0.429 |
| Prokaryotes | Bacteria | Firmicutes | cby | Clostridium botulinum A2 | 3877 | 0.293 |
| Prokaryotes | Bacteria | Chlamydiae | cca | Chlamydophila caviae | 1005 | 0.399 |
| Prokaryotes | Bacteria | Firmicutes | ccb | Clostridium cellulovorans | 4254 | 0.322 |
| Prokaryotes | Bacteria | Firmicutes | cce | Clostridium cellulolyticum | 3390 | 0.383 |
| Prokaryotes | Bacteria | Green sulfur bacteria | cch | Chlorobium chlorochromatii | 2002 | 0.451 |
| Eukaryotes | Fungi | Basidiomycetes | cci | Coprinopsis cinerea | 13356 | 0.540 |
| Prokaryotes | Bacteria | Epsilonproteobacteria | cco | Campylobacter concisus 13826 | 1985 | 0.402 |
| Prokaryotes | Bacteria | Alphaproteobacteria | ccr | Caulobacter crescentus CB15 | 3737 | 0.676 |
| Prokaryotes | Bacteria | Alphaproteobacteria | ccs | Caulobacter crescentus NA1000 | 3878 | 0.676 |
| Prokaryotes | Bacteria | Actinobacteria | ccu | Cryptobacterium curtum | 1357 | 0.514 |
| Prokaryotes | Bacteria | Epsilonproteobacteria | ccv | Campylobacter curvus | 1931 | 0.454 |
| Prokaryotes | Bacteria | Firmicutes | cdc | Clostridium difficile CD196 | 3454 | 0.299 |
| Prokaryotes | Bacteria | Firmicutes | cdf | Clostridium difficile 630 | 3749 | 0.305 |
| Prokaryotes | Bacteria | Actinobacteria | cdi | Corynebacterium diphtheriae | 2272 | 0.541 |
| Prokaryotes | Bacteria | Firmicutes | cdl | Clostridium difficile R20291 | 3507 | 0.301 |
| Eukaryotes | Fungi | Ascomycetes | cdu | Candida dubliniensis | 5860 | 0.348 |
| Prokaryotes | Bacteria | Actinobacteria | cef | Corynebacterium efficiens | 2994 | 0.636 |
| Eukaryotes | Animals | Nematodes | cel | Caenorhabditis elegans (nematode) | 20183 | 0.427 |
| Eukaryotes | Animals | Vertebrates | cfa | Canis familiaris (dog) | 19836 | 0.534 |
| Prokaryotes | Bacteria | Chlamydiae | cfe | Chlamydophila felis | 1013 | 0.400 |
| Prokaryotes | Bacteria | Epsilonproteobacteria | cff | Campylobacter fetus | 1719 | 0.341 |
| Prokaryotes | Bacteria | Actinobacteria | cfl | Cellulomonas flavigena | 3678 | 0.743 |
| Prokaryotes | Bacteria | Actinobacteria | cgb | Corynebacterium glutamicum ATCC 13032 (Bielefeld) | 3057 | 0.548 |
| Prokaryotes | Bacteria | Actinobacteria | cgl | Corynebacterium glutamicum ATCC 13032 (Kyowa Hakko) | 2993 | 0.548 |
| Prokaryotes | Bacteria | Actinobacteria | cgo | Coriobacterium glomerans | 1768 | 0.609 |
| Eukaryotes | Fungi | Ascomycetes | cgr | Candida glabrata | 5213 | 0.405 |
| Prokaryotes | Bacteria | Actinobacteria | cgt | Corynebacterium glutamicum R | 3080 | 0.552 |
| Prokaryotes | Bacteria | Epsilonproteobacteria | cha | Campylobacter hominis ATCC BAA-381 | 1687 | 0.331 |
| Prokaryotes | Bacteria | Firmicutes | chd | Caldicellulosiruptor hydrothermalis | 2546 | 0.366 |
| Prokaryotes | Bacteria | Green nonsulfur bacteria | chl | Chloroflexus sp. Y-400-fl | 4159 | 0.572 |
| Eukaryotes | Protists | Alveolates | cho | Cryptosporidium hominis | 3885 | 0.327 |
| Prokaryotes | Bacteria | Chlamydiae | chp | Chlamydophila psittaci | 975 | 0.397 |
| Prokaryotes | Bacteria | Bacteroidetes | chu | Cytophaga hutchinsonii | 3785 | 0.397 |
| Prokaryotes | Bacteria | Firmicutes | chy | Carboxydothermus hydrogenoformans | 2620 | 0.426 |
| Eukaryotes | Fungi | Ascomycetes | cim | Coccidioides immitis | 10440 | 0.504 |
| Eukaryotes | Animals | Ascidians | cin | Ciona intestinalis (sea squirt) | 13441 | 0.427 |
| Prokaryotes | Bacteria | Gammaproteobacteria | cja | Cellvibrio japonicus | 3750 | 0.527 |
| Prokaryotes | Bacteria | Epsilonproteobacteria | cjd | Campylobacter jejuni subsp. doylei 269.97 | 1731 | 0.312 |
| Prokaryotes | Bacteria | Epsilonproteobacteria | cje | Campylobacter jejuni NCTC11168 | 1623 | 0.310 |
| Prokaryotes | Bacteria | Epsilonproteobacteria | cjj | Campylobacter jejuni 81-176 | 1758 | 0.310 |
| Prokaryotes | Bacteria | Actinobacteria | cjk | Corynebacterium jeikeium | 2120 | 0.622 |
| Prokaryotes | Bacteria | Epsilonproteobacteria | cjn | Campylobacter jejuni ICDCCJ07001 | 1531 | 0.312 |
| Prokaryotes | Bacteria | Epsilonproteobacteria | cjr | Campylobacter jejuni RM1221 | 1838 | 0.308 |
| Prokaryotes | Bacteria | Epsilonproteobacteria | cju | Campylobacter jejuni 81116 | 1626 | 0.311 |
| Prokaryotes | Bacteria | Firmicutes | cki | Caldicellulosiruptor kristjanssonii | 2482 | 0.366 |
| Prokaryotes | Bacteria | Firmicutes | ckl | Clostridium kluyveri DSM 555 | 3913 | 0.330 |
| Prokaryotes | Bacteria | Firmicutes | ckn | Caldicellulosiruptor kronotskyensis | 2466 | 0.356 |
| Prokaryotes | Bacteria | Gammaproteobacteria | cko | Citrobacter koseri ATCC BAA-895 | 5003 | 0.546 |
| Prokaryotes | Bacteria | Actinobacteria | ckp | Corynebacterium kroppenstedtii | 2018 | 0.581 |
| Prokaryotes | Bacteria | Firmicutes | ckr | Clostridium kluyveri NBRC 12016 | 3523 | 0.330 |
| Prokaryotes | Bacteria | Epsilonproteobacteria | cla | Campylobacter lari | 1545 | 0.301 |
| Prokaryotes | Bacteria | Firmicutes | cle | Clostridium lentocellum | 4182 | 0.351 |
| Prokaryotes | Bacteria | Green sulfur bacteria | cli | Chlorobium limicola | 2434 | 0.522 |
| Prokaryotes | Bacteria | Firmicutes | clj | Clostridium ljungdahlii | 4184 | 0.320 |
| Prokaryotes | Bacteria | Firmicutes | clo | Clostridiales genomosp. BVAB3 | 1567 | 0.449 |
| Eukaryotes | Fungi | Ascomycetes | clu | Clavispora lusitaniae | 5936 | 0.468 |
| Prokaryotes | Bacteria | Bacteroidetes | cly | Cellulophaga lytica | 3284 | 0.328 |
| Prokaryotes | Archaea | Crenarchaeota | cma | Caldivirga maquilingensis | 1963 | 0.440 |
| Eukaryotes | Plants | Red algae | cme | Cyanidioschyzon merolae | 5013 | 0.567 |
| Prokaryotes | Bacteria | Actinobacteria | cmi | Clavibacter michiganensis subsp. michiganensis | 3078 | 0.726 |
| Prokaryotes | Bacteria | Actinobacteria | cms | Clavibacter michiganensis subsp. sepedonicus | 3116 | 0.723 |
| Prokaryotes | Bacteria | Chlamydiae | cmu | Chlamydia muridarum | 911 | 0.408 |
| Eukaryotes | Fungi | Basidiomycetes | cnb | Cryptococcus neoformans B-3501A | 6500 | 0.511 |
| Eukaryotes | Fungi | Basidiomycetes | cne | Cryptococcus neoformans JEC21 | 6273 | 0.512 |
| Prokaryotes | Bacteria | Hyperthermophilic bacteria | cni | Calditerrivibrio nitroreducens | 2100 | 0.360 |
| Prokaryotes | Bacteria | Firmicutes | cno | Clostridium novyi | 2315 | 0.296 |
| Prokaryotes | Bacteria | Firmicutes | cob | Caldicellulosiruptor obsidiansis | 2188 | 0.358 |
| Prokaryotes | Bacteria | Bacteroidetes | coc | Capnocytophaga ochracea | 2171 | 0.406 |
| Prokaryotes | Bacteria | Firmicutes | cow | Caldicellulosiruptor owensensis | 2147 | 0.360 |
| Prokaryotes | Bacteria | Chlamydiae | cpa | Chlamydophila pneumoniae AR39 | 1112 | 0.414 |
| Prokaryotes | Bacteria | Green sulfur bacteria | cpb | Chlorobium phaeobacteroides BS1 | 2469 | 0.498 |
| Prokaryotes | Bacteria | Green sulfur bacteria | cpc | Chlorobaculum parvum NCIB 8327 | 2043 | 0.571 |
| Prokaryotes | Bacteria | Firmicutes | cpe | Clostridium perfringens 13 | 2723 | 0.299 |
| Prokaryotes | Bacteria | Firmicutes | cpf | Clostridium perfringens ATCC 13124 | 2876 | 0.298 |
| Prokaryotes | Bacteria | Green sulfur bacteria | cph | Chlorobium phaeobacteroides DSM 266 | 2650 | 0.492 |
| Prokaryotes | Bacteria | Bacteroidetes | cpi | Chitinophaga pinensis | 7192 | 0.462 |
| Prokaryotes | Bacteria | Chlamydiae | cpj | Chlamydophila pneumoniae J138 | 1069 | 0.414 |
| Prokaryotes | Bacteria | Chlamydiae | cpm | Chlamydophila pecorum | 988 | 0.417 |
| Prokaryotes | Bacteria | Chlamydiae | cpn | Chlamydophila pneumoniae CWL029 | 1052 | 0.414 |
| Prokaryotes | Bacteria | Firmicutes | cpo | Coprothermobacter proteolyticus | 1482 | 0.451 |
| Prokaryotes | Bacteria | Firmicutes | cpr | Clostridium perfringens SM101 | 2566 | 0.297 |
| Prokaryotes | Bacteria | Gammaproteobacteria | cps | Colwellia psychrerythraea | 4910 | 0.390 |
| Prokaryotes | Bacteria | Chlamydiae | cpt | Chlamydophila pneumoniae TW183 | 1113 | 0.413 |
| Prokaryotes | Bacteria | Actinobacteria | cpu | Corynebacterium pseudotuberculosis | 2110 | 0.529 |
| Eukaryotes | Protists | Alveolates | cpv | Cryptosporidium parvum | 3805 | 0.318 |
| Eukaryotes | Fungi | Ascomycetes | cpw | Coccidioides posadasii | 7229 | 0.509 |
| Prokaryotes | Bacteria | Firmicutes | cpy | Clostridium phytofermentans | 3902 | 0.364 |
| Eukaryotes | Animals | Arthropods | cqu | Culex quinquefasciatus (southern house mosquito) | 18896 | 0.552 |
| Eukaryotes | Plants | Green algae | cre | Chlamydomonas reinhardtii | 14416 | 0.678 |
| Prokaryotes | Bacteria | Firmicutes | crn | Carnobacterium sp. 17-4 | 2474 | 0.362 |
| Prokaryotes | Bacteria | Gammaproteobacteria | cro | Citrobacter rodentium | 4913 | 0.557 |
| Prokaryotes | Bacteria | Gammaproteobacteria | crp | Candidatus Carsonella ruddii | 182 | 0.168 |
| Prokaryotes | Bacteria | Gammaproteobacteria | csa | Chromohalobacter salexigens | 3298 | 0.644 |
| Prokaryotes | Bacteria | Firmicutes | csc | Caldicellulosiruptor saccharolyticus | 2679 | 0.358 |
| Prokaryotes | Bacteria | Alphaproteobacteria | cse | Caulobacter segnis | 4139 | 0.682 |
| Prokaryotes | Bacteria | Firmicutes | csh | Clostridium saccharolyticum | 4154 | 0.459 |
| Prokaryotes | Bacteria | Firmicutes | cst | Clostridium sticklandii | 2573 | 0.340 |
| Prokaryotes | Archaea | Thaumarchaeota | csy | Cenarchaeum symbiosum A | 2017 | 0.577 |
| Prokaryotes | Bacteria | Chlamydiae | cta | Chlamydia trachomatis A/HAR-13 (serovar A) | 919 | 0.417 |
| Prokaryotes | Bacteria | Chlamydiae | ctb | Chlamydia trachomatis 434/Bu | 874 | 0.418 |
| Prokaryotes | Bacteria | Firmicutes | ctc | Clostridium tetani E88 | 2436 | 0.293 |
| Prokaryotes | Bacteria | Green sulfur bacteria | cte | Chlorobaculum tepidum | 2245 | 0.576 |
| Prokaryotes | Bacteria | Firmicutes | cth | Clostridium thermocellum | 3189 | 0.404 |
| Prokaryotes | Bacteria | Betaproteobacteria | cti | Cupriavidus taiwanensis | 5896 | 0.675 |
| Prokaryotes | Bacteria | Chlamydiae | ctj | Chlamydia trachomatis B/Jali20/OT | 875 | 0.418 |
| Prokaryotes | Bacteria | Chlamydiae | ctl | Chlamydia trachomatis L2b/UCH-1/proctitis | 874 | 0.418 |
| Eukaryotes | Fungi | Ascomycetes | ctp | Candida tropicalis | 6254 | 0.345 |
| Prokaryotes | Bacteria | Chlamydiae | ctr | Chlamydia trachomatis D/UW-3/CX (serovar D) | 895 | 0.418 |
| Prokaryotes | Bacteria | Green sulfur bacteria | cts | Chloroherpeton thalassium | 2710 | 0.462 |
| Prokaryotes | Bacteria | Betaproteobacteria | ctt | Comamonas testosteroni | 4799 | 0.625 |
| Prokaryotes | Bacteria | Gammaproteobacteria | ctu | Cronobacter turicensis | 4455 | 0.583 |
| Prokaryotes | Bacteria | Chlamydiae | ctz | Chlamydia trachomatis B/TZ1A828/OT | 880 | 0.418 |
| Prokaryotes | Bacteria | Actinobacteria | cur | Corynebacterium urealyticum | 2022 | 0.648 |
| Prokaryotes | Bacteria | Betaproteobacteria | cvi | Chromobacterium violaceum | 4407 | 0.656 |
| Prokaryotes | Bacteria | Actinobacteria | cwo | Conexibacter woesei | 5914 | 0.729 |
| Prokaryotes | Bacteria | Cyanobacteria | cya | Cyanobacteria Yellowstone A-Prime | 2760 | 0.611 |
| Prokaryotes | Bacteria | Cyanobacteria | cyb | Cyanobacteria Yellowstone B-Prime | 2862 | 0.593 |
| Prokaryotes | Bacteria | Cyanobacteria | cyc | Cyanothece sp. PCC 7424 | 5710 | 0.395 |
| Prokaryotes | Bacteria | Cyanobacteria | cyh | Cyanothece sp. PCC 8802 | 4444 | 0.406 |
| Prokaryotes | Bacteria | Cyanobacteria | cyj | Cyanothece sp. PCC 7822 | 6642 | 0.407 |
| Prokaryotes | Bacteria | Cyanobacteria | cyn | Cyanothece sp. PCC 7425 | 5327 | 0.516 |
| Prokaryotes | Bacteria | Cyanobacteria | cyp | Cyanothece sp. PCC 8801 | 4367 | 0.405 |
| Prokaryotes | Bacteria | Cyanobacteria | cyt | Cyanothece sp. ATCC 51142 | 5304 | 0.388 |
| Prokaryotes | Bacteria | Cyanobacteria | cyu | Cyanobacterium UCYN-A | 1199 | 0.333 |
| Prokaryotes | Bacteria | Betaproteobacteria | dac | Delftia acidovorans | 6040 | 0.672 |
| Prokaryotes | Bacteria | Firmicutes | dae | Desulfotomaculum acetoxidans | 4068 | 0.428 |
| Prokaryotes | Bacteria | Deltaproteobacteria | dak | Desulfurivibrio alkaliphilus | 2620 | 0.611 |
| Prokaryotes | Bacteria | Deltaproteobacteria | dal | Desulfatibacillum alkenivorans | 5252 | 0.555 |
| Eukaryotes | Animals | Arthropods | dan | Drosophila ananassae | 15069 | 0.546 |
| Prokaryotes | Bacteria | Deltaproteobacteria | dao | Desulfobacca acetoxidans DSM 11109 | 2866 | 0.542 |
| Prokaryotes | Bacteria | Hyperthermophilic bacteria | dap | Denitrovibrio acetiphilus | 2964 | 0.432 |
| Prokaryotes | Bacteria | Betaproteobacteria | dar | Dechloromonas aromatica | 4171 | 0.598 |
| Prokaryotes | Bacteria | Deltaproteobacteria | das | Desulfovibrio aespoeensis | 3304 | 0.632 |
| Prokaryotes | Bacteria | Deltaproteobacteria | dat | Desulfobacterium autotrophicum | 4943 | 0.497 |
| Prokaryotes | Bacteria | Firmicutes | dau | Candidatus Desulforudis audaxviator | 2157 | 0.619 |
| Prokaryotes | Bacteria | Deltaproteobacteria | dba | Desulfomicrobium baculatum | 3436 | 0.595 |
| Prokaryotes | Bacteria | Deltaproteobacteria | dbr | Desulfarculus baarsii | 3277 | 0.662 |
| Prokaryotes | Bacteria | Gammaproteobacteria | dda | Dickeya dadantii Ech703 | 3970 | 0.562 |
| Prokaryotes | Bacteria | Gammaproteobacteria | ddc | Dickeya dadantii Ech586 | 4144 | 0.549 |
| Prokaryotes | Bacteria | Gammaproteobacteria | ddd | Dickeya dadantii 3937 | 4549 | 0.576 |
| Prokaryotes | Bacteria | Deltaproteobacteria | dde | Desulfovibrio desulfuricans G20 | 3776 | 0.581 |
| Prokaryotes | Bacteria | Hyperthermophilic bacteria | ddf | Deferribacter desulfuricans SSM1 | 2374 | 0.307 |
| Eukaryotes | Protists | Amoebozoa | ddi | Dictyostelium discoideum (cellular slime mold) | 13289 | 0.274 |
| Prokaryotes | Bacteria | Deinococcus | ddr | Deinococcus deserti | 3451 | 0.638 |
| Prokaryotes | Bacteria | Deltaproteobacteria | dds | Desulfovibrio desulfuricans ATCC 27774 | 2356 | 0.588 |
| Prokaryotes | Bacteria | Green nonsulfur bacteria | deb | Dehalococcoides sp. BAV1 | 1371 | 0.480 |
| Prokaryotes | Bacteria | Green nonsulfur bacteria | deg | Dehalococcoides sp. GT | 1417 | 0.481 |
| Prokaryotes | Bacteria | Green nonsulfur bacteria | deh | Dehalococcoides sp. CBDB1 | 1458 | 0.479 |
| Eukaryotes | Animals | Arthropods | der | Drosophila erecta | 15044 | 0.545 |
| Prokaryotes | Bacteria | Green nonsulfur bacteria | det | Dehalococcoides ethenogenes | 1580 | 0.497 |
| Prokaryotes | Bacteria | Green nonsulfur bacteria | dev | Dehalococcoides sp. VS | 1439 | 0.481 |
| Prokaryotes | Bacteria | Bacteroidetes | dfe | Dyadobacter fermentans | 5719 | 0.526 |
| Prokaryotes | Bacteria | Deinococcus | dge | Deinococcus geothermalis | 3054 | 0.668 |
| Eukaryotes | Animals | Arthropods | dgr | Drosophila grimshawi | 14982 | 0.518 |
| Eukaryotes | Fungi | Ascomycetes | dha | Debaryomyces hansenii | 6286 | 0.375 |
| Prokaryotes | Bacteria | Firmicutes | dhd | Desulfitobacterium hafniense DCB-2 | 4883 | 0.486 |
| Prokaryotes | Bacteria | Betaproteobacteria | dia | Acidovorax ebreus | 3479 | 0.674 |
| Prokaryotes | Bacteria | Chrysiogenetes | din | Desulfurispirillum indicum | 2571 | 0.567 |
| Prokaryotes | Archaea | Crenarchaeota | dka | Desulfurococcus kamchatkensis | 1471 | 0.459 |
| Prokaryotes | Bacteria | Green nonsulfur bacteria | dly | Dehalogenimonas lykanthroporepellens | 1659 | 0.559 |
| Prokaryotes | Bacteria | Deltaproteobacteria | dma | Desulfovibrio magneticus | 4700 | 0.634 |
| Eukaryotes | Animals | Arthropods | dme | Drosophila melanogaster (fruit fly) | 13776 | 0.532 |
| Eukaryotes | Animals | Arthropods | dmo | Drosophila mojavensis | 14594 | 0.531 |
| Prokaryotes | Bacteria | Deinococcus | dmr | Deinococcus maricopensis | 3264 | 0.701 |
| Prokaryotes | Archaea | Crenarchaeota | dmu | Desulfurococcus mucosus | 1345 | 0.535 |
| Prokaryotes | Bacteria | Gammaproteobacteria | dno | Dichelobacter nodosus | 1280 | 0.451 |
| Prokaryotes | Bacteria | Deltaproteobacteria | dol | Desulfococcus oleovorans | 3265 | 0.572 |
| Eukaryotes | Animals | Arthropods | dpe | Drosophila persimilis | 16874 | 0.553 |
| Eukaryotes | Animals | Arthropods | dpo | Drosophila pseudoobscura pseudoobscura | 16071 | 0.557 |
| Prokaryotes | Bacteria | Deltaproteobacteria | dpr | Desulfobulbus propionicus | 3283 | 0.596 |
| Prokaryotes | Bacteria | Deltaproteobacteria | dps | Desulfotalea psychrophila | 3234 | 0.476 |
| Prokaryotes | Bacteria | Deinococcus | dpt | Deinococcus proteolyticus | 2656 | 0.661 |
| Prokaryotes | Bacteria | Deinococcus | dra | Deinococcus radiodurans | 3167 | 0.672 |
| Eukaryotes | Animals | Vertebrates | dre | Danio rerio (zebrafish) | 26976 | 0.496 |
| Prokaryotes | Bacteria | Firmicutes | drm | Desulfotomaculum reducens | 3276 | 0.434 |
| Prokaryotes | Bacteria | Deltaproteobacteria | drt | Desulfohalobium retbaense | 2526 | 0.579 |
| Prokaryotes | Bacteria | Deltaproteobacteria | dsa | Desulfovibrio salexigens | 3807 | 0.481 |
| Eukaryotes | Animals | Arthropods | dse | Drosophila sechellia | 16480 | 0.540 |
| Prokaryotes | Bacteria | Alphaproteobacteria | dsh | Dinoroseobacter shibae | 4192 | 0.659 |
| Eukaryotes | Animals | Arthropods | dsi | Drosophila simulans | 15426 | 0.537 |
| Prokaryotes | Bacteria | Firmicutes | dsy | Desulfitobacterium hafniense Y51 | 5060 | 0.485 |
| Prokaryotes | Bacteria | Hyperthermophilic bacteria | dte | Desulfurobacterium thermolithotrophum | 1509 | 0.350 |
| Prokaryotes | Bacteria | Hyperthermophilic bacteria | dth | Dictyoglomus thermophilum | 1912 | 0.340 |
| Prokaryotes | Bacteria | Hyperthermophilic bacteria | dtu | Dictyoglomus turgidum | 1744 | 0.342 |
| Eukaryotes | Animals | Arthropods | dvi | Drosophila virilis | 14491 | 0.531 |
| Prokaryotes | Bacteria | Deltaproteobacteria | dvl | Desulfovibrio vulgaris DP4 | 3091 | 0.635 |
| Prokaryotes | Bacteria | Deltaproteobacteria | dvm | Desulfovibrio vulgaris Miyazaki F | 3180 | 0.674 |
| Prokaryotes | Bacteria | Deltaproteobacteria | dvu | Desulfovibrio vulgaris Hildenborough | 3536 | 0.635 |
| Eukaryotes | Animals | Arthropods | dwi | Drosophila willistoni | 15512 | 0.472 |
| Eukaryotes | Animals | Arthropods | dya | Drosophila yakuba | 16090 | 0.542 |
| Prokaryotes | Bacteria | Gammaproteobacteria | dze | Dickeya zeae | 4163 | 0.558 |
| Prokaryotes | Bacteria | Gammaproteobacteria | eam | Erwinia amylovora CFBP1430 | 3706 | 0.547 |
| Prokaryotes | Bacteria | Firmicutes | eat | Exiguobacterium sp. AT1b | 3020 | 0.492 |
| Eukaryotes | Plants | Eudicots | eath | Arabidopsis thaliana (thale cress) (EST) | 27361 | 0.441 |
| Prokaryotes | Bacteria | Gammaproteobacteria | eay | Erwinia amylovora ATCC 49946 | 3565 | 0.546 |
| Prokaryotes | Bacteria | Betaproteobacteria | eba | Aromatoleum aromaticum EbN1 | 4590 | 0.650 |
| Prokaryotes | Bacteria | Gammaproteobacteria | ebd | Escherichia coli BL21-Gold(DE3)pLysS AG | 4228 | 0.519 |
| Prokaryotes | Bacteria | Gammaproteobacteria | ebi | Erwinia billingiae | 4917 | 0.562 |
| Prokaryotes | Bacteria | Gammaproteobacteria | ebr | Escherichia coli B REL606 | 4205 | 0.519 |
| Prokaryotes | Bacteria | Gammaproteobacteria | ebw | Escherichia coli K-12 MC4100(MuLac) BW2952 | 4084 | 0.520 |
| Prokaryotes | Bacteria | Gammaproteobacteria | eca | Pectobacterium atrosepticum | 4472 | 0.522 |
| Eukaryotes | Animals | Vertebrates | ecb | Equus caballus (horse) | 17677 | 0.524 |
| Prokaryotes | Bacteria | Gammaproteobacteria | ecc | Escherichia coli O6:K2:H1 CFT073 (UPEC) | 5333 | 0.515 |
| Prokaryotes | Bacteria | Gammaproteobacteria | ecd | Escherichia coli K-12 DH10B | 4126 | 0.519 |
| Prokaryotes | Bacteria | Gammaproteobacteria | ece | Escherichia coli O157:H7 EDL933 (EHEC) | 5397 | 0.515 |
| Prokaryotes | Bacteria | Gammaproteobacteria | ecf | Escherichia coli O157:H7 EC4115 (EHEC) | 5477 | 0.515 |
| Prokaryotes | Bacteria | Gammaproteobacteria | ecg | Escherichia coli O127:H6 E2348/69 (EPEC) | 4651 | 0.511 |
| Prokaryotes | Bacteria | Alphaproteobacteria | ech | Ehrlichia chaffeensis | 1105 | 0.316 |
| Prokaryotes | Bacteria | Gammaproteobacteria | eci | Escherichia coli O18:K1:H7 UTI89 (UPEC) | 5166 | 0.516 |
| Prokaryotes | Bacteria | Gammaproteobacteria | ecj | Escherichia coli K-12 W3110 | 4226 | 0.519 |
| Prokaryotes | Bacteria | Gammaproteobacteria | eck | Escherichia coli 55989 (EAEC) | 4759 | 0.517 |
| Prokaryotes | Bacteria | Gammaproteobacteria | ecl | Escherichia coli C ATCC 8739 | 4199 | 0.519 |
| Prokaryotes | Bacteria | Gammaproteobacteria | ecm | Escherichia coli SMS-3-5 (environmental) | 4913 | 0.515 |
| Prokaryotes | Bacteria | Alphaproteobacteria | ecn | Ehrlichia canis | 925 | 0.311 |
| Prokaryotes | Bacteria | Gammaproteobacteria | eco | Escherichia coli K-12 MG1655 | 4145 | 0.519 |
| Prokaryotes | Bacteria | Gammaproteobacteria | ecp | Escherichia coli O6:K15:H31 536 (UPEC) | 4619 | 0.516 |
| Prokaryotes | Bacteria | Gammaproteobacteria | ecq | Escherichia coli O81 ED1a (commensal) | 4915 | 0.518 |
| Prokaryotes | Bacteria | Gammaproteobacteria | ecr | Escherichia coli O8 IAI1 (commensal) | 4351 | 0.518 |
| Eukaryotes | Plants | Green algae | ecre | Chlamydomonas reinhardtii (EST) | 14416 | 0.678 |
| Prokaryotes | Bacteria | Gammaproteobacteria | ecs | Escherichia coli O157:H7 Sakai (EHEC) | 5317 | 0.516 |
| Prokaryotes | Bacteria | Gammaproteobacteria | ect | Escherichia coli O7:K1 IAI39 (ExPEC) | 4730 | 0.516 |
| Eukaryotes | Fungi | Microsporidians | ecu | Encephalitozoon cuniculi | 1996 | 0.475 |
| Prokaryotes | Bacteria | Gammaproteobacteria | ecv | Escherichia coli O1:K1:H7 (APEC) | 4851 | 0.514 |
| Prokaryotes | Bacteria | Gammaproteobacteria | ecw | Escherichia coli O139:H28 E24377A (ETEC) | 4991 | 0.516 |
| Prokaryotes | Bacteria | Gammaproteobacteria | ecx | Escherichia coli O9 HS (commensal) | 4378 | 0.518 |
| Prokaryotes | Bacteria | Gammaproteobacteria | ecy | Escherichia coli O152:H28 SE11 (commensal) | 5002 | 0.517 |
| Prokaryotes | Bacteria | Gammaproteobacteria | ecz | Escherichia coli O45:K1:H7 S88 (ExPEC) | 4827 | 0.517 |
| Eukaryotes | Protists | Amoebozoa | edi | Entamoeba dispar | 8811 | 0.276 |
| Prokaryotes | Bacteria | Firmicutes | eel | Eubacterium eligens | 2765 | 0.381 |
| Prokaryotes | Bacteria | Firmicutes | efa | Enterococcus faecalis | 3264 | 0.380 |
| Prokaryotes | Bacteria | Gammaproteobacteria | efe | Escherichia fergusonii | 4319 | 0.509 |
| Prokaryotes | Bacteria | Firmicutes | eha | Ethanoligenens harbinense | 2701 | 0.565 |
| Eukaryotes | Protists | Amoebozoa | ehi | Entamoeba histolytica | 8162 | 0.280 |
| Prokaryotes | Bacteria | Gammaproteobacteria | eic | Edwardsiella ictaluri | 3784 | 0.587 |
| Prokaryotes | Bacteria | Actinobacteria | ele | Eggerthella lenta | 3070 | 0.648 |
| Prokaryotes | Bacteria | Alphaproteobacteria | eli | Erythrobacter litoralis | 3011 | 0.634 |
| Prokaryotes | Bacteria | Firmicutes | elm | Eubacterium limosum | 4509 | 0.487 |
| Eukaryotes | Plants | Eudicots | emdm | Malus x domestica (apple tree) (EST) | 19166 | 0.497 |
| Prokaryotes | Bacteria | Elusimicrobia | emi | Elusimicrobium minutum | 1529 | 0.408 |
| Prokaryotes | Bacteria | Gammaproteobacteria | enc | Enterobacter cloacae subsp. cloacae ATCC 13047 | 5518 | 0.557 |
| Prokaryotes | Bacteria | Gammaproteobacteria | ent | Enterobacter sp. 638 | 4240 | 0.540 |
| Prokaryotes | Bacteria | Gammaproteobacteria | eoh | Escherichia coli O103:H2 12009 (EHEC) | 5121 | 0.517 |
| Prokaryotes | Bacteria | Gammaproteobacteria | eoi | Escherichia coli O111:H- 11128 (EHEC) | 5407 | 0.515 |
| Prokaryotes | Bacteria | Gammaproteobacteria | eoj | Escherichia coli O26:H11 11368 (EHEC) | 5519 | 0.518 |
| Prokaryotes | Bacteria | Gammaproteobacteria | eok | Escherichia coli O55:H7 CB9615 (EPEC) | 5121 | 0.515 |
| Eukaryotes | Plants | Monocots | eosa | Oryza sativa japonica (Japanese rice) (EST) | 28453 | 0.548 |
| Eukaryotes | Plants | Eudicots | epop | Populus trichocarpa (black cottonwood) (EST) | 40484 | 0.438 |
| Eukaryotes | Plants | Mosses | eppp | Physcomitrella patens subsp. patens (EST) | 35925 | 0.472 |
| Prokaryotes | Bacteria | Gammaproteobacteria | epy | Erwinia pyrifoliae | 3697 | 0.545 |
| Eukaryotes | Plants | Eudicots | ercu | Ricinus communis (castor bean) (EST) | 31258 | 0.448 |
| Prokaryotes | Bacteria | Firmicutes | ere | Eubacterium rectale | 3621 | 0.423 |
| Prokaryotes | Bacteria | Alphaproteobacteria | erg | Ehrlichia ruminantium Gardel | 950 | 0.307 |
| Prokaryotes | Bacteria | Alphaproteobacteria | eru | Ehrlichia ruminantium Welgevonden (South Africa) | 888 | 0.308 |
| Prokaryotes | Bacteria | Alphaproteobacteria | erw | Ehrlichia ruminantium Welgevonden (France) | 958 | 0.307 |
| Prokaryotes | Bacteria | Gammaproteobacteria | esa | Enterobacter sakazakii | 4421 | 0.578 |
| Eukaryotes | Plants | Monocots | esbi | Sorghum bicolor (sorghum) (EST) | 33002 | 0.546 |
| Prokaryotes | Bacteria | Gammaproteobacteria | esc | Enterobacter cloacae SCF1 | 4399 | 0.583 |
| Prokaryotes | Bacteria | Firmicutes | esi | Exiguobacterium sibiricum | 3015 | 0.486 |
| Eukaryotes | Animals | Flatworms | esja | Schistosoma japonicum (fluke) (EST) | 4394 | 0.654 |
| Eukaryotes | Animals | Echinoderms | espu | Strongylocentrotus purpuratus (purple sea urchin) (EST) | 28880 | 0.487 |
| Prokaryotes | Bacteria | Gammaproteobacteria | eta | Erwinia tasmaniensis | 3622 | 0.546 |
| Prokaryotes | Bacteria | Gammaproteobacteria | etr | Edwardsiella tarda | 3589 | 0.608 |
| Prokaryotes | Bacteria | Gammaproteobacteria | etw | Escherichia coli O157:H7 TW14359 (EHEC) | 5365 | 0.515 |
| Prokaryotes | Bacteria | Gammaproteobacteria | eum | Escherichia coli O17:K52:H18 UMN026 (ExPEC) | 5016 | 0.517 |
| Eukaryotes | Plants | Green algae | evcn | Volvox carteri f. nagariensis (EST) | 14434 | 0.630 |
| Eukaryotes | Plants | Eudicots | evvi | Vitis vinifera (wine grape) (EST) | 22511 | 0.451 |
| Eukaryotes | Plants | Monocots | ezma | Zea mays (maize) (EST) | 22152 | 0.573 |
| Prokaryotes | Bacteria | Actinobacteria | fal | Frankia alni | 6707 | 0.730 |
| Prokaryotes | Bacteria | Bacteroidetes | fba | Flavobacteriaceae bacterium | 2534 | 0.435 |
| Prokaryotes | Bacteria | Bacteroidetes | fbc | Maribacter sp. HTCC2170 | 3411 | 0.376 |
| Prokaryotes | Bacteria | Gammaproteobacteria | fbl | Ferrimonas balearica | 3782 | 0.611 |
| Eukaryotes | Fungi | Ascomycetes | fgr | Fusarium graminearum | 11594 | 0.515 |
| Prokaryotes | Bacteria | Bacteroidetes | fjo | Flavobacterium johnsoniae | 5017 | 0.351 |
| Prokaryotes | Bacteria | Firmicutes | fma | Finegoldia magna | 1813 | 0.325 |
| Prokaryotes | Bacteria | Hyperthermophilic bacteria | fno | Fervidobacterium nodosum | 1750 | 0.354 |
| Prokaryotes | Bacteria | Fusobacteria | fnu | Fusobacterium nucleatum | 2063 | 0.277 |
| Prokaryotes | Bacteria | Gammaproteobacteria | fph | Francisella philomiragia | 1915 | 0.332 |
| Prokaryotes | Archaea | Euryarchaeota | fpl | Ferroglobus placidus | 2480 | 0.447 |
| Prokaryotes | Bacteria | Bacteroidetes | fps | Flavobacterium psychrophilum | 2412 | 0.335 |
| Prokaryotes | Bacteria | Actinobacteria | fra | Frankia sp. CcI3 | 4499 | 0.705 |
| Prokaryotes | Bacteria | Actinobacteria | fre | Frankia sp. EAN1pec | 7191 | 0.715 |
| Prokaryotes | Bacteria | Actinobacteria | fri | Frankia sp. EuI1c | 7083 | 0.726 |
| Prokaryotes | Bacteria | Fibrobacteres | fsu | Fibrobacter succinogenes | 3085 | 0.489 |
| Prokaryotes | Bacteria | Gammaproteobacteria | fta | Francisella tularensis subsp. holarctica FTNF002-00 | 1581 | 0.330 |
| Prokaryotes | Bacteria | Bacteroidetes | fte | Fluviicola taffensis | 4033 | 0.370 |
| Prokaryotes | Bacteria | Gammaproteobacteria | ftf | Francisella tularensis subsp. tularensis FSC 198 | 1605 | 0.331 |
| Prokaryotes | Bacteria | Gammaproteobacteria | fth | Francisella tularensis subsp. holarctica OSU18 | 1555 | 0.329 |
| Prokaryotes | Bacteria | Gammaproteobacteria | ftl | Francisella tularensis subsp. holarctica LVS | 1754 | 0.330 |
| Prokaryotes | Bacteria | Gammaproteobacteria | ftm | Francisella tularensis subsp. mediasiatica FSC147 | 1406 | 0.331 |
| Prokaryotes | Bacteria | Gammaproteobacteria | ftn | Francisella novicida U112 | 1719 | 0.333 |
| Prokaryotes | Bacteria | Gammaproteobacteria | ftu | Francisella tularensis subsp. tularensis SCHU S4 | 1604 | 0.331 |
| Prokaryotes | Bacteria | Gammaproteobacteria | ftw | Francisella tularensis subsp. tularensis WY96-3418 | 1634 | 0.331 |
| Prokaryotes | Bacteria | Gammaproteobacteria | gag | Glaciecola agarilytica | 4547 | 0.448 |
| Prokaryotes | Bacteria | Gammaproteobacteria | gan | Gallibacterium anatis | 2500 | 0.407 |
| Prokaryotes | Bacteria | Gemmatimonadetes | gau | Gemmatimonas aurantiaca | 3935 | 0.645 |
| Prokaryotes | Bacteria | Alphaproteobacteria | gbe | Granulibacter bethesdensis | 2437 | 0.596 |
| Prokaryotes | Bacteria | Deltaproteobacteria | gbm | Geobacter bemidjiensis | 4057 | 0.614 |
| Prokaryotes | Bacteria | Actinobacteria | gbr | Gordonia bronchialis | 4696 | 0.673 |
| Prokaryotes | Bacteria | Betaproteobacteria | gca | Gallionella capsiferriformans | 2894 | 0.534 |
| Prokaryotes | Bacteria | Firmicutes | gct | Geobacillus sp. C56-T3 | 3315 | 0.533 |
| Prokaryotes | Bacteria | Alphaproteobacteria | gdi | Gluconacetobacter diazotrophicus PAl 5 (Brazil) | 3852 | 0.668 |
| Prokaryotes | Bacteria | Alphaproteobacteria | gdj | Gluconacetobacter diazotrophicus PAl 5 (JGI) | 3501 | 0.671 |
| Prokaryotes | Bacteria | Deltaproteobacteria | geb | Geobacter sp. M18 | 4434 | 0.623 |
| Prokaryotes | Bacteria | Deltaproteobacteria | gem | Geobacter sp. M21 | 4080 | 0.616 |
| Prokaryotes | Bacteria | Deltaproteobacteria | geo | Geobacter sp. FRC-32 | 3798 | 0.543 |
| Prokaryotes | Bacteria | Bacteroidetes | gfo | Gramella forsetii | 3584 | 0.373 |
| Eukaryotes | Animals | Vertebrates | gga | Gallus gallus (chicken) | 18114 | 0.510 |
| Prokaryotes | Bacteria | Firmicutes | gka | Geobacillus kaustophilus | 3539 | 0.529 |
| Eukaryotes | Protists | Diplomonads | gla | Giardia lamblia | 6502 | 0.493 |
| Prokaryotes | Bacteria | Deltaproteobacteria | glo | Geobacter lovleyi | 3685 | 0.553 |
| Prokaryotes | Bacteria | Firmicutes | gmc | Geobacillus sp. Y4.1MC1 | 3669 | 0.451 |
| Prokaryotes | Bacteria | Deltaproteobacteria | gme | Geobacter metallireducens | 3534 | 0.603 |
| Prokaryotes | Bacteria | Actinobacteria | gob | Geodermatophilus obscurus | 4810 | 0.740 |
| Prokaryotes | Bacteria | Alphaproteobacteria | gox | Gluconobacter oxydans | 2664 | 0.614 |
| Prokaryotes | Bacteria | Gammaproteobacteria | gpb | Gamma proteobacterium HdN1 | 3763 | 0.537 |
| Prokaryotes | Bacteria | Deltaproteobacteria | gsu | Geobacter sulfurreducens | 3427 | 0.616 |
| Prokaryotes | Bacteria | Firmicutes | gtn | Geobacillus thermodenitrificans | 3445 | 0.499 |
| Prokaryotes | Bacteria | Deltaproteobacteria | gur | Geobacter uraniumreducens | 4358 | 0.553 |
| Prokaryotes | Bacteria | Actinobacteria | gva | Gardnerella vaginalis | 1261 | 0.428 |
| Prokaryotes | Bacteria | Actinobacteria | gvg | Gardnerella vaginalis ATCC 14019 | 1365 | 0.422 |
| Prokaryotes | Bacteria | Cyanobacteria | gvi | Gloeobacter violaceus | 4430 | 0.629 |
| Prokaryotes | Bacteria | Firmicutes | gwc | Geobacillus sp. WCH70 | 3168 | 0.437 |
| Prokaryotes | Bacteria | Firmicutes | gya | Geobacillus sp. Y412MC52 | 3459 | 0.532 |
| Prokaryotes | Bacteria | Firmicutes | gyc | Geobacillus sp. Y412MC61 | 3446 | 0.532 |
| Prokaryotes | Bacteria | Firmicutes | gym | Geobacillus sp. Y412MC10 | 6239 | 0.525 |
| Prokaryotes | Bacteria | Epsilonproteobacteria | hac | Helicobacter acinonychis | 1619 | 0.389 |
| Prokaryotes | Archaea | Euryarchaeota | hal | Halobacterium sp. NRC-1 | 2622 | 0.669 |
| Prokaryotes | Bacteria | Gammaproteobacteria | hap | Haemophilus parasuis | 2021 | 0.410 |
| Prokaryotes | Bacteria | Betaproteobacteria | har | Herminiimonas arsenicoxydans | 3295 | 0.552 |
| Prokaryotes | Bacteria | Firmicutes | has | Halanaerobium sp. sapolanicus | 2295 | 0.339 |
| Prokaryotes | Bacteria | Green nonsulfur bacteria | hau | Herpetosiphon aurantiacus | 5279 | 0.514 |
| Prokaryotes | Bacteria | Alphaproteobacteria | hba | Hirschia baltica | 3187 | 0.458 |
| Prokaryotes | Archaea | Euryarchaeota | hbo | Halogeometricum borinquense | 3898 | 0.610 |
| Prokaryotes | Archaea | Crenarchaeota | hbu | Hyperthermus butylicus | 1603 | 0.541 |
| Prokaryotes | Bacteria | Gammaproteobacteria | hch | Hahella chejuensis | 6778 | 0.548 |
| Prokaryotes | Bacteria | Alphaproteobacteria | hci | Candidatus Hodgkinia cicadicola | 169 | 0.585 |
| Prokaryotes | Bacteria | Gammaproteobacteria | hde | Candidatus Hamiltonella defensa | 2148 | 0.414 |
| Prokaryotes | Bacteria | Alphaproteobacteria | hdn | Hyphomicrobium denitrificans | 3512 | 0.613 |
| Prokaryotes | Bacteria | Gammaproteobacteria | hdu | Haemophilus ducreyi | 1717 | 0.392 |
| Prokaryotes | Bacteria | Gammaproteobacteria | hel | Halomonas elongata | 3474 | 0.647 |
| Prokaryotes | Bacteria | Epsilonproteobacteria | hfe | Helicobacter felis | 1668 | 0.449 |
| Prokaryotes | Bacteria | Gammaproteobacteria | hha | Halorhodospira halophila | 2407 | 0.682 |
| Prokaryotes | Bacteria | Epsilonproteobacteria | hhe | Helicobacter hepaticus | 1876 | 0.364 |
| Prokaryotes | Bacteria | Gammaproteobacteria | hif | Haemophilus influenzae F3031 | 1770 | 0.391 |
| Prokaryotes | Bacteria | Gammaproteobacteria | hil | Haemophilus influenzae F3047 | 1786 | 0.391 |
| Prokaryotes | Bacteria | Gammaproteobacteria | hin | Haemophilus influenzae Rd KW20 (serotype d) | 1657 | 0.390 |
| Prokaryotes | Bacteria | Gammaproteobacteria | hip | Haemophilus influenzae PittEE | 1613 | 0.390 |
| Prokaryotes | Bacteria | Gammaproteobacteria | hiq | Haemophilus influenzae PittGG | 1661 | 0.388 |
| Prokaryotes | Bacteria | Gammaproteobacteria | hit | Haemophilus influenzae 86-028NP (nontypeable) | 1792 | 0.390 |
| Prokaryotes | Archaea | Euryarchaeota | hje | Halalkalicoccus jeotgali | 3873 | 0.642 |
| Prokaryotes | Archaea | Euryarchaeota | hla | Halorubrum lacusprofundi | 3560 | 0.650 |
| Prokaryotes | Archaea | Euryarchaeota | hma | Haloarcula marismortui | 4243 | 0.622 |
| Eukaryotes | Animals | Cnidarians | hmg | Hydra magnipapillata | 17250 | 0.346 |
| Prokaryotes | Bacteria | Firmicutes | hmo | Heliobacterium modesticaldum | 3000 | 0.580 |
| Prokaryotes | Bacteria | Deltaproteobacteria | hmr | Hippea maritima | 1677 | 0.376 |
| Prokaryotes | Bacteria | Epsilonproteobacteria | hms | Helicobacter mustelae | 1398 | 0.429 |
| Prokaryotes | Archaea | Euryarchaeota | hmu | Halomicrobium mukohataei | 3349 | 0.665 |
| Prokaryotes | Bacteria | Gammaproteobacteria | hna | Halothiobacillus neapolitanus | 2357 | 0.555 |
| Prokaryotes | Bacteria | Alphaproteobacteria | hne | Hyphomonas neptunium | 3505 | 0.623 |
| Prokaryotes | Bacteria | Deltaproteobacteria | hoh | Haliangium ochraceum | 6719 | 0.699 |
| Prokaryotes | Bacteria | Firmicutes | hor | Halothermothrix orenii | 2342 | 0.390 |
| Prokaryotes | Bacteria | Epsilonproteobacteria | hpa | Helicobacter pylori HPAG1 | 1539 | 0.398 |
| Prokaryotes | Bacteria | Epsilonproteobacteria | hpb | Helicobacter pylori B38 | 1382 | 0.399 |
| Prokaryotes | Bacteria | Epsilonproteobacteria | hpc | Helicobacter pylori PeCan4 | 1563 | 0.397 |
| Prokaryotes | Bacteria | Epsilonproteobacteria | hpg | Helicobacter pylori G27 | 1504 | 0.396 |
| Prokaryotes | Bacteria | Epsilonproteobacteria | hpj | Helicobacter pylori J99 | 1488 | 0.400 |
| Prokaryotes | Bacteria | Epsilonproteobacteria | hpl | Helicobacter pylori B8 | 1707 | 0.394 |
| Prokaryotes | Bacteria | Epsilonproteobacteria | hpm | Helicobacter pylori SJM180 | 1581 | 0.397 |
| Prokaryotes | Bacteria | Epsilonproteobacteria | hpp | Helicobacter pylori P12 | 1578 | 0.395 |
| Prokaryotes | Bacteria | Epsilonproteobacteria | hps | Helicobacter pylori Shi470 | 1568 | 0.396 |
| Prokaryotes | Bacteria | Epsilonproteobacteria | hpy | Helicobacter pylori 26695 | 1573 | 0.396 |
| Eukaryotes | Animals | Vertebrates | hsa | Homo sapiens (human) | 20846 | 0.524 |
| Prokaryotes | Bacteria | Betaproteobacteria | hse | Herbaspirillum seropedicae | 4735 | 0.642 |
| Prokaryotes | Archaea | Euryarchaeota | hsl | Halobacterium salinarum R1 | 2749 | 0.665 |
| Prokaryotes | Bacteria | Gammaproteobacteria | hsm | Haemophilus somnus 2336 | 1980 | 0.381 |
| Prokaryotes | Bacteria | Gammaproteobacteria | hso | Haemophilus somnus 129PT | 1798 | 0.379 |
| Prokaryotes | Bacteria | Hyperthermophilic bacteria | hth | Hydrogenobacter thermophilus | 1893 | 0.442 |
| Prokaryotes | Archaea | Euryarchaeota | htu | Haloterrigena turkmenica | 5113 | 0.656 |
| Prokaryotes | Archaea | Euryarchaeota | hut | Halorhabdus utahensis | 2998 | 0.637 |
| Prokaryotes | Archaea | Euryarchaeota | hvo | Haloferax volcanii | 4015 | 0.664 |
| Prokaryotes | Archaea | Euryarchaeota | hwa | Haloquadratum walsbyi | 2646 | 0.490 |
| Prokaryotes | Bacteria | Hyperthermophilic bacteria | hya | Hydrogenobaculum sp. Y04AAS1 | 1629 | 0.351 |
| Prokaryotes | Archaea | Crenarchaeota | iag | Ignisphaera aggregans | 1930 | 0.362 |
| Prokaryotes | Bacteria | Actinobacteria | ica | Intrasporangium calvum | 3563 | 0.708 |
| Prokaryotes | Archaea | Crenarchaeota | iho | Ignicoccus hospitalis | 1434 | 0.566 |
| Prokaryotes | Bacteria | Gammaproteobacteria | ilo | Idiomarina loihiensis | 2628 | 0.475 |
| Prokaryotes | Bacteria | Planctomycetes | ipa | Isosphaera pallida | 3722 | 0.630 |
| Prokaryotes | Bacteria | Fusobacteria | ipo | Ilyobacter polytropus | 2880 | 0.355 |
| Eukaryotes | Animals | Arthropods | isc | Ixodes scapularis (black-legged tick) | 20467 | 0.582 |
| Prokaryotes | Bacteria | Alphaproteobacteria | jan | Jannaschia sp. CCS1 | 4283 | 0.626 |
| Prokaryotes | Bacteria | Actinobacteria | jde | Jonesia denitrificans | 2511 | 0.585 |
| Prokaryotes | Archaea | Korarchaeota | kcr | Candidatus Korarchaeum cryptofilum | 1603 | 0.496 |
| Prokaryotes | Bacteria | Bacteroidetes | kdi | Krokinobacter diaphorus | 2978 | 0.379 |
| Prokaryotes | Bacteria | Actinobacteria | kfl | Kribbella flavida | 6943 | 0.708 |
| Prokaryotes | Bacteria | Gammaproteobacteria | kko | Kangiella koreensis | 2632 | 0.443 |
| Eukaryotes | Fungi | Ascomycetes | kla | Kluyveromyces lactis | 5084 | 0.401 |
| Prokaryotes | Bacteria | Hyperthermophilic bacteria | kol | Kosmotoga olearia | 2118 | 0.416 |
| Prokaryotes | Bacteria | Gammaproteobacteria | kpe | Klebsiella pneumoniae 342 | 5768 | 0.580 |
| Prokaryotes | Bacteria | Gammaproteobacteria | kpn | Klebsiella pneumoniae | 5185 | 0.584 |
| Prokaryotes | Bacteria | Gammaproteobacteria | kpu | Klebsiella pneumoniae NTUH-K2044 | 5262 | 0.584 |
| Prokaryotes | Bacteria | Actinobacteria | kra | Kineococcus radiotolerans | 4681 | 0.742 |
| Prokaryotes | Bacteria | Actinobacteria | krh | Kocuria rhizophila | 2357 | 0.710 |
| Prokaryotes | Bacteria | Actinobacteria | kse | Kytococcus sedentarius | 2554 | 0.717 |
| Prokaryotes | Bacteria | Gammaproteobacteria | kva | Klebsiella variicola | 5057 | 0.587 |
| Prokaryotes | Bacteria | Alphaproteobacteria | kvu | Ketogulonicigenium vulgare | 3213 | 0.623 |
| Prokaryotes | Bacteria | Firmicutes | lac | Lactobacillus acidophilus NCFM | 1864 | 0.354 |
| Prokaryotes | Bacteria | Firmicutes | lai | Lactobacillus acidophilus 30SC | 2059 | 0.390 |
| Prokaryotes | Bacteria | Firmicutes | lam | Lactobacillus amylovorus | 2054 | 0.391 |
| Prokaryotes | Bacteria | Alphaproteobacteria | las | Candidatus Liberibacter asiaticus | 1109 | 0.378 |
| Prokaryotes | Bacteria | Fusobacteria | lba | Leptotrichia buccalis | 2220 | 0.310 |
| Eukaryotes | Fungi | Basidiomycetes | lbc | Laccaria bicolor | 18215 | 0.506 |
| Prokaryotes | Bacteria | Spirochaetes | lbf | Leptospira biflexa serovar Patoc Patoc 1 (Ames) | 3600 | 0.390 |
| Prokaryotes | Bacteria | Firmicutes | lbh | Lactobacillus buchneri | 2392 | 0.452 |
| Prokaryotes | Bacteria | Spirochaetes | lbi | Leptospira biflexa serovar Patoc Patoc 1 (Paris) | 3726 | 0.390 |
| Prokaryotes | Bacteria | Spirochaetes | lbj | Leptospira borgpetersenii JB197 | 2880 | 0.412 |
| Prokaryotes | Bacteria | Spirochaetes | lbl | Leptospira borgpetersenii L550 | 2945 | 0.412 |
| Prokaryotes | Bacteria | Firmicutes | lbr | Lactobacillus brevis | 2218 | 0.471 |
| Prokaryotes | Bacteria | Firmicutes | lbu | Lactobacillus delbrueckii ATCC BAA-365 | 1715 | 0.514 |
| Prokaryotes | Bacteria | Bacteroidetes | lby | Leadbetterella byssophila | 3465 | 0.410 |
| Eukaryotes | Protists | Euglenozoa | lbz | Leishmania braziliensis | 7895 | 0.604 |
| Prokaryotes | Bacteria | Firmicutes | lca | Lactobacillus casei ATCC 334 | 2768 | 0.473 |
| Prokaryotes | Bacteria | Firmicutes | lcb | Lactobacillus casei BL23 | 3015 | 0.471 |
| Prokaryotes | Bacteria | Betaproteobacteria | lch | Leptothrix cholodnii | 4363 | 0.693 |
| Prokaryotes | Bacteria | Firmicutes | lci | Leuconostoc citreum | 1820 | 0.396 |
| Prokaryotes | Bacteria | Firmicutes | lcr | Lactobacillus crispatus | 2024 | 0.378 |
| Prokaryotes | Bacteria | Firmicutes | lcz | Lactobacillus casei Zhang | 2848 | 0.471 |
| Prokaryotes | Bacteria | Firmicutes | ldb | Lactobacillus delbrueckii ATCC 11842 | 1529 | 0.514 |
| Prokaryotes | Bacteria | Firmicutes | lde | Lactobacillus delbrueckii subsp. bulgaricus ND02 | 2018 | 0.512 |
| Eukaryotes | Fungi | Ascomycetes | lel | Lodderomyces elongisporus | 5799 | 0.404 |
| Prokaryotes | Bacteria | Firmicutes | lfe | Lactobacillus fermentum | 1843 | 0.530 |
| Prokaryotes | Bacteria | Firmicutes | lga | Lactobacillus gasseri | 1755 | 0.358 |
| Prokaryotes | Bacteria | Firmicutes | lgs | Leuconostoc gasicomitatum | 1912 | 0.375 |
| Prokaryotes | Bacteria | Firmicutes | lhe | Lactobacillus helveticus | 1610 | 0.379 |
| Prokaryotes | Bacteria | Betaproteobacteria | lhk | Laribacter hongkongensis | 3235 | 0.631 |
| Prokaryotes | Bacteria | Spirochaetes | lic | Leptospira interrogans serovar Copenhageni | 3667 | 0.367 |
| Eukaryotes | Protists | Euglenozoa | lif | Leishmania infantum | 7992 | 0.624 |
| Prokaryotes | Bacteria | Spirochaetes | lil | Leptospira interrogans serovar lai | 3702 | 0.367 |
| Prokaryotes | Bacteria | Firmicutes | lin | Listeria innocua | 3043 | 0.380 |
| Prokaryotes | Bacteria | Deltaproteobacteria | lip | Lawsonia intracellularis | 1340 | 0.348 |
| Prokaryotes | Bacteria | Firmicutes | ljf | Lactobacillus johnsonii FI9785 | 1735 | 0.351 |
| Prokaryotes | Bacteria | Firmicutes | ljo | Lactobacillus johnsonii NCC 533 | 1821 | 0.353 |
| Prokaryotes | Bacteria | Firmicutes | lki | Leuconostoc kimchii | 2129 | 0.387 |
| Prokaryotes | Bacteria | Firmicutes | lla | Lactococcus lactis subsp. lactis IL1403 | 2321 | 0.364 |
| Prokaryotes | Bacteria | Firmicutes | llc | Lactococcus lactis subsp. cremoris SK11 | 2504 | 0.369 |
| Prokaryotes | Bacteria | Firmicutes | llk | Lactococcus lactis subsp. lactis KF147 | 2473 | 0.359 |
| Prokaryotes | Bacteria | Firmicutes | llm | Lactococcus lactis subsp. cremoris MG1363 | 2434 | 0.369 |
| Prokaryotes | Bacteria | Gammaproteobacteria | llo | Legionella longbeachae | 3470 | 0.378 |
| Eukaryotes | Protists | Euglenozoa | lma | Leishmania major | 8265 | 0.625 |
| Prokaryotes | Bacteria | Firmicutes | lmc | Listeria monocytogenes Clip81459 | 2766 | 0.387 |
| Prokaryotes | Bacteria | Firmicutes | lme | Leuconostoc mesenteroides | 2005 | 0.385 |
| Prokaryotes | Bacteria | Firmicutes | lmf | Listeria monocytogenes F2365 | 2821 | 0.387 |
| Prokaryotes | Bacteria | Firmicutes | lmh | Listeria monocytogenes HCC23 | 2974 | 0.389 |
| Prokaryotes | Bacteria | Firmicutes | lmn | Listeria monocytogenes 08-5578 | 3088 | 0.385 |
| Prokaryotes | Bacteria | Firmicutes | lmo | Listeria monocytogenes EGD-e | 2846 | 0.386 |
| Prokaryotes | Bacteria | Firmicutes | lmy | Listeria monocytogenes 08-5923 | 2966 | 0.386 |
| Prokaryotes | Bacteria | Gammaproteobacteria | lpa | Legionella pneumophila 2300/99 Alcoy | 3190 | 0.391 |
| Prokaryotes | Bacteria | Gammaproteobacteria | lpc | Legionella pneumophila Corby | 3204 | 0.392 |
| Prokaryotes | Bacteria | Gammaproteobacteria | lpf | Legionella pneumophila Lens | 2934 | 0.392 |
| Prokaryotes | Bacteria | Firmicutes | lpj | Lactobacillus plantarum JDM1 | 2948 | 0.458 |
| Prokaryotes | Bacteria | Firmicutes | lpl | Lactobacillus plantarum WCFS1 | 3057 | 0.455 |
| Prokaryotes | Bacteria | Gammaproteobacteria | lpn | Legionella pneumophila Philadelphia 1 | 2943 | 0.390 |
| Prokaryotes | Bacteria | Gammaproteobacteria | lpp | Legionella pneumophila Paris | 3166 | 0.391 |
| Prokaryotes | Bacteria | Firmicutes | lps | Lactobacillus plantarum subsp. plantarum ST-III | 3038 | 0.457 |
| Prokaryotes | Bacteria | Firmicutes | lre | Lactobacillus reuteri DSM 20016 | 1900 | 0.397 |
| Prokaryotes | Bacteria | Firmicutes | lrf | Lactobacillus reuteri JCM 1112 | 1820 | 0.398 |
| Prokaryotes | Bacteria | Firmicutes | lrh | Lactobacillus rhamnosus GG | 2913 | 0.475 |
| Prokaryotes | Bacteria | Firmicutes | lrl | Lactobacillus rhamnosus Lc 705 | 2957 | 0.474 |
| Prokaryotes | Bacteria | Firmicutes | lsa | Lactobacillus sakei | 1879 | 0.421 |
| Prokaryotes | Bacteria | Firmicutes | lsg | Listeria seeligeri | 2710 | 0.380 |
| Prokaryotes | Bacteria | Firmicutes | lsl | Lactobacillus salivarius | 2013 | 0.337 |
| Prokaryotes | Bacteria | Alphaproteobacteria | lso | Candidatus Liberibacter solanacearum | 1192 | 0.368 |
| Prokaryotes | Bacteria | Firmicutes | lsp | Lysinibacillus sphaericus | 4771 | 0.380 |
| Eukaryotes | Fungi | Ascomycetes | lth | Lachancea thermotolerans | 5091 | 0.482 |
| Prokaryotes | Bacteria | Firmicutes | lwe | Listeria welshimeri SLCC5334 | 2774 | 0.370 |
| Prokaryotes | Bacteria | Actinobacteria | lxx | Leifsonia xyli xyli CTCB07 | 2030 | 0.684 |
| Prokaryotes | Bacteria | Tenericutes | maa | Mycoplasma agalactiae PG2 | 742 | 0.304 |
| Prokaryotes | Bacteria | Actinobacteria | mab | Mycobacterium abscessus ATCC 19977 | 4941 | 0.645 |
| Prokaryotes | Archaea | Euryarchaeota | mac | Methanosarcina acetivorans | 4540 | 0.452 |
| Prokaryotes | Archaea | Euryarchaeota | mae | Methanococcus aeolicus | 1490 | 0.315 |
| Prokaryotes | Bacteria | Alphaproteobacteria | mag | Magnetospirillum magneticum | 4559 | 0.661 |
| Prokaryotes | Bacteria | Tenericutes | mal | Mycoplasma agalactiae 5632 | 813 | 0.303 |
| Prokaryotes | Bacteria | Gammaproteobacteria | maq | Marinobacter aquaeolei | 4272 | 0.576 |
| Prokaryotes | Bacteria | Cyanobacteria | mar | Microcystis aeruginosa | 6312 | 0.429 |
| Prokaryotes | Bacteria | Tenericutes | mat | Mycoplasma arthritidis | 631 | 0.315 |
| Prokaryotes | Bacteria | Actinobacteria | mau | Micromonospora aurantiaca | 6222 | 0.731 |
| Prokaryotes | Bacteria | Actinobacteria | mav | Mycobacterium avium 104 | 5120 | 0.693 |
| Prokaryotes | Archaea | Euryarchaeota | mba | Methanosarcina barkeri | 3624 | 0.422 |
| Prokaryotes | Bacteria | Actinobacteria | mbb | Mycobacterium bovis BCG Pasteur 1173P2 | 3949 | 0.659 |
| Prokaryotes | Archaea | Euryarchaeota | mbn | Candidatus Methanoregula boonei | 2452 | 0.556 |
| Prokaryotes | Bacteria | Actinobacteria | mbo | Mycobacterium bovis AF2122/97 | 3918 | 0.659 |
| Eukaryotes | Protists | Choanoflagellates | mbr | Monosiga brevicollis | 9171 | 0.572 |
| Prokaryotes | Bacteria | Actinobacteria | mbt | Mycobacterium bovis BCG Tokyo 172 | 3944 | 0.659 |
| Prokaryotes | Archaea | Euryarchaeota | mbu | Methanococcoides burtonii | 2273 | 0.421 |
| Prokaryotes | Bacteria | Tenericutes | mbv | Mycoplasma bovis PG45 | 765 | 0.300 |
| Prokaryotes | Bacteria | Gammaproteobacteria | mca | Methylococcus capsulatus | 2956 | 0.639 |
| Eukaryotes | Animals | Vertebrates | mcc | Macaca mulatta (rhesus monkey) | 22640 | 0.522 |
| Prokaryotes | Bacteria | Tenericutes | mcd | Mycoplasma crocodyli | 689 | 0.276 |
| Prokaryotes | Bacteria | Alphaproteobacteria | mch | Methylobacterium chloromethanicum | 5516 | 0.688 |
| Prokaryotes | Bacteria | Alphaproteobacteria | mci | Mesorhizobium ciceri | 6264 | 0.632 |
| Prokaryotes | Archaea | Euryarchaeota | mcj | Methanosaeta concilii | 2850 | 0.523 |
| Prokaryotes | Bacteria | Firmicutes | mcl | Macrococcus caseolyticus | 2052 | 0.374 |
| Prokaryotes | Archaea | Crenarchaeota | mcn | Metallosphaera cuprina | 2029 | 0.429 |
| Prokaryotes | Bacteria | Tenericutes | mco | Mycoplasma conjunctivae | 692 | 0.292 |
| Prokaryotes | Bacteria | Tenericutes | mcp | Mycoplasma capricolum | 812 | 0.243 |
| Prokaryotes | Bacteria | Gammaproteobacteria | mct | Moraxella catarrhalis | 1886 | 0.430 |
| Prokaryotes | Bacteria | Actinobacteria | mcu | Mobiluncus curtisii | 1909 | 0.559 |
| Prokaryotes | Bacteria | Alphaproteobacteria | mdi | Methylobacterium extorquens DM4 | 5736 | 0.688 |
| Eukaryotes | Animals | Vertebrates | mdo | Monodelphis domestica (opossum) | 19166 | 0.497 |
| Prokaryotes | Bacteria | Alphaproteobacteria | mea | Methylobacterium extorquens AM1 | 6212 | 0.694 |
| Prokaryotes | Bacteria | Betaproteobacteria | meh | Methylotenera sp. 301 | 2764 | 0.436 |
| Prokaryotes | Bacteria | Betaproteobacteria | mei | Methylovorus sp. SIP3-4 | 2909 | 0.554 |
| Prokaryotes | Archaea | Euryarchaeota | mel | Methanobacterium sp. AL-21 | 2493 | 0.378 |
| Prokaryotes | Archaea | Euryarchaeota | mem | Methanoculleus marisnigri | 2490 | 0.629 |
| Prokaryotes | Bacteria | Betaproteobacteria | mep | Methylovorus sp. MP688 | 2712 | 0.563 |
| Prokaryotes | Bacteria | Alphaproteobacteria | mes | Mesorhizobium sp. BNC1 | 4543 | 0.616 |
| Prokaryotes | Bacteria | Alphaproteobacteria | met | Methylobacterium sp. 4-46 | 6692 | 0.719 |
| Prokaryotes | Archaea | Euryarchaeota | mev | Methanohalobium evestigatum | 2254 | 0.377 |
| Prokaryotes | Bacteria | Alphaproteobacteria | mex | Methylobacterium extorquens | 4829 | 0.690 |
| Prokaryotes | Bacteria | Betaproteobacteria | mfa | Methylobacillus flagellatus | 2753 | 0.565 |
| Prokaryotes | Archaea | Euryarchaeota | mfe | Methanocaldococcus fervens | 1581 | 0.329 |
| Prokaryotes | Bacteria | Tenericutes | mfl | Mesoplasma florum | 682 | 0.274 |
| Prokaryotes | Bacteria | Tenericutes | mfm | Mycoplasma fermentans M64 | 1050 | 0.274 |
| Prokaryotes | Bacteria | Tenericutes | mfr | Mycoplasma fermentans JER | 797 | 0.277 |
| Prokaryotes | Archaea | Euryarchaeota | mfs | Methanocaldococcus sp. FS406-22 | 1816 | 0.327 |
| Prokaryotes | Archaea | Euryarchaeota | mfv | Methanothermus fervidus | 1283 | 0.320 |
| Prokaryotes | Bacteria | Tenericutes | mga | Mycoplasma gallisepticum | 763 | 0.322 |
| Prokaryotes | Bacteria | Tenericutes | mge | Mycoplasma genitalium | 475 | 0.318 |
| Prokaryotes | Bacteria | Actinobacteria | mgi | Mycobacterium gilvum | 5579 | 0.681 |
| Eukaryotes | Fungi | Basidiomycetes | mgl | Malassezia globosa | 4286 | 0.533 |
| Prokaryotes | Bacteria | Other proteobacteria | mgm | Magnetococcus sp. MC-1 | 3716 | 0.548 |
| Eukaryotes | Fungi | Ascomycetes | mgr | Magnaporthe oryzae | 14010 | 0.577 |
| Prokaryotes | Bacteria | Tenericutes | mha | Mycoplasma haemofelis | 1545 | 0.390 |
| Prokaryotes | Bacteria | Deinococcus | mhd | Marinithermus hydrothermalis | 2205 | 0.684 |
| Prokaryotes | Bacteria | Tenericutes | mhj | Mycoplasma hyopneumoniae J | 657 | 0.295 |
| Prokaryotes | Bacteria | Tenericutes | mho | Mycoplasma hominis | 523 | 0.278 |
| Prokaryotes | Bacteria | Tenericutes | mhp | Mycoplasma hyopneumoniae 7448 | 657 | 0.295 |
| Prokaryotes | Bacteria | Tenericutes | mhr | Mycoplasma hyorhinis | 654 | 0.266 |
| Prokaryotes | Archaea | Euryarchaeota | mhu | Methanospirillum hungatei | 3139 | 0.461 |
| Prokaryotes | Bacteria | Tenericutes | mhy | Mycoplasma hyopneumoniae 232 | 691 | 0.295 |
| Prokaryotes | Archaea | Euryarchaeota | mif | Methanocaldococcus infernus | 1441 | 0.340 |
| Prokaryotes | Bacteria | Actinobacteria | mil | Micromonospora sp. L5 | 6150 | 0.731 |
| Prokaryotes | Bacteria | Verrucomicrobia | min | Methylacidiphilum infernorum | 2472 | 0.459 |
| Prokaryotes | Archaea | Euryarchaeota | mja | Methanocaldococcus jannaschii | 1771 | 0.321 |
| Prokaryotes | Bacteria | Actinobacteria | mjl | Mycobacterium sp. JLS | 5739 | 0.687 |
| Prokaryotes | Archaea | Euryarchaeota | mka | Methanopyrus kandleri | 1687 | 0.612 |
| Prokaryotes | Bacteria | Actinobacteria | mkm | Mycobacterium sp. KMS | 5975 | 0.686 |
| Prokaryotes | Archaea | Euryarchaeota | mla | Methanocorpusculum labreanum | 1741 | 0.511 |
| Prokaryotes | Bacteria | Actinobacteria | mlb | Mycobacterium leprae Br4923 | 1604 | 0.588 |
| Prokaryotes | Bacteria | Tenericutes | mlc | Mycoplasma leachii | 882 | 0.243 |
| Prokaryotes | Bacteria | Actinobacteria | mle | Mycobacterium leprae TN | 1605 | 0.588 |
| Prokaryotes | Bacteria | Alphaproteobacteria | mlo | Mesorhizobium loti | 7272 | 0.632 |
| Prokaryotes | Bacteria | Actinobacteria | mlu | Micrococcus luteus | 2236 | 0.730 |
| Prokaryotes | Archaea | Euryarchaeota | mma | Methanosarcina mazei | 3368 | 0.443 |
| Prokaryotes | Bacteria | Betaproteobacteria | mmb | Methylotenera mobilis | 2338 | 0.465 |
| Prokaryotes | Bacteria | Actinobacteria | mmc | Mycobacterium sp. MCS | 5615 | 0.687 |
| Prokaryotes | Bacteria | Gammaproteobacteria | mme | Marinomonas mediterranea | 4121 | 0.449 |
| Prokaryotes | Archaea | Euryarchaeota | mmg | Methanothermobacter marburgensis | 1757 | 0.498 |
| Prokaryotes | Archaea | Euryarchaeota | mmh | Methanohalophilus mahii | 1987 | 0.437 |
| Prokaryotes | Bacteria | Actinobacteria | mmi | Mycobacterium marinum M | 5452 | 0.661 |
| Prokaryotes | Bacteria | Tenericutes | mml | Mycoplasma mycoides subsp. capri LC 95010 | 922 | 0.242 |
| Prokaryotes | Bacteria | Tenericutes | mmo | Mycoplasma mobile | 633 | 0.256 |
| Prokaryotes | Archaea | Euryarchaeota | mmp | Methanococcus maripaludis S2 | 1722 | 0.342 |
| Prokaryotes | Archaea | Euryarchaeota | mmq | Methanococcus maripaludis C5 | 1822 | 0.341 |
| Prokaryotes | Bacteria | Alphaproteobacteria | mmr | Maricaulis maris | 3063 | 0.630 |
| Prokaryotes | Bacteria | Betaproteobacteria | mms | Minibacterium massiliensis | 3697 | 0.551 |
| Eukaryotes | Animals | Vertebrates | mmu | Mus musculus (mouse) | 23227 | 0.516 |
| Prokaryotes | Bacteria | Gammaproteobacteria | mmw | Marinomonas sp. MWYL1 | 4439 | 0.434 |
| Prokaryotes | Archaea | Euryarchaeota | mmx | Methanococcus maripaludis C6 | 1826 | 0.346 |
| Prokaryotes | Bacteria | Tenericutes | mmy | Mycoplasma mycoides subsp. mycoides SC PG1 | 1017 | 0.244 |
| Prokaryotes | Archaea | Euryarchaeota | mmz | Methanococcus maripaludis C7 | 1788 | 0.344 |
| Prokaryotes | Bacteria | Alphaproteobacteria | mno | Methylobacterium nodulans | 8308 | 0.691 |
| Prokaryotes | Bacteria | Actinobacteria | mpa | Mycobacterium avium paratuberculosis | 4350 | 0.696 |
| Prokaryotes | Archaea | Euryarchaeota | mpd | Methanocella paludicola | 3004 | 0.570 |
| Prokaryotes | Bacteria | Tenericutes | mpe | Mycoplasma penetrans | 1037 | 0.266 |
| Prokaryotes | Archaea | Euryarchaeota | mpi | Methanoplanus petrolearius | 2785 | 0.486 |
| Prokaryotes | Archaea | Euryarchaeota | mpl | Candidatus Methanosphaerula palustris | 2655 | 0.567 |
| Prokaryotes | Bacteria | Tenericutes | mpn | Mycoplasma pneumoniae | 689 | 0.408 |
| Prokaryotes | Bacteria | Alphaproteobacteria | mpo | Methylobacterium populi | 5365 | 0.699 |
| Eukaryotes | Fungi | Basidiomycetes | mpr | Moniliophthora perniciosa | 13560 | 0.498 |
| Prokaryotes | Bacteria | Betaproteobacteria | mpt | Methylibium petroleiphilum | 4449 | 0.689 |
| Prokaryotes | Bacteria | Tenericutes | mpu | Mycoplasma pulmonis | 782 | 0.275 |
| Prokaryotes | Bacteria | Actinobacteria | mra | Mycobacterium tuberculosis H37Ra | 4034 | 0.658 |
| Prokaryotes | Bacteria | Deinococcus | mrb | Meiothermus ruber | 3014 | 0.641 |
| Prokaryotes | Bacteria | Alphaproteobacteria | mrd | Methylobacterium radiotolerans | 6431 | 0.714 |
| Prokaryotes | Archaea | Euryarchaeota | mru | Methanobrevibacter ruminantium | 2217 | 0.365 |
| Prokaryotes | Archaea | Crenarchaeota | mse | Metallosphaera sedula | 2256 | 0.473 |
| Prokaryotes | Archaea | Euryarchaeota | msi | Methanobrevibacter smithii ATCC 35061 | 1793 | 0.322 |
| Prokaryotes | Bacteria | Tenericutes | msk | Mycoplasma suis KI3806 | 794 | 0.316 |
| Prokaryotes | Bacteria | Alphaproteobacteria | msl | Methylocella silvestris | 3818 | 0.637 |
| Prokaryotes | Bacteria | Actinobacteria | msm | Mycobacterium smegmatis | 6717 | 0.678 |
| Prokaryotes | Bacteria | Actinobacteria | msp | Mycobacterium sp. Spyr1 | 5349 | 0.681 |
| Prokaryotes | Bacteria | Tenericutes | mss | Mycoplasma suis Illinois | 844 | 0.316 |
| Prokaryotes | Archaea | Euryarchaeota | mst | Methanosphaera stadtmanae | 1535 | 0.294 |
| Prokaryotes | Bacteria | Gammaproteobacteria | msu | Mannheimia succiniciproducens | 2369 | 0.436 |
| Prokaryotes | Bacteria | Deinococcus | msv | Meiothermus silvanus | 3505 | 0.633 |
| Prokaryotes | Bacteria | Tenericutes | msy | Mycoplasma synoviae | 659 | 0.290 |
| Prokaryotes | Bacteria | Firmicutes | mta | Moorella thermoacetica | 2463 | 0.569 |
| Prokaryotes | Bacteria | Actinobacteria | mtb | Mycobacterium tuberculosis KZN 1435 | 4059 | 0.659 |
| Prokaryotes | Bacteria | Actinobacteria | mtc | Mycobacterium tuberculosis CDC1551 | 4189 | 0.658 |
| Prokaryotes | Bacteria | Actinobacteria | mtf | Mycobacterium tuberculosis F11 | 3941 | 0.659 |
| Prokaryotes | Archaea | Euryarchaeota | mth | Methanothermobacter thermautotrophicus | 1873 | 0.506 |
| Prokaryotes | Archaea | Euryarchaeota | mtp | Methanosaeta thermophila | 1696 | 0.547 |
| Prokaryotes | Bacteria | Actinobacteria | mts | Microbacterium testaceum | 3676 | 0.703 |
| Prokaryotes | Bacteria | Bacteroidetes | mtt | Marivirga tractuosa | 3757 | 0.361 |
| Prokaryotes | Bacteria | Actinobacteria | mtu | Mycobacterium tuberculosis H37Rv | 3988 | 0.659 |
| Prokaryotes | Bacteria | Actinobacteria | mul | Mycobacterium ulcerans | 4241 | 0.656 |
| Prokaryotes | Bacteria | Actinobacteria | mva | Mycobacterium vanbaalenii | 5979 | 0.681 |
| Prokaryotes | Archaea | Euryarchaeota | mvn | Methanococcus vannielii | 1678 | 0.326 |
| Prokaryotes | Archaea | Euryarchaeota | mvo | Methanococcus voltae | 1717 | 0.315 |
| Prokaryotes | Archaea | Euryarchaeota | mvu | Methanocaldococcus vulcanius | 1742 | 0.330 |
| Prokaryotes | Bacteria | Deltaproteobacteria | mxa | Myxococcus xanthus | 7316 | 0.691 |
| Prokaryotes | Bacteria | Epsilonproteobacteria | nam | Nautilia profundicola | 1730 | 0.339 |
| Prokaryotes | Bacteria | Alphaproteobacteria | nar | Novosphingobium aromaticivorans | 3937 | 0.654 |
| Prokaryotes | Bacteria | Cyanobacteria | naz | Anabaena azollae 0708 | 3651 | 0.394 |
| Prokaryotes | Bacteria | Actinobacteria | nca | Nocardioides sp. JS614 | 4909 | 0.716 |
| Eukaryotes | Fungi | Ascomycetes | ncr | Neurospora crassa | 9824 | 0.561 |
| Prokaryotes | Bacteria | Actinobacteria | nda | Nocardiopsis dassonvillei | 5497 | 0.728 |
| Prokaryotes | Bacteria | Hyperthermophilic bacteria | nde | Candidatus Nitrospira defluvii | 4268 | 0.593 |
| Prokaryotes | Archaea | Nanoarchaeota | neq | Nanoarchaeum equitans | 540 | 0.318 |
| Prokaryotes | Bacteria | Betaproteobacteria | net | Nitrosomonas eutropha | 2551 | 0.495 |
| Prokaryotes | Bacteria | Betaproteobacteria | neu | Nitrosomonas europaea | 2461 | 0.515 |
| Prokaryotes | Bacteria | Actinobacteria | nfa | Nocardia farcinica | 5934 | 0.710 |
| Eukaryotes | Fungi | Ascomycetes | nfi | Neosartorya fischeri | 10395 | 0.541 |
| Prokaryotes | Bacteria | Betaproteobacteria | ngk | Neisseria gonorrhoeae NCCP11945 | 2674 | 0.535 |
| Prokaryotes | Bacteria | Betaproteobacteria | ngo | Neisseria gonorrhoeae FA 1090 | 2002 | 0.540 |
| Eukaryotes | Protists | Amoeboflagellate | ngr | Naegleria gruberi | 15709 | 0.345 |
| Prokaryotes | Bacteria | Alphaproteobacteria | nha | Nitrobacter hamburgensis | 4326 | 0.624 |
| Prokaryotes | Bacteria | Gammaproteobacteria | nhl | Nitrosococcus halophilus | 3817 | 0.526 |
| Prokaryotes | Bacteria | Epsilonproteobacteria | nis | Nitratiruptor sp. SB155-2 | 1843 | 0.400 |
| Prokaryotes | Bacteria | Betaproteobacteria | nit | Nitrosomonas sp. AL212 | 2983 | 0.460 |
| Prokaryotes | Bacteria | Betaproteobacteria | nla | Neisseria lactamica | 1972 | 0.535 |
| Prokaryotes | Bacteria | Betaproteobacteria | nma | Neisseria meningitidis Z2491 (serogroup A) | 1909 | 0.532 |
| Prokaryotes | Bacteria | Betaproteobacteria | nmc | Neisseria meningitidis FAM18 (serogroup C) | 1917 | 0.530 |
| Prokaryotes | Bacteria | Betaproteobacteria | nme | Neisseria meningitidis MC58 (serogroup B) | 2063 | 0.528 |
| Prokaryotes | Archaea | Euryarchaeota | nmg | Natrialba magadii | 4212 | 0.622 |
| Prokaryotes | Bacteria | Betaproteobacteria | nmi | Neisseria meningitidis alpha14 | 1872 | 0.535 |
| Prokaryotes | Bacteria | Actinobacteria | nml | Nakamurella multipartita | 5240 | 0.711 |
| Prokaryotes | Bacteria | Betaproteobacteria | nmn | Neisseria meningitidis 053442 (serogroup C) | 2020 | 0.531 |
| Prokaryotes | Archaea | Thaumarchaeota | nmr | Nitrosopumilus maritimus | 1796 | 0.347 |
| Prokaryotes | Bacteria | Betaproteobacteria | nmu | Nitrosospira multiformis | 2805 | 0.548 |
| Prokaryotes | Bacteria | Gammaproteobacteria | noc | Nitrosococcus oceani | 3017 | 0.513 |
| Prokaryotes | Archaea | Euryarchaeota | nph | Natronomonas pharaonis | 2820 | 0.637 |
| Prokaryotes | Bacteria | Cyanobacteria | npu | Nostoc punctiforme | 6689 | 0.425 |
| Prokaryotes | Bacteria | Alphaproteobacteria | nri | Neorickettsia risticii | 892 | 0.417 |
| Prokaryotes | Bacteria | Epsilonproteobacteria | nsa | Nitratifractor salsuginis | 2088 | 0.550 |
| Prokaryotes | Bacteria | Alphaproteobacteria | nse | Neorickettsia sennetsu | 932 | 0.415 |
| Prokaryotes | Bacteria | Firmicutes | nth | Natranaerobius thermophilus | 2906 | 0.369 |
| Eukaryotes | Animals | Cnidarians | nve | Nematostella vectensis (sea anemone) | 24773 | 0.473 |
| Eukaryotes | Animals | Arthropods | nvi | Nasonia vitripennis (jewel wasp) | 9658 | 0.473 |
| Prokaryotes | Bacteria | Gammaproteobacteria | nwa | Nitrosococcus watsonii | 2908 | 0.511 |
| Prokaryotes | Bacteria | Alphaproteobacteria | nwi | Nitrobacter winogradskyi | 3122 | 0.630 |
| Eukaryotes | Animals | Vertebrates | oaa | Ornithorhynchus anatinus (platypus) | 16390 | 0.550 |
| Prokaryotes | Bacteria | Alphaproteobacteria | oan | Ochrobactrum anthropi | 4799 | 0.570 |
| Prokaryotes | Bacteria | Alphaproteobacteria | oca | Oligotropha carboxidovorans | 3722 | 0.630 |
| Prokaryotes | Bacteria | Firmicutes | oih | Oceanobacillus iheyensis | 3500 | 0.363 |
| Prokaryotes | Bacteria | Actinobacteria | ols | Olsenella uli | 1739 | 0.652 |
| Eukaryotes | Plants | Green algae | olu | Ostreococcus lucimarinus | 7603 | 0.590 |
| Prokaryotes | Bacteria | Firmicutes | ooe | Oenococcus oeni | 1691 | 0.389 |
| Prokaryotes | Bacteria | Deinococcus | opr | Oceanithermus profundus | 2373 | 0.703 |
| Eukaryotes | Plants | Monocots | osa | Oryza sativa japonica (Japanese rice) | 28453 | 0.548 |
| Prokaryotes | Bacteria | Bacteroidetes | osp | Odoribacter splanchnicus | 3497 | 0.444 |
| Eukaryotes | Plants | Green algae | ota | Ostreococcus tauri | 7990 | 0.586 |
| Prokaryotes | Bacteria | Verrucomicrobia | ote | Opitutus terrae | 4612 | 0.654 |
| Prokaryotes | Bacteria | Alphaproteobacteria | ots | Orientia tsutsugamushi Boryong | 1182 | 0.314 |
| Prokaryotes | Bacteria | Alphaproteobacteria | ott | Orientia tsutsugamushi Ikeda | 1967 | 0.316 |
| Prokaryotes | Bacteria | Green sulfur bacteria | paa | Prosthecochloris aestuarii | 2327 | 0.509 |
| Prokaryotes | Archaea | Euryarchaeota | pab | Pyrococcus abyssi | 1781 | 0.453 |
| Prokaryotes | Bacteria | Actinobacteria | pac | Propionibacterium acnes KPA171202 | 2297 | 0.603 |
| Prokaryotes | Bacteria | Gammaproteobacteria | pae | Pseudomonas aeruginosa PAO1 | 5571 | 0.671 |
| Prokaryotes | Bacteria | Gammaproteobacteria | pag | Pseudomonas aeruginosa LESB58 | 5925 | 0.669 |
| Prokaryotes | Bacteria | Bacteroidetes | pah | Porphyromonas asaccharolytica | 1699 | 0.534 |
| Prokaryotes | Archaea | Crenarchaeota | pai | Pyrobaculum aerophilum | 2604 | 0.520 |
| Prokaryotes | Bacteria | Actinobacteria | pak | Propionibacterium acnes SK137 | 2352 | 0.604 |
| Prokaryotes | Bacteria | Tenericutes | pal | Candidatus Phytoplasma australiense | 684 | 0.292 |
| Prokaryotes | Bacteria | Gammaproteobacteria | pam | Pantoea ananatis | 4241 | 0.546 |
| Eukaryotes | Fungi | Ascomycetes | pan | Podospora anserina | 10271 | 0.558 |
| Prokaryotes | Bacteria | Gammaproteobacteria | pao | Pantoea sp. At-9b | 5770 | 0.554 |
| Prokaryotes | Bacteria | Gammaproteobacteria | pap | Pseudomonas aeruginosa PA7 | 6286 | 0.671 |
| Prokaryotes | Bacteria | Gammaproteobacteria | par | Psychrobacter arcticum | 2120 | 0.447 |
| Prokaryotes | Archaea | Crenarchaeota | pas | Pyrobaculum arsenaticum | 2299 | 0.558 |
| Prokaryotes | Bacteria | Gammaproteobacteria | pat | Pseudoalteromonas atlantica | 4281 | 0.455 |
| Prokaryotes | Bacteria | Gammaproteobacteria | pau | Pseudomonas aeruginosa UCBPP-PA14 | 5892 | 0.669 |
| Prokaryotes | Bacteria | Gammaproteobacteria | pay | Photorhabdus asymbiotica | 4417 | 0.436 |
| Prokaryotes | Bacteria | Gammaproteobacteria | pba | Pseudomonas brassicacearum | 6095 | 0.616 |
| Eukaryotes | Protists | Alveolates | pbe | Plasmodium berghei | 9821 | 0.247 |
| Prokaryotes | Bacteria | Alphaproteobacteria | pbr | Parvularcula bermudensis | 2687 | 0.609 |
| Prokaryotes | Bacteria | Planctomycetes | pbs | Planctomyces brasiliensis | 4750 | 0.568 |
| Prokaryotes | Bacteria | Deltaproteobacteria | pca | Pelobacter carbinolicus | 3352 | 0.560 |
| Eukaryotes | Protists | Alveolates | pcb | Plasmodium chabaudi | 12252 | 0.256 |
| Prokaryotes | Archaea | Crenarchaeota | pcl | Pyrobaculum calidifontis | 2152 | 0.578 |
| Prokaryotes | Bacteria | Gammaproteobacteria | pcr | Psychrobacter cryohalolentis | 2511 | 0.441 |
| Eukaryotes | Fungi | Ascomycetes | pcs | Penicillium chrysogenum | 12791 | 0.529 |
| Prokaryotes | Bacteria | Gammaproteobacteria | pct | Pectobacterium carotovorum | 4246 | 0.532 |
| Prokaryotes | Bacteria | Chlamydiae | pcu | Candidatus Protochlamydia amoebophila | 2031 | 0.362 |
| Prokaryotes | Bacteria | Alphaproteobacteria | pde | Paracoccus denitrificans | 5077 | 0.671 |
| Prokaryotes | Bacteria | Bacteroidetes | pdi | Parabacteroides distasonis | 3850 | 0.461 |
| Prokaryotes | Bacteria | Bacteroidetes | pdn | Prevotella denticola | 2386 | 0.518 |
| Prokaryotes | Bacteria | Actinobacteria | pdx | Pseudonocardia dioxanivorans | 6681 | 0.733 |
| Prokaryotes | Bacteria | Alphaproteobacteria | pel | Candidatus Pelagibacter sp. IMCC9063 | 1447 | 0.320 |
| Prokaryotes | Bacteria | Gammaproteobacteria | pen | Pseudomonas entomophila | 5134 | 0.648 |
| Eukaryotes | Protists | Alveolates | pfa | Plasmodium falciparum 3D7 | 5331 | 0.238 |
| Eukaryotes | Protists | Alveolates | pfd | Plasmodium falciparum Dd2 | 4955 | 0.231 |
| Eukaryotes | Protists | Alveolates | pfh | Plasmodium falciparum HB3 | 5367 | 0.226 |
| Prokaryotes | Bacteria | Gammaproteobacteria | pfl | Pseudomonas fluorescens Pf-5 | 6138 | 0.641 |
| Prokaryotes | Bacteria | Gammaproteobacteria | pfo | Pseudomonas fluorescens Pf0-1 | 5722 | 0.612 |
| Prokaryotes | Bacteria | Actinobacteria | pfr | Propionibacterium freudenreichii | 2375 | 0.674 |
| Prokaryotes | Bacteria | Gammaproteobacteria | pfs | Pseudomonas fluorescens SBW25 | 6395 | 0.608 |
| Prokaryotes | Archaea | Euryarchaeota | pfu | Pyrococcus furiosus | 2125 | 0.413 |
| Prokaryotes | Bacteria | Bacteroidetes | pgi | Porphyromonas gingivalis W83 | 1909 | 0.494 |
| Prokaryotes | Bacteria | Bacteroidetes | pgn | Porphyromonas gingivalis ATCC 33277 | 2090 | 0.494 |
| Eukaryotes | Fungi | Ascomycetes | pgu | Meyerozyma guilliermondii | 5920 | 0.445 |
| Prokaryotes | Bacteria | Alphaproteobacteria | pgv | Polymorphum gilvum | 4393 | 0.676 |
| Prokaryotes | Bacteria | Gammaproteobacteria | pha | Pseudoalteromonas haloplanktis | 3485 | 0.411 |
| Prokaryotes | Bacteria | Bacteroidetes | phe | Pedobacter heparinus | 4252 | 0.428 |
| Prokaryotes | Archaea | Euryarchaeota | pho | Pyrococcus horikoshii | 1955 | 0.423 |
| Eukaryotes | Animals | Arthropods | phu | Pediculus humanus corporis (human body louse) | 10773 | 0.366 |
| Eukaryotes | Fungi | Ascomycetes | pic | Scheffersomyces stipitis | 5816 | 0.427 |
| Eukaryotes | Protists | Oomycetes | pif | Phytophthora infestans | 17797 | 0.539 |
| Prokaryotes | Bacteria | Gammaproteobacteria | pin | Psychromonas ingrahamii | 3545 | 0.414 |
| Prokaryotes | Archaea | Crenarchaeota | pis | Pyrobaculum islandicum | 1978 | 0.497 |
| Prokaryotes | Bacteria | Firmicutes | pjd | Paenibacillus sp. JDR-2 | 6213 | 0.512 |
| Eukaryotes | Protists | Alveolates | pkn | Plasmodium knowlesi | 5102 | 0.402 |
| Prokaryotes | Bacteria | Alphaproteobacteria | pla | Parvibaculum lavamentivorans | 3636 | 0.627 |
| Prokaryotes | Bacteria | Planctomycetes | plm | Planctomyces limnophilus | 4258 | 0.544 |
| Prokaryotes | Bacteria | Green sulfur bacteria | plt | Pelodictyon luteolum | 2083 | 0.581 |
| Prokaryotes | Bacteria | Gammaproteobacteria | plu | Photorhabdus luminescens | 4683 | 0.443 |
| Prokaryotes | Bacteria | Cyanobacteria | pma | Prochlorococcus marinus SS120 | 1883 | 0.371 |
| Prokaryotes | Bacteria | Cyanobacteria | pmb | Prochlorococcus marinus AS9601 | 1920 | 0.321 |
| Prokaryotes | Bacteria | Cyanobacteria | pmc | Prochlorococcus marinus MIT 9515 | 1905 | 0.318 |
| Prokaryotes | Bacteria | Cyanobacteria | pme | Prochlorococcus marinus NATL1A | 2193 | 0.359 |
| Prokaryotes | Bacteria | Cyanobacteria | pmf | Prochlorococcus marinus MIT 9303 | 2997 | 0.512 |
| Prokaryotes | Bacteria | Cyanobacteria | pmg | Prochlorococcus marinus MIT 9301 | 1906 | 0.322 |
| Prokaryotes | Bacteria | Cyanobacteria | pmh | Prochlorococcus marinus MIT 9215 | 1982 | 0.320 |
| Prokaryotes | Bacteria | Cyanobacteria | pmi | Prochlorococcus marinus MIT9312 | 1810 | 0.321 |
| Prokaryotes | Bacteria | Cyanobacteria | pmj | Prochlorococcus marinus MIT 9211 | 1854 | 0.387 |
| Prokaryotes | Bacteria | Gammaproteobacteria | pmk | Pseudomonas mendocina NK-01 | 4958 | 0.632 |
| Prokaryotes | Bacteria | Tenericutes | pml | Candidatus Phytoplasma mali | 479 | 0.232 |
| Prokaryotes | Bacteria | Cyanobacteria | pmm | Prochlorococcus marinus MED4 | 1717 | 0.318 |
| Prokaryotes | Bacteria | Cyanobacteria | pmn | Prochlorococcus marinus NATL2A | 2162 | 0.360 |
| Prokaryotes | Bacteria | Hyperthermophilic bacteria | pmo | Petrotoga mobilis | 1898 | 0.344 |
| Prokaryotes | Bacteria | Gammaproteobacteria | pmr | Proteus mirabilis | 3662 | 0.403 |
| Prokaryotes | Bacteria | Cyanobacteria | pmt | Prochlorococcus marinus MIT 9313 | 2269 | 0.522 |
| Prokaryotes | Bacteria | Gammaproteobacteria | pmu | Pasteurella multocida | 2015 | 0.412 |
| Prokaryotes | Bacteria | Hyperthermophilic bacteria | pmx | Persephonella marina | 2051 | 0.374 |
| Prokaryotes | Bacteria | Gammaproteobacteria | pmy | Pseudomonas mendocina ymp | 4594 | 0.652 |
| Prokaryotes | Bacteria | Bacteroidetes | pmz | Prevotella melaninogenica | 2296 | 0.428 |
| Prokaryotes | Bacteria | Betaproteobacteria | pna | Polaromonas naphthalenivorans | 4929 | 0.628 |
| Prokaryotes | Bacteria | Betaproteobacteria | pne | Polynucleobacter necessarius | 1508 | 0.460 |
| Eukaryotes | Fungi | Ascomycetes | pno | Phaeosphaeria nodorum | 15998 | 0.543 |
| Prokaryotes | Bacteria | Betaproteobacteria | pnu | Polynucleobacter sp. QLW-P1DMWA-1 | 2077 | 0.453 |
| Prokaryotes | Bacteria | Betaproteobacteria | pol | Polaromonas sp. JS666 | 5453 | 0.627 |
| Eukaryotes | Animals | Vertebrates | pon | Pongo abelii (Sumatran orangutan) | 23880 | 0.522 |
| Eukaryotes | Plants | Eudicots | pop | Populus trichocarpa (black cottonwood) | 40484 | 0.438 |
| Prokaryotes | Bacteria | Tenericutes | poy | Phytoplasma OY | 750 | 0.294 |
| Eukaryotes | Fungi | Ascomycetes | ppa | Pichia pastoris | 5040 | 0.416 |
| Prokaryotes | Bacteria | Deltaproteobacteria | ppd | Pelobacter propionicus | 3804 | 0.592 |
| Prokaryotes | Bacteria | Firmicutes | ppe | Pediococcus pentosaceus | 1755 | 0.381 |
| Prokaryotes | Bacteria | Gammaproteobacteria | ppf | Pseudomonas putida F1 | 5250 | 0.625 |
| Prokaryotes | Bacteria | Gammaproteobacteria | ppg | Pseudomonas putida GB-1 | 5408 | 0.626 |
| Prokaryotes | Bacteria | Green sulfur bacteria | pph | Pelodictyon phaeoclathratiforme | 2707 | 0.489 |
| Eukaryotes | Fungi | Basidiomycetes | ppl | Postia placenta | 9083 | 0.567 |
| Prokaryotes | Bacteria | Firmicutes | ppm | Paenibacillus polymyxa SC2 | 6032 | 0.458 |
| Prokaryotes | Bacteria | Bacteroidetes | ppn | Paludibacter propionicigenes | 3020 | 0.400 |
| Eukaryotes | Plants | Mosses | ppp | Physcomitrella patens subsp. patens | 35925 | 0.472 |
| Prokaryotes | Bacteria | Gammaproteobacteria | ppr | Photobacterium profundum | 5489 | 0.428 |
| Prokaryotes | Bacteria | Gammaproteobacteria | ppu | Pseudomonas putida KT2440 | 5350 | 0.623 |
| Prokaryotes | Bacteria | Gammaproteobacteria | ppw | Pseudomonas putida W619 | 5182 | 0.621 |
| Prokaryotes | Bacteria | Firmicutes | ppy | Paenibacillus polymyxa E681 | 4805 | 0.469 |
| Prokaryotes | Bacteria | Bacteroidetes | pru | Prevotella ruminicola | 2763 | 0.489 |
| Prokaryotes | Bacteria | Gammaproteobacteria | prw | Psychrobacter sp. PRwf-1 | 2385 | 0.464 |
| Prokaryotes | Bacteria | Gammaproteobacteria | psa | Pseudomonas stutzeri | 4128 | 0.643 |
| Prokaryotes | Bacteria | Gammaproteobacteria | psb | Pseudomonas syringae pv. syringae B728a | 5089 | 0.600 |
| Prokaryotes | Bacteria | Planctomycetes | psl | Pirellula staleyi | 4717 | 0.579 |
| Prokaryotes | Bacteria | Gammaproteobacteria | psm | Pseudoalteromonas sp. SM9913 | 3712 | 0.411 |
| Prokaryotes | Bacteria | Bacteroidetes | psn | Pedobacter saltans | 3792 | 0.372 |
| Prokaryotes | Bacteria | Gammaproteobacteria | psp | Pseudomonas syringae pv. phaseolicola 1448A | 5172 | 0.587 |
| Prokaryotes | Bacteria | Gammaproteobacteria | pst | Pseudomonas syringae pv. tomato DC3000 | 5619 | 0.592 |
| Prokaryotes | Bacteria | Gammaproteobacteria | psu | Pseudoxanthomonas suwonensis | 3070 | 0.704 |
| Prokaryotes | Bacteria | Firmicutes | pth | Pelotomaculum thermopropionicum | 2919 | 0.546 |
| Eukaryotes | Protists | Diatoms | pti | Phaeodactylum tricornutum | 10392 | 0.511 |
| Eukaryotes | Protists | Alveolates | ptm | Paramecium tetraurelia | 40043 | 0.300 |
| Prokaryotes | Archaea | Euryarchaeota | pto | Picrophilus torridus | 1537 | 0.372 |
| Eukaryotes | Animals | Vertebrates | ptr | Pan troglodytes (chimpanzee) | 25209 | 0.527 |
| Prokaryotes | Bacteria | Alphaproteobacteria | pub | Candidatus Pelagibacter ubique | 1354 | 0.299 |
| Prokaryotes | Bacteria | Betaproteobacteria | put | Pusillimonas sp. T7-7 | 3773 | 0.576 |
| Prokaryotes | Bacteria | Gammaproteobacteria | pva | Pantoea vagans | 4590 | 0.564 |
| Prokaryotes | Bacteria | Green sulfur bacteria | pvi | Chlorobium vibrioformis | 1753 | 0.536 |
| Eukaryotes | Protists | Alveolates | pvx | Plasmodium vivax | 5392 | 0.463 |
| Prokaryotes | Bacteria | Gammaproteobacteria | pwa | Pectobacterium wasabiae | 4437 | 0.518 |
| Prokaryotes | Archaea | Euryarchaeota | pyn | Pyrococcus sp. NA2 | 1980 | 0.432 |
| Eukaryotes | Protists | Alveolates | pyo | Plasmodium yoelii | 7353 | 0.248 |
| Prokaryotes | Bacteria | Alphaproteobacteria | pzu | Phenylobacterium zucineum | 3854 | 0.713 |
| Prokaryotes | Bacteria | Alphaproteobacteria | raf | Rickettsia africae | 1041 | 0.330 |
| Prokaryotes | Bacteria | Gammaproteobacteria | rah | Rahnella sp. Y9602 | 5111 | 0.534 |
| Prokaryotes | Bacteria | Alphaproteobacteria | rak | Rickettsia akari | 1258 | 0.329 |
| Prokaryotes | Bacteria | Firmicutes | ral | Ruminococcus albus | 3872 | 0.447 |
| Prokaryotes | Bacteria | Bacteroidetes | ran | Riemerella anatipestifer | 1972 | 0.358 |
| Prokaryotes | Bacteria | Planctomycetes | rba | Rhodopirellula baltica | 7325 | 0.555 |
| Prokaryotes | Bacteria | Alphaproteobacteria | rbe | Rickettsia bellii RML369-C | 1429 | 0.320 |
| Prokaryotes | Bacteria | Bacteroidetes | rbi | Robiginitalea biformata | 3209 | 0.561 |
| Prokaryotes | Bacteria | Alphaproteobacteria | rbo | Rickettsia bellii OSU 85-389 | 1475 | 0.319 |
| Prokaryotes | Bacteria | Green nonsulfur bacteria | rca | Roseiflexus castenholzii DSM13941 | 4330 | 0.609 |
| Prokaryotes | Bacteria | Alphaproteobacteria | rce | Rhodospirillum centenum | 4003 | 0.706 |
| Prokaryotes | Archaea | Euryarchaeota | rci | Uncultured methanogenic archaeon RC-I | 3089 | 0.563 |
| Prokaryotes | Bacteria | Alphaproteobacteria | rcm | Rickettsia canadensis | 1090 | 0.320 |
| Prokaryotes | Bacteria | Alphaproteobacteria | rco | Rickettsia conorii | 1374 | 0.330 |
| Prokaryotes | Bacteria | Alphaproteobacteria | rcp | Rhodobacter capsulatus | 3642 | 0.668 |
| Eukaryotes | Plants | Eudicots | rcu | Ricinus communis (castor bean) | 31258 | 0.448 |
| Prokaryotes | Bacteria | Alphaproteobacteria | rde | Roseobacter denitrificans | 4129 | 0.594 |
| Prokaryotes | Bacteria | Actinobacteria | rdn | Rothia dentocariosa | 2217 | 0.548 |
| Prokaryotes | Bacteria | Alphaproteobacteria | rec | Rhizobium etli CIAT 652 | 6056 | 0.620 |
| Prokaryotes | Bacteria | Betaproteobacteria | reh | Ralstonia eutropha H16 | 6626 | 0.669 |
| Prokaryotes | Bacteria | Actinobacteria | req | Rhodococcus equi | 4512 | 0.692 |
| Prokaryotes | Bacteria | Actinobacteria | rer | Rhodococcus erythropolis | 6433 | 0.628 |
| Prokaryotes | Bacteria | Alphaproteobacteria | ret | Rhizobium etli CFN 42 | 5963 | 0.617 |
| Prokaryotes | Bacteria | Betaproteobacteria | reu | Ralstonia eutropha JMP134 | 6446 | 0.650 |
| Prokaryotes | Bacteria | Alphaproteobacteria | rfe | Rickettsia felis | 1512 | 0.328 |
| Prokaryotes | Bacteria | Betaproteobacteria | rfr | Rhodoferax ferrireducens | 4418 | 0.603 |
| Prokaryotes | Bacteria | Actinobacteria | rha | Rhodococcus sp. RHA1 | 9145 | 0.674 |
| Prokaryotes | Bacteria | Alphaproteobacteria | rhi | Sinorhizobium fredii NGR234 | 6363 | 0.631 |
| Prokaryotes | Bacteria | Gammaproteobacteria | rip | Candidatus Riesia pediculicola | 556 | 0.300 |
| Prokaryotes | Bacteria | Alphaproteobacteria | rle | Rhizobium leguminosarum | 7143 | 0.616 |
| Prokaryotes | Bacteria | Alphaproteobacteria | rlg | Rhizobium leguminosarum bv. trifolii WSM1325 | 7001 | 0.616 |
| Prokaryotes | Bacteria | Alphaproteobacteria | rlt | Rhizobium leguminosarum bv. trifolii WSM2304 | 6415 | 0.620 |
| Prokaryotes | Bacteria | Gammaproteobacteria | rma | Candidatus Ruthia magnifica | 976 | 0.353 |
| Prokaryotes | Bacteria | Betaproteobacteria | rme | Cupriavidus metallidurans | 6355 | 0.641 |
| Prokaryotes | Bacteria | Bacteroidetes | rmr | Rhodothermus marinus | 2863 | 0.650 |
| Prokaryotes | Bacteria | Alphaproteobacteria | rms | Rickettsia massiliae | 980 | 0.330 |
| Prokaryotes | Bacteria | Actinobacteria | rmu | Rothia mucilaginosa | 1904 | 0.609 |
| Eukaryotes | Animals | Vertebrates | rno | Rattus norvegicus (rat) | 23979 | 0.518 |
| Prokaryotes | Bacteria | Actinobacteria | rop | Rhodococcus opacus | 8197 | 0.681 |
| Prokaryotes | Bacteria | Alphaproteobacteria | rpa | Rhodopseudomonas palustris CGA009 | 4820 | 0.655 |
| Prokaryotes | Bacteria | Alphaproteobacteria | rpb | Rhodopseudomonas palustris HaA2 | 4683 | 0.664 |
| Prokaryotes | Bacteria | Alphaproteobacteria | rpc | Rhodopseudomonas palustris BisB18 | 4886 | 0.654 |
| Prokaryotes | Bacteria | Alphaproteobacteria | rpd | Rhodopseudomonas palustris BisB5 | 4397 | 0.654 |
| Prokaryotes | Bacteria | Alphaproteobacteria | rpe | Rhodopseudomonas palustris BisA53 | 4878 | 0.650 |
| Prokaryotes | Bacteria | Betaproteobacteria | rpf | Ralstonia pickettii 12D | 5361 | 0.639 |
| Prokaryotes | Bacteria | Betaproteobacteria | rpi | Ralstonia pickettii 12J | 4952 | 0.642 |
| Prokaryotes | Bacteria | Alphaproteobacteria | rpk | Rickettsia peacockii | 947 | 0.334 |
| Prokaryotes | Bacteria | Alphaproteobacteria | rpr | Rickettsia prowazekii | 835 | 0.306 |
| Prokaryotes | Bacteria | Alphaproteobacteria | rpt | Rhodopseudomonas palustris TIE-1 | 5246 | 0.654 |
| Prokaryotes | Bacteria | Alphaproteobacteria | rpx | Rhodopseudomonas palustris DX-1 | 4917 | 0.659 |
| Prokaryotes | Bacteria | Alphaproteobacteria | rri | Rickettsia rickettsii Sheila Smith | 1343 | 0.330 |
| Prokaryotes | Bacteria | Alphaproteobacteria | rrj | Rickettsia rickettsii Iowa | 1384 | 0.329 |
| Prokaryotes | Bacteria | Green nonsulfur bacteria | rrs | Roseiflexus sp. RS-1 | 4517 | 0.609 |
| Prokaryotes | Bacteria | Alphaproteobacteria | rru | Rhodospirillum rubrum | 3841 | 0.660 |
| Prokaryotes | Bacteria | Actinobacteria | rsa | Renibacterium salmoninarum | 3507 | 0.566 |
| Prokaryotes | Bacteria | Betaproteobacteria | rsc | Ralstonia solanacearum CFBP2957 | 3223 | 0.670 |
| Prokaryotes | Bacteria | Elusimicrobia | rsd | Uncultured Termite group 1 bacterium phylotype Rs-D17 | 776 | 0.364 |
| Prokaryotes | Bacteria | Alphaproteobacteria | rsh | Rhodobacter sphaeroides ATCC 17029 | 4131 | 0.691 |
| Prokaryotes | Bacteria | Alphaproteobacteria | rsk | Rhodobacter sphaeroides KD131 | 4569 | 0.693 |
| Prokaryotes | Bacteria | Betaproteobacteria | rsl | Ralstonia solanacearum PSI07 | 4978 | 0.669 |
| Prokaryotes | Bacteria | Betaproteobacteria | rso | Ralstonia solanacearum GMI1000 | 5113 | 0.675 |
| Prokaryotes | Bacteria | Alphaproteobacteria | rsp | Rhodobacter sphaeroides 2.4.1 | 4242 | 0.689 |
| Prokaryotes | Bacteria | Alphaproteobacteria | rsq | Rhodobacter sphaeroides ATCC 17025 | 4333 | 0.685 |
| Prokaryotes | Bacteria | Alphaproteobacteria | rty | Rickettsia typhi | 838 | 0.304 |
| Prokaryotes | Bacteria | Alphaproteobacteria | rva | Rhodomicrobium vannielii | 3565 | 0.630 |
| Prokaryotes | Bacteria | Actinobacteria | rxy | Rubrobacter xylanophilus | 3140 | 0.709 |
| Prokaryotes | Bacteria | Firmicutes | saa | Staphylococcus aureus USA300_FPR3757 (CA-MRSA) | 2604 | 0.336 |
| Prokaryotes | Bacteria | Firmicutes | sab | Staphylococcus aureus RF122 | 2509 | 0.337 |
| Prokaryotes | Bacteria | Firmicutes | sac | Staphylococcus aureus COL (MRSA) | 2615 | 0.337 |
| Prokaryotes | Bacteria | Firmicutes | sad | Staphylococcus aureus ED98 | 2689 | 0.337 |
| Prokaryotes | Bacteria | Firmicutes | sae | Staphylococcus aureus Newman | 2614 | 0.338 |
| Prokaryotes | Bacteria | Hyperthermophilic bacteria | saf | Sulfurihydrogenibium azorense | 1723 | 0.329 |
| Prokaryotes | Bacteria | Firmicutes | sag | Streptococcus agalactiae 2603 (serotype V) | 2124 | 0.364 |
| Prokaryotes | Bacteria | Firmicutes | sah | Staphylococcus aureus JH1 (MRSA/VSSA) | 2780 | 0.338 |
| Prokaryotes | Archaea | Crenarchaeota | sai | Sulfolobus acidocaldarius | 2224 | 0.376 |
| Prokaryotes | Bacteria | Firmicutes | saj | Staphylococcus aureus JH9 (MRSA/VRSA) | 2726 | 0.338 |
| Prokaryotes | Bacteria | Firmicutes | sak | Streptococcus agalactiae A909 (serotype Ia) | 1996 | 0.364 |
| Prokaryotes | Bacteria | Alphaproteobacteria | sal | Sphingopyxis alaskensis | 3195 | 0.658 |
| Prokaryotes | Bacteria | Firmicutes | sam | Staphylococcus aureus MW2 (CA-MRSA) | 2624 | 0.337 |
| Prokaryotes | Bacteria | Firmicutes | san | Streptococcus agalactiae NEM316 (serotype III) | 2094 | 0.364 |
| Prokaryotes | Bacteria | Firmicutes | sao | Staphylococcus aureus NCTC8325 | 2891 | 0.337 |
| Prokaryotes | Bacteria | Actinobacteria | saq | Salinispora arenicola | 4917 | 0.698 |
| Prokaryotes | Bacteria | Firmicutes | sar | Staphylococcus aureus MRSA252 (MRSA) | 2650 | 0.336 |
| Prokaryotes | Bacteria | Firmicutes | sas | Staphylococcus aureus MSSA476 (MSSA) | 2590 | 0.337 |
| Prokaryotes | Bacteria | Deltaproteobacteria | sat | Syntrophus aciditrophicus | 3166 | 0.525 |
| Prokaryotes | Bacteria | Firmicutes | sau | Staphylococcus aureus N315 (MRSA/VSSA) | 2614 | 0.337 |
| Prokaryotes | Bacteria | Firmicutes | sav | Staphylococcus aureus Mu50 (MRSA/VISA) | 2730 | 0.337 |
| Prokaryotes | Bacteria | Firmicutes | saw | Staphylococcus aureus Mu3 (MRSA/hetero-VISA) | 2690 | 0.338 |
| Prokaryotes | Bacteria | Firmicutes | sax | Staphylococcus aureus USA300_TCH1516 (CA-MSSA) | 2689 | 0.336 |
| Prokaryotes | Bacteria | Gammaproteobacteria | saz | Shewanella amazonensis | 3645 | 0.544 |
| Prokaryotes | Bacteria | Gammaproteobacteria | sbc | Shigella boydii CDC 3083-94 | 4557 | 0.521 |
| Eukaryotes | Plants | Monocots | sbi | Sorghum bicolor (sorghum) | 33002 | 0.546 |
| Prokaryotes | Bacteria | Gammaproteobacteria | sbl | Shewanella baltica OS155 | 4489 | 0.473 |
| Prokaryotes | Bacteria | Gammaproteobacteria | sbm | Shewanella baltica OS185 | 4394 | 0.474 |
| Prokaryotes | Bacteria | Gammaproteobacteria | sbn | Shewanella baltica OS195 | 4688 | 0.473 |
| Prokaryotes | Bacteria | Gammaproteobacteria | sbo | Shigella boydii Sb227 | 4281 | 0.522 |
| Prokaryotes | Bacteria | Gammaproteobacteria | sbp | Shewanella baltica OS223 | 4436 | 0.474 |
| Prokaryotes | Bacteria | Spirochaetes | sbu | Spirochaeta sp. Buddy | 3017 | 0.495 |
| Prokaryotes | Bacteria | Firmicutes | sca | Staphylococcus carnosus | 2461 | 0.355 |
| Prokaryotes | Bacteria | Actinobacteria | scb | Streptomyces scabiei | 8746 | 0.717 |
| Prokaryotes | Bacteria | Spirochaetes | scc | Spirochaeta coccoides | 1822 | 0.513 |
| Eukaryotes | Fungi | Ascomycetes | sce | Saccharomyces cerevisiae (budding yeast) | 5882 | 0.396 |
| Prokaryotes | Bacteria | Deltaproteobacteria | scl | Sorangium cellulosum | 9381 | 0.716 |
| Eukaryotes | Fungi | Basidiomycetes | scm | Schizophyllum commune | 13190 | 0.600 |
| Prokaryotes | Bacteria | Actinobacteria | sco | Streptomyces coelicolor | 8153 | 0.723 |
| Prokaryotes | Bacteria | Gammaproteobacteria | sde | Saccharophagus degradans | 4007 | 0.466 |
| Prokaryotes | Bacteria | Epsilonproteobacteria | sdl | Sulfurospirillum deleyianum | 2265 | 0.394 |
| Prokaryotes | Bacteria | Gammaproteobacteria | sdn | Shewanella denitrificans | 3754 | 0.462 |
| Prokaryotes | Bacteria | Firmicutes | sds | Streptococcus dysgalactiae | 2094 | 0.403 |
| Prokaryotes | Bacteria | Gammaproteobacteria | sdy | Shigella dysenteriae | 4501 | 0.521 |
| Prokaryotes | Bacteria | Gammaproteobacteria | sea | Salmonella enterica subsp. enterica serovar Agona | 4615 | 0.531 |
| Prokaryotes | Bacteria | Gammaproteobacteria | sec | Salmonella enterica subsp. enterica serovar Choleraesuis | 4627 | 0.532 |
| Prokaryotes | Bacteria | Gammaproteobacteria | sed | Salmonella enterica subsp. enterica serovar Dublin | 4617 | 0.532 |
| Prokaryotes | Bacteria | Gammaproteobacteria | see | Salmonella enterica subsp. enterica serovar Newport | 4806 | 0.533 |
| Prokaryotes | Bacteria | Gammaproteobacteria | seg | Salmonella enterica subsp. enterica serovar Gallinarum | 3965 | 0.534 |
| Prokaryotes | Bacteria | Gammaproteobacteria | seh | Salmonella enterica subsp. enterica serovar Heidelberg | 4780 | 0.531 |
| Prokaryotes | Bacteria | Gammaproteobacteria | sei | Salmonella enterica subsp. enterica serovar Paratyphi C | 4638 | 0.532 |
| Prokaryotes | Bacteria | Gammaproteobacteria | sek | Salmonella enterica subsp. enterica serovar Paratyphi A AKU12601 | 4075 | 0.533 |
| Prokaryotes | Bacteria | Actinobacteria | sen | Saccharopolyspora erythraea | 7197 | 0.716 |
| Prokaryotes | Bacteria | Firmicutes | sep | Staphylococcus epidermidis ATCC 12228 | 2482 | 0.331 |
| Prokaryotes | Bacteria | Firmicutes | seq | Streptococcus equi subsp. zooepidemicus H70 | 1869 | 0.424 |
| Prokaryotes | Bacteria | Firmicutes | ser | Staphylococcus epidermidis RP62A | 2525 | 0.332 |
| Prokaryotes | Bacteria | Gammaproteobacteria | ses | Salmonella enterica subsp. arizonae | 4498 | 0.525 |
| Prokaryotes | Bacteria | Gammaproteobacteria | set | Salmonella enterica subsp. enterica serovar Enteritidis | 4206 | 0.533 |
| Prokaryotes | Bacteria | Firmicutes | seu | Streptococcus equi subsp. equi 4047 | 2001 | 0.421 |
| Prokaryotes | Bacteria | Gammaproteobacteria | sew | Salmonella enterica subsp. enterica serovar Schwarzengrund | 4630 | 0.533 |
| Prokaryotes | Bacteria | Firmicutes | sez | Streptococcus equi subsp. zooepidemicus MGCS10565 | 1893 | 0.427 |
| Prokaryotes | Bacteria | Gammaproteobacteria | sfl | Shigella flexneri 301 (serotype 2a) | 4439 | 0.516 |
| Prokaryotes | Bacteria | Gammaproteobacteria | sfr | Shewanella frigidimarina | 4029 | 0.426 |
| Prokaryotes | Bacteria | Deltaproteobacteria | sfu | Syntrophobacter fumaroxidans | 4064 | 0.602 |
| Prokaryotes | Bacteria | Gammaproteobacteria | sfv | Shigella flexneri 8401 (serotype 5b) | 4114 | 0.520 |
| Prokaryotes | Bacteria | Gammaproteobacteria | sfx | Shigella flexneri 2457T (serotype 2a) | 4060 | 0.521 |
| Prokaryotes | Bacteria | Firmicutes | sga | Streptococcus gallolyticus UCN34 | 2223 | 0.385 |
| Prokaryotes | Bacteria | Firmicutes | sgg | Streptococcus gallolyticus subsp. gallolyticus | 2329 | 0.384 |
| Prokaryotes | Bacteria | Gammaproteobacteria | sgl | Sodalis glossinidius | 2516 | 0.561 |
| Prokaryotes | Bacteria | Firmicutes | sgo | Streptococcus gordonii | 2051 | 0.415 |
| Prokaryotes | Bacteria | Actinobacteria | sgr | Streptomyces griseus | 7136 | 0.723 |
| Prokaryotes | Bacteria | Firmicutes | sgy | Syntrophobotulus glycolicus | 3251 | 0.473 |
| Prokaryotes | Bacteria | Firmicutes | sha | Staphylococcus haemolyticus | 2692 | 0.336 |
| Prokaryotes | Archaea | Crenarchaeota | shc | Staphylothermus hellenicus | 1599 | 0.374 |
| Prokaryotes | Bacteria | Gammaproteobacteria | she | Shewanella sp. MR-4 | 3924 | 0.490 |
| Prokaryotes | Bacteria | Bacteroidetes | shg | Sphingobacterium sp. 21 | 5169 | 0.419 |
| Prokaryotes | Bacteria | Actinobacteria | shi | Slackia heliotrinireducens | 2765 | 0.610 |
| Prokaryotes | Bacteria | Gammaproteobacteria | shl | Shewanella halifaxensis | 4278 | 0.458 |
| Prokaryotes | Bacteria | Gammaproteobacteria | shm | Shewanella sp. MR-7 | 4014 | 0.490 |
| Prokaryotes | Bacteria | Gammaproteobacteria | shn | Shewanella sp. ANA-3 | 4360 | 0.490 |
| Prokaryotes | Bacteria | Gammaproteobacteria | shw | Shewanella sp. W3-18-1 | 4044 | 0.456 |
| Prokaryotes | Archaea | Crenarchaeota | sia | Sulfolobus islandicus M.14.25 | 2608 | 0.359 |
| Prokaryotes | Archaea | Crenarchaeota | sid | Sulfolobus islandicus M.16.4 | 2735 | 0.357 |
| Prokaryotes | Archaea | Crenarchaeota | sii | Sulfolobus islandicus L.D.8.5 | 2948 | 0.359 |
| Prokaryotes | Bacteria | Alphaproteobacteria | sil | Silicibacter pomeroyi | 4252 | 0.646 |
| Prokaryotes | Archaea | Crenarchaeota | sim | Sulfolobus islandicus M.16.27 | 2657 | 0.358 |
| Prokaryotes | Archaea | Crenarchaeota | sin | Sulfolobus islandicus Y.N.15.51 | 2900 | 0.361 |
| Prokaryotes | Archaea | Crenarchaeota | sis | Sulfolobus islandicus L.S.2.15 | 2737 | 0.358 |
| Prokaryotes | Bacteria | Alphaproteobacteria | sit | Ruegeria sp. TM1040 | 3864 | 0.605 |
| Prokaryotes | Archaea | Crenarchaeota | siy | Sulfolobus islandicus Y.G.57.14 | 2903 | 0.361 |
| Prokaryotes | Bacteria | Firmicutes | sjj | Streptococcus pneumoniae JJA | 2123 | 0.407 |
| Prokaryotes | Bacteria | Alphaproteobacteria | sjp | Sphingobium japonicum | 4394 | 0.654 |
| Prokaryotes | Bacteria | Actinobacteria | ske | Sanguibacter keddieii | 3710 | 0.720 |
| Prokaryotes | Bacteria | Epsilonproteobacteria | sku | Sulfuricurvum kujiense | 2798 | 0.452 |
| Prokaryotes | Bacteria | Firmicutes | slg | Staphylococcus lugdunensis | 2490 | 0.346 |
| Prokaryotes | Bacteria | Bacteroidetes | sli | Spirosoma linguale | 6938 | 0.513 |
| Prokaryotes | Bacteria | Gammaproteobacteria | slo | Shewanella loihica | 3859 | 0.553 |
| Prokaryotes | Bacteria | Firmicutes | slp | Syntrophothermus lipocalidus | 2313 | 0.516 |
| Prokaryotes | Bacteria | Betaproteobacteria | slt | Sideroxydans lithotrophicus | 2980 | 0.582 |
| Prokaryotes | Bacteria | Actinobacteria | sma | Streptomyces avermitilis | 7676 | 0.711 |
| Prokaryotes | Bacteria | Firmicutes | smb | Streptococcus mitis B6 | 2004 | 0.409 |
| Prokaryotes | Bacteria | Firmicutes | smc | Streptococcus mutans NN2025 | 1895 | 0.378 |
| Prokaryotes | Bacteria | Alphaproteobacteria | smd | Sinorhizobium medicae | 6213 | 0.617 |
| Prokaryotes | Bacteria | Alphaproteobacteria | sme | Sinorhizobium meliloti | 6218 | 0.628 |
| Prokaryotes | Bacteria | Fusobacteria | smf | Streptobacillus moniliformis | 1442 | 0.266 |
| Prokaryotes | Bacteria | Bacteroidetes | smg | Candidatus Sulcia muelleri GWSS | 227 | 0.228 |
| Prokaryotes | Bacteria | Bacteroidetes | smh | Candidatus Sulcia muelleri DMIN | 226 | 0.228 |
| Prokaryotes | Bacteria | Gammaproteobacteria | sml | Stenotrophomonas maltophilia K279a | 4386 | 0.666 |
| Eukaryotes | Animals | Flatworms | smm | Schistosoma mansoni | 12857 | 0.362 |
| Prokaryotes | Archaea | Crenarchaeota | smr | Staphylothermus marinus | 1573 | 0.363 |
| Prokaryotes | Bacteria | Bacteroidetes | sms | Candidatus Sulcia muelleri SMDSEM | 242 | 0.230 |
| Prokaryotes | Bacteria | Gammaproteobacteria | smt | Stenotrophomonas maltophilia R551-3 | 4039 | 0.666 |
| Prokaryotes | Bacteria | Firmicutes | smu | Streptococcus mutans UA159 | 1960 | 0.377 |
| Prokaryotes | Bacteria | Actinobacteria | sna | Stackebrandtia nassauensis | 6379 | 0.686 |
| Prokaryotes | Bacteria | Firmicutes | snb | Streptococcus pneumoniae 670-6B | 2352 | 0.406 |
| Prokaryotes | Bacteria | Firmicutes | snc | Streptococcus pneumoniae TCH8431/19A | 2275 | 0.407 |
| Prokaryotes | Bacteria | Firmicutes | sne | Streptococcus pneumoniae ATCC 700669 (serotype 23F ST81 lineage) | 1990 | 0.404 |
| Prokaryotes | Bacteria | Firmicutes | snm | Streptococcus pneumoniae 70585 | 2202 | 0.407 |
| Prokaryotes | Bacteria | Alphaproteobacteria | sno | Starkeya novella | 4431 | 0.684 |
| Prokaryotes | Bacteria | Firmicutes | snp | Streptococcus pneumoniae AP200 | 2216 | 0.405 |
| Prokaryotes | Bacteria | Firmicutes | snt | Streptococcus pneumoniae Taiwan19F-14 | 2044 | 0.408 |
| Prokaryotes | Bacteria | Gammaproteobacteria | son | Shewanella oneidensis | 4467 | 0.469 |
| Prokaryotes | Bacteria | Firmicutes | sor | Streptococcus oralis | 1907 | 0.420 |
| Prokaryotes | Bacteria | Firmicutes | soz | Streptococcus pyogenes NZ131 (serotype M49) | 1700 | 0.394 |
| Prokaryotes | Bacteria | Firmicutes | spa | Streptococcus pyogenes MGAS10394 (serotype M6) | 1886 | 0.395 |
| Prokaryotes | Bacteria | Firmicutes | spb | Streptococcus pyogenes MGAS6180 (serotype M28) | 1894 | 0.392 |
| Prokaryotes | Bacteria | Gammaproteobacteria | spc | Shewanella putrefaciens | 3972 | 0.454 |
| Prokaryotes | Bacteria | Firmicutes | spd | Streptococcus pneumoniae D39 (virulent serotype 2) | 1914 | 0.407 |
| Prokaryotes | Bacteria | Gammaproteobacteria | spe | Serratia proteamaculans | 4942 | 0.563 |
| Prokaryotes | Bacteria | Firmicutes | spf | Streptococcus pyogenes Manfredo (serotype M5) | 1745 | 0.394 |
| Prokaryotes | Bacteria | Firmicutes | spg | Streptococcus pyogenes MGAS315 (serotype M3) | 1865 | 0.394 |
| Prokaryotes | Bacteria | Firmicutes | sph | Streptococcus pyogenes MGAS10270 (serotype M2) | 1986 | 0.392 |
| Prokaryotes | Bacteria | Firmicutes | spi | Streptococcus pyogenes MGAS10750 (serotype M4) | 1979 | 0.391 |
| Prokaryotes | Bacteria | Firmicutes | spj | Streptococcus pyogenes MGAS2096 (serotype M12) | 1898 | 0.395 |
| Prokaryotes | Bacteria | Firmicutes | spk | Streptococcus pyogenes MGAS9429 (serotype M12) | 1877 | 0.393 |
| Prokaryotes | Bacteria | Gammaproteobacteria | spl | Shewanella pealeana | 4241 | 0.458 |
| Prokaryotes | Bacteria | Firmicutes | spm | Streptococcus pyogenes MGAS8232 (serotype M18) | 1839 | 0.394 |
| Prokaryotes | Bacteria | Firmicutes | spn | Streptococcus pneumoniae TIGR4 (virulent serotype 4) | 2105 | 0.406 |
| Eukaryotes | Fungi | Ascomycetes | spo | Schizosaccharomyces pombe (fission yeast) | 5020 | 0.394 |
| Prokaryotes | Bacteria | Firmicutes | spp | Streptococcus pneumoniae P1031 | 2073 | 0.407 |
| Prokaryotes | Bacteria | Gammaproteobacteria | spq | Salmonella enterica subsp. enterica serovar Paratyphi B | 5592 | 0.529 |
| Prokaryotes | Bacteria | Firmicutes | spr | Streptococcus pneumoniae R6 (avirulent) | 2042 | 0.407 |
| Prokaryotes | Bacteria | Firmicutes | sps | Streptococcus pyogenes SSI-1 (serotype M3) | 1859 | 0.394 |
| Prokaryotes | Bacteria | Gammaproteobacteria | spt | Salmonella enterica subsp. enterica serovar Paratyphi A ATCC9150 | 4091 | 0.533 |
| Eukaryotes | Animals | Echinoderms | spu | Strongylocentrotus purpuratus (purple sea urchin) | 28880 | 0.487 |
| Prokaryotes | Bacteria | Firmicutes | spv | Streptococcus pneumoniae Hungary19A 6 | 2155 | 0.406 |
| Prokaryotes | Bacteria | Firmicutes | spw | Streptococcus pneumoniae CGSP14 (serotype 14) | 2206 | 0.404 |
| Prokaryotes | Bacteria | Firmicutes | spx | Streptococcus pneumoniae G54 (serotype 19F) | 2114 | 0.407 |
| Prokaryotes | Bacteria | Firmicutes | spy | Streptococcus pyogenes SF370 (serotype M1) | 1696 | 0.394 |
| Prokaryotes | Bacteria | Firmicutes | spz | Streptococcus pyogenes MGAS5005 (serotype M1) | 1865 | 0.394 |
| Prokaryotes | Bacteria | Bacteroidetes | srm | Salinibacter ruber | 3186 | 0.665 |
| Prokaryotes | Bacteria | Actinobacteria | sro | Streptosporangium roseum | 8975 | 0.713 |
| Prokaryotes | Bacteria | Actinobacteria | srt | Segniliparus rotundus | 3006 | 0.672 |
| Prokaryotes | Bacteria | Bacteroidetes | sru | Salinibacter ruber | 2833 | 0.668 |
| Prokaryotes | Bacteria | Firmicutes | ssa | Streptococcus sanguinis | 2270 | 0.445 |
| Prokaryotes | Bacteria | Firmicutes | ssb | Streptococcus suis BM407 | 1947 | 0.419 |
| Eukaryotes | Animals | Vertebrates | ssc | Sus scrofa (pig) | 19125 | 0.539 |
| Prokaryotes | Bacteria | Firmicutes | ssd | Staphylococcus pseudintermedius | 2450 | 0.384 |
| Prokaryotes | Bacteria | Gammaproteobacteria | sse | Shewanella sediminis | 4497 | 0.474 |
| Prokaryotes | Bacteria | Firmicutes | ssg | Selenomonas sputigena | 2255 | 0.582 |
| Prokaryotes | Bacteria | Firmicutes | ssi | Streptococcus suis P1/7 | 1824 | 0.421 |
| Eukaryotes | Fungi | Ascomycetes | ssl | Sclerotinia sclerotiorum | 14446 | 0.457 |
| Prokaryotes | Bacteria | Spirochaetes | ssm | Spirochaeta smaragdinae | 4219 | 0.495 |
| Prokaryotes | Bacteria | Gammaproteobacteria | ssn | Shigella sonnei | 4470 | 0.520 |
| Prokaryotes | Archaea | Crenarchaeota | sso | Sulfolobus solfataricus | 2978 | 0.366 |
| Prokaryotes | Bacteria | Firmicutes | ssp | Staphylococcus saprophyticus | 2514 | 0.342 |
| Prokaryotes | Bacteria | Firmicutes | sss | Streptococcus suis SC84 | 1898 | 0.420 |
| Prokaryotes | Bacteria | Firmicutes | sst | Streptococcus suis ST3 | 1952 | 0.422 |
| Prokaryotes | Bacteria | Firmicutes | ssu | Streptococcus suis 05ZYH33 | 2186 | 0.420 |
| Prokaryotes | Bacteria | Firmicutes | ssv | Streptococcus suis 98HAH33 | 2185 | 0.420 |
| Prokaryotes | Bacteria | Firmicutes | stc | Streptococcus thermophilus CNRZ1066 | 1915 | 0.401 |
| Prokaryotes | Bacteria | Firmicutes | ste | Streptococcus thermophilus LMD-9 | 1715 | 0.401 |
| Prokaryotes | Bacteria | Firmicutes | sth | Symbiobacterium thermophilum | 3337 | 0.692 |
| Prokaryotes | Bacteria | Green nonsulfur bacteria | sti | Sphaerobacter thermophilus | 3485 | 0.683 |
| Prokaryotes | Bacteria | Firmicutes | stl | Streptococcus thermophilus LMG18311 | 1888 | 0.402 |
| Prokaryotes | Bacteria | Gammaproteobacteria | stm | Salmonella enterica subsp. enterica serovar Typhimurium LT2 | 4525 | 0.534 |
| Prokaryotes | Archaea | Crenarchaeota | sto | Sulfolobus tokodaii | 2826 | 0.337 |
| Prokaryotes | Bacteria | Actinobacteria | stp | Salinispora tropica | 4536 | 0.698 |
| Prokaryotes | Bacteria | Fusobacteria | str | Sebaldella termitidis | 4150 | 0.347 |
| Prokaryotes | Bacteria | Gammaproteobacteria | stt | Salmonella enterica subsp. enterica serovar Typhi Ty2 | 4313 | 0.533 |
| Prokaryotes | Bacteria | Gammaproteobacteria | sty | Salmonella enterica subsp. enterica serovar Typhi CT18 | 4753 | 0.530 |
| Prokaryotes | Bacteria | Firmicutes | sub | Streptococcus uberis | 1762 | 0.373 |
| Prokaryotes | Bacteria | Hyperthermophilic bacteria | sul | Sulfurihydrogenibium sp. YO3AOP1 | 1721 | 0.322 |
| Prokaryotes | Bacteria | Bacteroidetes | sum | Candidatus Sulcia muelleri CARI | 246 | 0.215 |
| Prokaryotes | Bacteria | Epsilonproteobacteria | sun | Sulfurovum sp. NBC37-1 | 2438 | 0.447 |
| Prokaryotes | Bacteria | Deltaproteobacteria | sur | Stigmatella aurantiaca | 8352 | 0.677 |
| Prokaryotes | Bacteria | Acidobacteria | sus | Solibacter usitatus | 7826 | 0.624 |
| Prokaryotes | Bacteria | Actinobacteria | svi | Saccharomonospora viridis | 3828 | 0.677 |
| Prokaryotes | Bacteria | Gammaproteobacteria | svo | Shewanella violacea | 4346 | 0.460 |
| Prokaryotes | Bacteria | Gammaproteobacteria | swd | Shewanella woodyi ATCC 51908 | 4880 | 0.448 |
| Prokaryotes | Bacteria | Alphaproteobacteria | swi | Sphingomonas wittichii | 5345 | 0.684 |
| Prokaryotes | Bacteria | Firmicutes | swo | Syntrophomonas wolfei | 2504 | 0.458 |
| Prokaryotes | Bacteria | Gammaproteobacteria | swp | Shewanella piezotolerans WP3 | 4911 | 0.442 |
| Prokaryotes | Bacteria | Cyanobacteria | syc | Synechococcus elongatus PCC6301 | 2527 | 0.561 |
| Prokaryotes | Bacteria | Cyanobacteria | syd | Synechococcus sp. CC9605 | 2645 | 0.602 |
| Prokaryotes | Bacteria | Cyanobacteria | sye | Synechococcus sp. CC9902 | 2306 | 0.549 |
| Prokaryotes | Bacteria | Cyanobacteria | syf | Synechococcus elongatus PCC7942 | 2662 | 0.560 |
| Prokaryotes | Bacteria | Cyanobacteria | syg | Synechococcus sp. CC9311 | 2892 | 0.534 |
| Prokaryotes | Bacteria | Cyanobacteria | syn | Synechocystis sp. PCC6803 | 3575 | 0.482 |
| Prokaryotes | Bacteria | Cyanobacteria | syp | Synechococcus sp. PCC7002 | 3187 | 0.503 |
| Prokaryotes | Bacteria | Cyanobacteria | syr | Synechococcus sp. RCC307 | 2534 | 0.612 |
| Prokaryotes | Bacteria | Cyanobacteria | syw | Synechococcus sp. WH8102 | 2519 | 0.602 |
| Prokaryotes | Bacteria | Cyanobacteria | syx | Synechococcus sp. WH7803 | 2533 | 0.606 |
| Prokaryotes | Archaea | Euryarchaeota | tac | Thermoplasma acidophilum | 1484 | 0.473 |
| Eukaryotes | Animals | Placozoans | tad | Trichoplax adhaerens | 11538 | 0.377 |
| Prokaryotes | Bacteria | Hyperthermophilic bacteria | taf | Thermosipho africanus | 1954 | 0.310 |
| Prokaryotes | Archaea | Crenarchaeota | tag | Thermosphaera aggregans | 1387 | 0.473 |
| Prokaryotes | Bacteria | Synergistetes | tai | Thermanaerovibrio acidaminovorans | 1738 | 0.643 |
| Prokaryotes | Bacteria | Hyperthermophilic bacteria | tal | Thermocrinis albus | 1593 | 0.471 |
| Prokaryotes | Bacteria | Hyperthermophilic bacteria | tam | Thermovibrio ammonificans | 1813 | 0.524 |
| Eukaryotes | Protists | Alveolates | tan | Theileria annulata | 3795 | 0.357 |
| Prokaryotes | Bacteria | Gammaproteobacteria | tau | Tolumonas auensis | 3130 | 0.498 |
| Prokaryotes | Archaea | Euryarchaeota | tba | Thermococcus barophilus | 2265 | 0.422 |
| Prokaryotes | Bacteria | Betaproteobacteria | tbd | Thiobacillus denitrificans | 2827 | 0.663 |
| Prokaryotes | Bacteria | Spirochaetes | tbe | Treponema brennaborense | 2531 | 0.520 |
| Prokaryotes | Bacteria | Actinobacteria | tbi | Thermobispora bispora | 3546 | 0.724 |
| Prokaryotes | Bacteria | Firmicutes | tbo | Thermoanaerobacter brockii | 2209 | 0.349 |
| Eukaryotes | Protists | Euglenozoa | tbr | Trypanosoma brucei | 8712 | 0.508 |
| Eukaryotes | Animals | Arthropods | tca | Tribolium castaneum (red flour beetle) | 9823 | 0.471 |
| Eukaryotes | Protists | Euglenozoa | tcr | Trypanosoma cruzi | 19602 | 0.532 |
| Prokaryotes | Bacteria | Actinobacteria | tcu | Thermomonospora curvata | 4890 | 0.721 |
| Prokaryotes | Bacteria | Gammaproteobacteria | tcx | Thiomicrospira crunogena | 2196 | 0.440 |
| Prokaryotes | Bacteria | Spirochaetes | tde | Treponema denticola | 2767 | 0.384 |
| Prokaryotes | Bacteria | Epsilonproteobacteria | tdn | Sulfurimonas denitrificans | 2096 | 0.349 |
| Prokaryotes | Bacteria | Cyanobacteria | tel | Thermosynechococcus elongatus | 2476 | 0.545 |
| Prokaryotes | Bacteria | Betaproteobacteria | teq | Taylorella equigenitalis | 1556 | 0.379 |
| Prokaryotes | Bacteria | Cyanobacteria | ter | Trichodesmium erythraeum | 4451 | 0.366 |
| Eukaryotes | Protists | Alveolates | tet | Tetrahymena thermophila | 24770 | 0.277 |
| Prokaryotes | Bacteria | Firmicutes | tex | Thermoanaerobacter sp. X514 | 2349 | 0.349 |
| Prokaryotes | Bacteria | Actinobacteria | tfu | Thermobifida fusca | 3087 | 0.681 |
| Prokaryotes | Archaea | Euryarchaeota | tga | Thermococcus gammatolerans | 2156 | 0.542 |
| Eukaryotes | Protists | Alveolates | tgo | Toxoplasma gondii | 7987 | 0.581 |
| Prokaryotes | Bacteria | Gammaproteobacteria | tgr | Thioalkalivibrio sp. HL-EbGR7 | 3283 | 0.661 |
| Eukaryotes | Animals | Vertebrates | tgu | Taeniopygia guttata (zebra finch) | 13694 | 0.513 |
| Prokaryotes | Bacteria | Betaproteobacteria | tin | Thiomonas intermedia | 3172 | 0.644 |
| Prokaryotes | Bacteria | Firmicutes | tit | Thermoanaerobacter italicus | 2270 | 0.345 |
| Prokaryotes | Bacteria | Firmicutes | tjr | Thermincola potens JR | 2949 | 0.467 |
| Prokaryotes | Bacteria | Gammaproteobacteria | tkm | Thioalkalivibrio sp. K90mix | 2855 | 0.659 |
| Prokaryotes | Archaea | Euryarchaeota | tko | Thermococcus kodakaraensis | 2306 | 0.528 |
| Prokaryotes | Bacteria | Hyperthermophilic bacteria | tle | Thermotoga lettingae | 2040 | 0.388 |
| Prokaryotes | Bacteria | Hyperthermophilic bacteria | tma | Thermotoga maritima | 1858 | 0.465 |
| Prokaryotes | Bacteria | Hyperthermophilic bacteria | tme | Thermosipho melanesiensis | 1879 | 0.316 |
| Eukaryotes | Fungi | Ascomycetes | tml | Tuber melanosporum | 7496 | 0.514 |
| Prokaryotes | Bacteria | Firmicutes | tmr | Thermaerobacter marianensis | 2327 | 0.724 |
| Prokaryotes | Bacteria | Firmicutes | tmt | Thermoanaerobacter mathranii | 2159 | 0.347 |
| Prokaryotes | Bacteria | Betaproteobacteria | tmz | Thauera sp. MZ1T | 3978 | 0.686 |
| Prokaryotes | Bacteria | Hyperthermophilic bacteria | tna | Thermotoga neapolitana | 1937 | 0.472 |
| Prokaryotes | Archaea | Crenarchaeota | tne | Thermoproteus neutrophilus | 1966 | 0.604 |
| Prokaryotes | Bacteria | Hyperthermophilic bacteria | tnp | Thermotoga naphthophila | 1768 | 0.464 |
| Prokaryotes | Bacteria | Firmicutes | tnr | Thermodesulfobium narugense | 1807 | 0.344 |
| Prokaryotes | Bacteria | Firmicutes | toc | Thermosediminibacter oceani | 2197 | 0.478 |
| Prokaryotes | Archaea | Euryarchaeota | ton | Thermococcus onnurineus | 1975 | 0.522 |
| Prokaryotes | Bacteria | Spirochaetes | tpa | Treponema pallidum subsp. pallidum Nichols | 1036 | 0.526 |
| Prokaryotes | Bacteria | Firmicutes | tpd | Thermoanaerobacter pseudethanolicus | 2243 | 0.349 |
| Prokaryotes | Archaea | Crenarchaeota | tpe | Thermofilum pendens | 1878 | 0.583 |
| Prokaryotes | Bacteria | Spirochaetes | tpp | Treponema pallidum subsp. pallidum SS14 | 1028 | 0.526 |
| Prokaryotes | Bacteria | Actinobacteria | tpr | Tsukamurella paurometabola | 4242 | 0.687 |
| Eukaryotes | Protists | Diatoms | tps | Thalassiosira pseudonana | 11672 | 0.479 |
| Prokaryotes | Bacteria | Hyperthermophilic bacteria | tpt | Thermotoga petrophila | 1785 | 0.463 |
| Eukaryotes | Protists | Alveolates | tpv | Theileria parva | 4061 | 0.378 |
| Prokaryotes | Bacteria | Deinococcus | tra | Truepera radiovictrix | 2945 | 0.686 |
| Prokaryotes | Bacteria | Green nonsulfur bacteria | tro | Thermomicrobium roseum | 2854 | 0.642 |
| Prokaryotes | Bacteria | Hyperthermophilic bacteria | trq | Thermotoga sp. RQ2 | 1819 | 0.464 |
| Prokaryotes | Bacteria | Acidobacteria | tsa | Terriglobus saanensis | 4180 | 0.579 |
| Prokaryotes | Bacteria | Deinococcus | tsc | Thermus scotoductus | 2461 | 0.651 |
| Prokaryotes | Archaea | Euryarchaeota | tsi | Thermococcus sibiricus | 2035 | 0.408 |
| Prokaryotes | Bacteria | Spirochaetes | tsu | Treponema succinifaciens | 2608 | 0.397 |
| Prokaryotes | Bacteria | Firmicutes | tte | Thermoanaerobacter tengcongensis | 2588 | 0.380 |
| Prokaryotes | Bacteria | Deinococcus | tth | Thermus thermophilus HB27 | 2210 | 0.696 |
| Prokaryotes | Bacteria | Deinococcus | ttj | Thermus thermophilus HB8 | 2238 | 0.696 |
| Prokaryotes | Bacteria | Firmicutes | ttm | Thermoanaerobacterium thermosaccharolyticum | 2601 | 0.348 |
| Prokaryotes | Bacteria | Hyperthermophilic bacteria | ttr | Thermobaculum terrenum | 2832 | 0.539 |
| Prokaryotes | Bacteria | Gammaproteobacteria | ttu | Teredinibacter turnerae | 4254 | 0.517 |
| Eukaryotes | Protists | Parabasalids | tva | Trichomonas vaginalis | 59679 | 0.355 |
| Prokaryotes | Archaea | Euryarchaeota | tvo | Thermoplasma volcanium | 1501 | 0.412 |
| Prokaryotes | Bacteria | Actinobacteria | twh | Tropheryma whipplei Twist | 808 | 0.466 |
| Prokaryotes | Bacteria | Actinobacteria | tws | Tropheryma whipplei TW08/27 | 783 | 0.465 |
| Prokaryotes | Bacteria | Hyperthermophilic bacteria | tye | Thermodesulfovibrio yellowstonii | 2033 | 0.343 |
| Eukaryotes | Fungi | Basidiomycetes | uma | Ustilago maydis | 6538 | 0.561 |
| Prokaryotes | Bacteria | Tenericutes | upa | Ureaplasma parvum serovar 3 ATCC 27815 | 609 | 0.260 |
| Eukaryotes | Fungi | Ascomycetes | ure | Uncinocarpus reesii | 7760 | 0.512 |
| Prokaryotes | Bacteria | Tenericutes | uue | Ureaplasma urealyticum serovar 10 ATCC 33699 | 646 | 0.263 |
| Prokaryotes | Bacteria | Tenericutes | uur | Ureaplasma parvum serovar 3 ATCC 700970 | 614 | 0.261 |
| Prokaryotes | Bacteria | Betaproteobacteria | vap | Variovorax paradoxus S110 | 6279 | 0.681 |
| Prokaryotes | Bacteria | Gammaproteobacteria | vch | Vibrio cholerae O1 | 3834 | 0.482 |
| Prokaryotes | Bacteria | Gammaproteobacteria | vcj | Vibrio cholerae MJ-1236 | 3772 | 0.482 |
| Prokaryotes | Bacteria | Gammaproteobacteria | vcm | Vibrio cholerae M66-2 | 3693 | 0.483 |
| Eukaryotes | Plants | Green algae | vcn | Volvox carteri f. nagariensis | 14434 | 0.630 |
| Prokaryotes | Bacteria | Gammaproteobacteria | vco | Vibrio cholerae O395 | 3875 | 0.483 |
| Prokaryotes | Archaea | Crenarchaeota | vdi | Vulcanisaeta distributa | 2493 | 0.464 |
| Prokaryotes | Bacteria | Betaproteobacteria | vei | Verminephrobacter eiseniae | 4947 | 0.655 |
| Prokaryotes | Bacteria | Gammaproteobacteria | vex | Vibrio sp. Ex25 | 4518 | 0.459 |
| Prokaryotes | Bacteria | Gammaproteobacteria | vfi | Vibrio fischeri | 3817 | 0.392 |
| Prokaryotes | Bacteria | Gammaproteobacteria | vfm | Vibrio fischeri MJ11 | 4039 | 0.389 |
| Prokaryotes | Bacteria | Gammaproteobacteria | vha | Vibrio harveyi | 6039 | 0.463 |
| Prokaryotes | Bacteria | Actinobacteria | vma | Verrucosispora maris | 6009 | 0.712 |
| Prokaryotes | Archaea | Crenarchaeota | vmo | Vulcanisaeta moutnovskia | 2320 | 0.433 |
| Prokaryotes | Bacteria | Gammaproteobacteria | vok | Candidatus Vesicomyosocius okutanii | 937 | 0.330 |
| Prokaryotes | Bacteria | Gammaproteobacteria | vpa | Vibrio parahaemolyticus | 4832 | 0.464 |
| Prokaryotes | Bacteria | Betaproteobacteria | vpe | Variovorax paradoxus EPS | 5952 | 0.670 |
| Eukaryotes | Fungi | Ascomycetes | vpo | Vanderwaltozyma polyspora | 5336 | 0.349 |
| Prokaryotes | Bacteria | Firmicutes | vpr | Veillonella parvula | 1844 | 0.398 |
| Prokaryotes | Bacteria | Gammaproteobacteria | vsa | Aliivibrio salmonicida LFI1238 | 3911 | 0.398 |
| Prokaryotes | Bacteria | Gammaproteobacteria | vsp | Vibrio splendidus | 4432 | 0.450 |
| Eukaryotes | Plants | Eudicots | vvi | Vitis vinifera (wine grape) | 22511 | 0.451 |
| Prokaryotes | Bacteria | Gammaproteobacteria | vvm | Vibrio vulnificus MO6-24/O | 4562 | 0.479 |
| Prokaryotes | Bacteria | Gammaproteobacteria | vvu | Vibrio vulnificus CMCP6 | 4433 | 0.477 |
| Prokaryotes | Bacteria | Gammaproteobacteria | vvy | Vibrio vulnificus YJ016 | 5024 | 0.476 |
| Prokaryotes | Bacteria | Alphaproteobacteria | wbm | Wolbachia wBm | 805 | 0.352 |
| Prokaryotes | Bacteria | Gammaproteobacteria | wbr | Wigglesworthia glossinidia | 617 | 0.241 |
| Prokaryotes | Bacteria | Chlamydiae | wch | Waddlia chondrophila | 1956 | 0.443 |
| Prokaryotes | Bacteria | Alphaproteobacteria | wol | Wolbachia wMel | 1195 | 0.355 |
| Prokaryotes | Bacteria | Alphaproteobacteria | wpi | Wolbachia pipientis | 1275 | 0.348 |
| Prokaryotes | Bacteria | Alphaproteobacteria | wri | Wolbachia sp. wRi | 1150 | 0.355 |
| Prokaryotes | Bacteria | Epsilonproteobacteria | wsu | Wolinella succinogenes | 2043 | 0.489 |
| Prokaryotes | Bacteria | Bacteroidetes | wvi | Weeksella virosa | 2049 | 0.368 |
| Prokaryotes | Bacteria | Gammaproteobacteria | xac | Xanthomonas axonopodis | 4427 | 0.650 |
| Prokaryotes | Bacteria | Gammaproteobacteria | xal | Xanthomonas albilineans | 3114 | 0.634 |
| Prokaryotes | Bacteria | Alphaproteobacteria | xau | Xanthobacter autotrophicus | 5035 | 0.678 |
| Prokaryotes | Bacteria | Gammaproteobacteria | xbo | Xenorhabdus bovienii | 4260 | 0.464 |
| Prokaryotes | Bacteria | Gammaproteobacteria | xca | Xanthomonas campestris pv. campestris B100 | 4466 | 0.654 |
| Prokaryotes | Bacteria | Gammaproteobacteria | xcb | Xanthomonas campestris pv. campestris 8004 | 4271 | 0.654 |
| Prokaryotes | Bacteria | Gammaproteobacteria | xcc | Xanthomonas campestris pv. campestris ATCC 33913 | 4179 | 0.656 |
| Prokaryotes | Bacteria | Actinobacteria | xce | Xylanimonas cellulosilytica | 3443 | 0.726 |
| Prokaryotes | Bacteria | Gammaproteobacteria | xcv | Xanthomonas campestris pv. vesicatoria | 4726 | 0.648 |
| Prokaryotes | Bacteria | Gammaproteobacteria | xfa | Xylella fastidiosa 9a5c | 2832 | 0.538 |
| Prokaryotes | Bacteria | Gammaproteobacteria | xfm | Xylella fastidiosa M12 | 2104 | 0.533 |
| Prokaryotes | Bacteria | Gammaproteobacteria | xfn | Xylella fastidiosa M23 | 2201 | 0.532 |
| Prokaryotes | Bacteria | Gammaproteobacteria | xft | Xylella fastidiosa Temecula1 | 2036 | 0.533 |
| Eukaryotes | Animals | Vertebrates | xla | Xenopus laevis (African clawed frog) | 11013 | 0.468 |
| Prokaryotes | Bacteria | Gammaproteobacteria | xne | Xenorhabdus nematophila | 4474 | 0.456 |
| Prokaryotes | Bacteria | Gammaproteobacteria | xom | Xanthomonas oryzae MAFF311018 | 4372 | 0.642 |
| Prokaryotes | Bacteria | Gammaproteobacteria | xoo | Xanthomonas oryzae KACC10331 | 4064 | 0.642 |
| Prokaryotes | Bacteria | Gammaproteobacteria | xop | Xanthomonas oryzae PXO99A | 4988 | 0.642 |
| Eukaryotes | Animals | Vertebrates | xtr | Xenopus tropicalis (western clawed frog) | 22304 | 0.470 |
| Prokaryotes | Bacteria | Gammaproteobacteria | yen | Yersinia enterocolitica subsp. enterocolitica 8081 | 4050 | 0.484 |
| Prokaryotes | Bacteria | Gammaproteobacteria | yep | Yersinia enterocolitica subsp. palearctica 105.5R(r) | 4021 | 0.481 |
| Eukaryotes | Fungi | Ascomycetes | yli | Yarrowia lipolytica | 6472 | 0.536 |
| Prokaryotes | Bacteria | Gammaproteobacteria | ypa | Yersinia pestis Antiqua (biovar Antiqua) | 4361 | 0.490 |
| Prokaryotes | Bacteria | Gammaproteobacteria | ypb | Yersinia pseudotuberculosis PB1/+ | 4237 | 0.488 |
| Prokaryotes | Bacteria | Gammaproteobacteria | ype | Yersinia pestis CO92 (biovar Orientalis) | 4066 | 0.489 |
| Prokaryotes | Bacteria | Gammaproteobacteria | ypg | Yersinia pestis Angola | 4039 | 0.489 |
| Prokaryotes | Bacteria | Gammaproteobacteria | ypi | Yersinia pseudotuberculosis IP31758 (serotype O:1b) | 4324 | 0.485 |
| Prokaryotes | Bacteria | Gammaproteobacteria | ypk | Yersinia pestis KIM 10 (biovar Mediaevalis) | 4165 | 0.490 |
| Prokaryotes | Bacteria | Gammaproteobacteria | ypm | Yersinia pestis 91001 (biovar Microtus) | 4137 | 0.489 |
| Prokaryotes | Bacteria | Gammaproteobacteria | ypn | Yersinia pestis Nepal516 (biovar Antiqua) | 4091 | 0.489 |
| Prokaryotes | Bacteria | Gammaproteobacteria | ypp | Yersinia pestis Pestoides F | 4068 | 0.491 |
| Prokaryotes | Bacteria | Gammaproteobacteria | yps | Yersinia pseudotuberculosis IP32953 (serotype I) | 4038 | 0.489 |
| Prokaryotes | Bacteria | Gammaproteobacteria | ypy | Yersinia pseudotuberculosis YPIII | 4192 | 0.488 |
| Prokaryotes | Bacteria | Gammaproteobacteria | ypz | Yersinia pestis Z176003 | 3695 | 0.491 |
| Prokaryotes | Bacteria | Betaproteobacteria | zin | Candidatus Zinderia insecticola CARI | 202 | 0.141 |
| Eukaryotes | Plants | Monocots | zma | Zea mays (maize) | 22152 | 0.573 |
| Prokaryotes | Bacteria | Alphaproteobacteria | zmn | Zymomonas mobilis subsp. mobilis NCIMB 11163 | 1884 | 0.481 |
| Prokaryotes | Bacteria | Alphaproteobacteria | zmo | Zymomonas mobilis | 1736 | 0.478 |
| Prokaryotes | Bacteria | Bacteroidetes | zpr | Zunongwangia profunda | 4653 | 0.371 |
| Eukaryotes | Fungi | Ascomycetes | zro | Zygosaccharomyces rouxii | 4991 | 0.400 |

Figure S1 – Histogram of the shape parameter in the modelled gamma functions.

The distribution of the shape parameter values obtained in the modelled gamma functions

Figure S2 – Dendogram of protein size attributes

Dendogram of protein size attributes in different species. Data from table 1 was used to construct a distance matrix for hierarchical clustering. Euclidean distances were calculated and then full hierarchical clustering was plotted with default parameters of the R function hclust(dist(data)).

Figure S3– Pareto´s best fit of the right handed distribution tail

Pareto’s best fit for *Arabidopsis thaliana*
